# Adipocyte RNF20 Knockout Leads to Hyperinsulinemia via the H2Bub‐H3K4me3‐*Slc2a4* Axis

**DOI:** 10.1111/jcmm.70649

**Published:** 2025-06-16

**Authors:** Ying Zhao, Xiaojuan Liang, Jiayu Tang, Chunwei Cao, Chunhuai Yang, Shulin Yang, Jianguo Zhao, Jinxiang Yuan, Meng Zhang, Yanfang Wang

**Affiliations:** ^1^ State Key Laboratory of Animal Biotech Breeding Institute of Animal Science, Chinese Academy of Agricultural Sciences Beijing China; ^2^ Guangdong Provincial Key Laboratory of Malignant Tumor Epigenetics and Gene Regulation, Guangdong‐Hong Kong Joint Laboratory for RNA Medicine Sun Yat‐Sen Memorial Hospital, Sun Yat‐Sen University Guangzhou China; ^3^ State Key Laboratory of Stem Cell and Reproductive Biology Institute of Zoology, Chinese Academy of Sciences Beijing China; ^4^ Savaid Medical School University of Chinese Academy of Sciences Beijing China; ^5^ Sanya Institute, Hainan Academy of Agricultural Sciences Sanya China; ^6^ Lin He's Academician Workstation of New Medicine and Clinical Translation Jining Medical University Jining Shandong China; ^7^ College of Medical Imaging and Laboratory Jining Medical University Jining Shandong China

**Keywords:** adipose tissue, H3K4me3, insulin resistance, RNF20, *Slc2a4*

## Abstract

The ubiquitin ligase RING finger 20 (RNF20) mediated the monoubiquitination of histone H2B at lysine 120 (H2Bub), an epigenetic modification known to regulate key biological processes such as fat tissue development, tumorigenesis, spermatogenesis and so on. Despite our previous findings showing that mice with adipocyte‐specific deletion of *Rnf20* (ASKO mice) develop hyperinsulinaemia, the underlying mechanisms remain unclear. In this study, we investigated the role of adipocyte RNF20 in maintaining systemic insulin homoeostasis in ASKO mice. Our results reveal that ASKO mice exhibit an enlarged pancreas, increased islet size and a greater number of pancreatic β‐cells. Fat tissue in ASKO mice showed reduced insulin sensitivity, evidenced by diminished AKT phosphorylation under basal and insulin‐stimulated conditions, alongside suppressed insulin signalling pathways. Furthermore, the decreased levels of histone modifications, including H2Bub, H3K4me3 and H3K79me3, were observed in both ASKO mice fat tissues and *Rnf20*‐knockdown 3T3‐L1 cells. Mechanistically, *Rnf20* knockdown in adipocytes reduced H3K4me3 occupancy at the *Slc2a4* gene locus, inhibiting GLUT4 expression and inducing adipose‐specific insulin resistance. These findings establish a critical role for adipocyte RNF20 in the insulin signalling regulation via the H2Bub‐H3K4me3‐*Slc2a4* axis, highlighting its importance in systemic glucose metabolism.

## 1 Introduction

Accumulation of an excessive amount of body fat causes type 2 diabetes (T2D), which is characterised by glucose intolerance and insulin resistance (IR), also accompanied by the disorder of blood glucose homoeostasis and hyperinsulinemia [1]. There is mounting evidence revealing a close relationship between hyperinsulinaemia and IR. At the physiological level, IR is a condition where the cellular insulin response is impaired, resulting in elevated circulating insulin levels under both fasted and fed states (also called hyperinsulinaemia). Meanwhile, hyperinsulinaemia further exacerbates the metabolic triad of insulin resistance, hyperglycaemia and hypertriglyceridemia [2]. To summarise, hyperinsulinaemia is often both a result and a driver of insulin resistance. Accumulating evidence demonstrates that the imbalance of the phosphatidylinositol 3‐kinase (PI3K)/protein kinase B (also named AKT) signalling pathway leads to the development of obesity, T2D and insulin resistance [3]. This pathway includes insulin receptor (INSR), insulin receptor substrate (IRS), PI3K and AKT proteins [4]. Notably, AKT phosphorylation sites of Thr308 and Ser473 are obligatory for its activation and the hallmarks of insulin resistance [5, 6].

Some of the insights emerging from clinical data and animal experiments point to the importance of the epigenetic mechanism in maintaining glucose and insulin homoeostasis both in humans and mice. Healthy individuals and insulin‐resistant patients showed a negative correlation between the levels of several histone modifications (H3K4me3 and H3K9/14 ac) in adipose tissues and the homoeostatic model assessment of insulin resistance (HOMA‐IR) index, which is a clinical homoeostatic model assessment for insulin resistance [7]. The clinical data showed that the epigenetic pathways related to regulating insulin action were marked by H3K4me3 occupancy in insulin‐resistant patients [8]. Histone deacetylase (HDAC) is an important epigenetic regulator, and its activity is significantly and positively correlated with HOMA‐IR in both males and females [9]. Mice with overfeeding found that the up‐regulated expression of monoacylglycerol *O*‐acyltransferase (*Mogat1*) conceivably mediates insulin resistance, accompanied by the occupancies of H3K4me3, H3K27me3 and H3K9ac, which were significantly increased in the promoter of the *Mogat1* gene [10]. In vitro studies, the H3K4 hypertrimethylation and H3, H4 hyperacetylation levels were increased in the fatty acid synthesis (*Fasn*) gene promoter in HepG2 cells and primary hepatocytes following insulin stimulation [11]. HDAC8 directly regulated the expression level of sterol regulatory element‐binding protein‐1 (SREBP‐1), a master regulator of *de novo* lipogenesis that contributes to glucose homoeostasis [12]. Overall, these studies suggested a close relationship between the histone modification levels and the maintenance of insulin homoeostasis.

Ring finger 20 (RNF20) is an E3 ubiquitination ligase that monoubiquitinates histone H2B at lysine 120 (H2Bub) and is an important epigenetic modification for cellular events. H2Bub physiologically disrupts the compaction of chromatin, resulting in a more open conformation through recruiting the transcription factors and other protein complexes that participate in transcriptional activation [13]. On the other hand, RNF20/RNF40 axis‐driven H2Bub promotes the broadening of H3K4me3 peaks to facilitate tissue‐specific gene transcription [14]. For example, the H2UB1‐mediated histone H2Bub and H3K4me3 marks were associated with the circadian clock genes during periodic and inducible processes such as the cell cycle, dormancy, flowering time and defence responses [15]. The intestine‐specific of *Rnf20* or *Rnf40* resulted in spontaneous colorectal inflammation through the deregulation of the IBD‐related genes and the inhibition of vitamin D receptor (*Vdr*) activity via H2Bub‐H3K4me3 [16]. RNAi‐mediated knockdown of RNF20 lowers both the levels of H2Bub and H3K4me3, which is repressive of inducible transcription at the *Irf1* gene locus [17]. However, the detailed regulatory mechanism of RNF20 and its mediated histone modifications on glucose homoeostasis remains unclear.

Our previous studies showed that mice with adipocyte‐specific deletion of the *Rnf20* gene (ASKO mice) suffered hyperinsulinaemia, as evidenced by the significantly elevated serum insulin content, compared to those from wild‐type mice [18], but the detailed molecular mechanisms were unknown. Here, we evaluated the pathological features of the pancreas of ASKO mice by histological and insulin immunohistochemistry analysis. RNA‐seq and ChIP‐Seq combination analysis were utilised to explore the effect of adipocyte *Rnf20* on the regulation of systemic insulin homoeostasis, providing new insight into the role of RNF20 in hyperinsulinaemia.

## 2 Materials and Methods

### 2.1 Mice and Animal Experiments

Animal experiments were approved by the Animal Research Panel of the Committee on Research Practice of the University of the Chinese Academy of Sciences (No. IOZ20190077). *Rnf20* ^flox/flox^ mice (referred to as WT mice) and *Rnf20* ^flox/flox^ adiponectin‐Cre mice (referred to as ASKO mice) were generated as described before [18]. All male mice (C57BL6/J background) were kept at 25°C on a 12 h light/12 h dark cycle with *ad libitum* access to food and water.

For biochemical analysis, 6‐month‐old WT and ASKO male mice were fasted for at least 12 h (h), and then serum from the two groups of mice was collected for detection of the level of insulin by using the Mouse Insulin ELISA kit (Abcam, ab277390).

To evaluate peripheral tissue insulin delivery and signalling to AKT, two groups of 6‐month‐old male mice were fasted for 4 h (from 9 am to 1 pm) and then injected intraperitoneally with 0.75 IU/kg insulin (Novolin^@^ R Penfill) or sterile saline. Ten minutes after injection, inguinal white adipose tissue (iWAT), gonadal white adipose tissue (gWAT), brown adipose tissue (BAT), gastrocnemius muscle and liver were excised and frozen in liquid nitrogen for further analysis.

### 2.2 Morphology of Pancreatic Tissue Structure

Two groups of male mice were sacrificed by cervical dislocation, and the pancreas tissues were collected. The pancreas index was calculated as pancreas wet weight (g)/body weight (g). Then, pancreas tissues were fixed in 4% paraformaldehyde (PFA) solution for two days, embedded in paraffin and sectioned. A serial section of one slice at an interval of 10 sections (5‐ μm) was collected for haematoxylin and eosin (H&E) staining. The morphology of pancreatic tissue was observed under an optical microscope (Nikon, Japan). Based on this assignment, we utilised Image J software (National Institute of Health, USA) to quantify the areas of individual islet clones (at least 50 islets per group).

For immunohistochemical staining, epitopes were unmasked by heat treatment in sodium citrate buffer (pH = 6.0) at 95°C for 20 min and then cooled to room temperature (RT). Sections were blocked with 5% bovine serum albumin (BSA, Solarbio, China) in PBST (0.1% Triton‐X‐100 in PBS) for 1 h, incubated with 3% hydrogen peroxide at 37°C for 15 min to inhibit endogenous peroxidase activity and then subsequently incubated overnight at 4°C in a 1:200 dilution of anti‐mouse monoclonal Insulin B (C‐12) antibody (sc‐377,071, Santa Cruz Biotechnology, USA). Subsequently, slides were incubated with ready‐to‐use undiluted secondary antibodies conjugated with biotin in the UltraSensitiveTM SP (mouse) IHC kit (KIT‐9701, Fuzhou, China) for 15 min at 37°C. Subsequently, 3,3′‐diaminobenzidine (DAB) staining (DA1010, Solarbio, China) was performed for 5 min at RT, and the nuclei were stained with haematoxylin (Beyotime, China) for 15 min at RT. The stained sections were observed under a light microscope (Nikon, Japan) and the percentage of insulin‐positive cells was quantified using Image J software.

### 2.3 Protein Extraction and Western Blotting

Western blotting was performed using our previous procedures [19]. Proteins were extracted from tissues using T‐PER Tissue Protein Extraction Reagent (Thermo Fisher Scientific, USA) buffer containing a protease and phosphatase inhibitor tablet (Roche, Switzerland) and then they were centrifuged for 15 min at 12,000 rpm to remove cell debris. The bicinchoninic acid (BCA) protein assay kit (Beyotime, Beijing) was used to determine the total protein concentration. Supernatants were prepared in 5 x Reducing Buffer (CWBIO, China), separated by SDS‐PAGE, and then transferred to nitrocellulose membranes (NC, Millipore, USA). Membranes with indicated proteins were blocked with 5% skim milk at RT for 2 h and incubated overnight at 4°C in primary antibodies. Then, blots were incubated using the anti‐rabbit IgG HRP‐linked secondary antibody (CST, 7074) and measured with enhanced chemiluminescence (ECL) using Tanon 5200 (Tanon, China).

Primary antibodies used in this study, including β‐Tubulin (CST, 2146), RNF20 (Proteintech, 21625–1‐1‐AP), H2Bub (CST, 5546), H3K4me3 (Abcam, ab8580), H3K79me3 (Abcam, ab177185), PPARγ (CST, 2443), C/EBPα (Abcam, ab40764), AKT (CST, 4691) and p‐AKT (Ser473) (CST, 9271).

### 2.4 RNA Extraction, Reverse Transcription, and qPCR

Quantitative reverse‐transcription PCR (qPCR) was performed as we described previously [19]. Total RNA was isolated using RNAiso (Takara, Japan) according to the manufacturer's introductions. After DNase treatment (RNase‐Free DNase Set, CWBIO, China), 1 μg of total RNA was reverse transcribed to cDNA by PrimeScript RT Reagent Kit (Takara, Japan) and random primers according to the manufacturer's instructions. Gene expression differences were determined by quantitative PCR using TB Green Premix EX Taq (Takara, Japan) on the Applied Biosystems Quant Studio 3 Real‐Time PCR System (Thermo Fisher Scientific, USA). Relative gene expression was calculated using the 2^‐ΔΔCT^ method; the *18 s* gene was used as a housekeeping gene. Primer sequences are listed in Table S1.

### 2.5 RNA‐Sequencing and Analysis

Total RNA was extracted from gWAT of 6‐month‐old WT and ASKO mice, four independent samples per group, the protocol used as we described before [20]. Samples were run on the Agilent 2100 Bioanalyzer to determine the level of degradation, thus ensuring only high‐quality RNA was used (OD 260/280 ≥ 2.0 and RIN > 8). High‐throughput sequencing was performed by HiSeq 2000 at the Next Generation Sequencing Core at Shanghai Personal Biotechnology Co. Ltd. (Shanghai, China).

Differentially expressed genes (DEGs) were selected with a cut‐off of fold change (FC) > 1.5 and *p*‐value < 0.05 using a two‐tailed Student's *t*‐test. Volcano plot and heatmap analysis of DEGs were generated using ‘ggplot2’ and ‘pheatmap’ packages, which were installed and used in R software (Version 3.5.3, USA). The DEGs were uploaded into DAVID Functional Annotation Bioinformatics Microarray Analysis (https://david.ncifcrf.gov/) for pathway analysis. Kyoto Encyclopedia of Genes and Genomes (KEGG) pathways with *p*‐value < 0.05 were regarded as statically significant. A complete list of the transcriptome data is provided in Table S2.

### 2.6 RNAi Experiment

Small interfering RNAs (siRNAs), including negative control siRNA (siNC) or siRNA‐targeting *Rnf20* (siRNF20), were separately transfected into 3T3‐L1 cells using Lipofectamine RNAiMAX Transfection Reagent (Invitrogen, USA) in Opti‐MEM reduced serum media (Gibco, USA) according to the manufacturer's protocols. The sequences of siNC and siRNF20 were shown in our previous study [18] and were synthesised from Gene Pharma (Shanghai, China).

### 2.7 Primary Culture of Mouse Preadipocytes

Mouse preadipocytes were harvested from the subcutaneous adipose tissues of 6‐week‐old WT and ASKO mice, minced and digested with 1 mg/mL collagenase type I (Sigma, USA) in D’Hanks (Solarbio, China). The cell suspension was filtered through a 70 μM filter and was centrifuged for 15 min at 200 x g to collect cell pellets. Then, the pellets were cultured in DMEM (Gibco, USA) plus 20% foetal bovine serum (FBS, PAN, USA) and 1% penicillin–streptomycin (P/S, Sigma, USA) for 24 h at 37°C, and the preadipocytes will adhere in 2–3 days. The medium was changed every 2 or 3 days.

### 2.8 Cell Differentiation

For 3T3‐L1 cells or preadipocytes differentiation, the cells were seeded in the six‐well plate with culture media DMEM (Gibco, USA) containing 10% FBS and 1% P/S. Two days post‐confluency, cells were treated with culture medium DMEM/F12 supplemented with 5 μg/mL insulin (Macgene, China), 0.5 mM 3‐isobutyl methylxanthine (IBMX, MCE, China), 1 μM dexamethasone (Dex, Sigma, USA) and 1 μM rosiglitazone (Rosi, Sigma, USA). After 4 days, cells were re‐fed with the culture medium containing 5 μg/mL insulin and were fully induced after 6–8 days.

### 2.9 Oil Red O Staining

Adipocyte differentiation was determined by oil red o (ORO) staining (Sigma, MA, USA). Cells were washed twice with DPBS and then fixed with 4% PFA (Solarbio, Beijing, China) for 30 min at RT or overnight at 4°C. Next, the working solution for ORO (oil red o: deionised water = 4:6) was made freshly, used within 2 h of preparation, and then kept away from light. Then, 60% of isopropanol was used to wash the cells. The cells were stained with working solution for 15–20 min at RT, and the overstaining was washed twice with DPBS. Finally, the cells were observed using a microscope (Nikon, Tokyo, Japan).

### 2.10 ChIP‐Sequencing and ChIP‐qPCR

3T3‐L1 cells transfected with siNC or siRNF20 and then differentiated into mature adipocytes. Then, cells were harvested and fixed with 1% formaldehyde (Alfa Aesar, USA) for 10 min and quenched with 0.125 M glycine (Solaribo, China) for 5 min at RT. Suspended cells were centrifuged at 1000 rpm for 5 min and pellets were washed twice with ice‐cold PBS.

ChIP‐Seq was performed by Wuhan Seqhealth Tech Co. Ltd. (Wuhan, China). Briefly, high throughput sequencing and analysis were constructed using DNBSEQ‐T7 sequencer (MGI Tech., China) with the PE150 model. Peaks were identified by MACS2 software (Version 2.1.1), and then peak annotation and peak distribution were analysed by Bedtools (Version 2.25.0). The primary analysis of ChIP‐seq were as follows: The threshold of the number of valid peaks was selected within a *p*‐value of 0.05. To define meaningful H3K4me3 binding sites, we defined the fold enrichment of a peak that was larger than 1.5 in IP versus input, and the FC of tag density was considered at least *p‐*value < 0.05. The ChIP‐Seq data are shown in Table S3.

For ChIP‐qPCR analysis, siNC and siRNF20 cell pellets were resuspended in buffer A plus protease inhibitor cocktail (PIC) and dithiothreitol (DTT) from Simple ChIP Enzymatic Chromatin IP kit (Magnetic Beads) (CST, 9003) according to the manufacturer's instruction. The cross‐linked chromatin was sheared into fragments of 200–1000 bp with a sonicator (XM08‐II, Shanghai, China). Then, 200 μg DNA from each sample was incubated with buffer A containing 2 μg Normal Rabbit Anti‐IgG HRP‐linked Antibody (CST, 7074) or Anti‐Histone H3 (trimethyl K4) ChIP Grade Antibody (Abcam, ab8580) at 4°C overnight. Bound chromatin was eluted and reverse–cross‐linked at 65°C for 4 h. ChIP DNA quantification was conducted by qPCR according to the manufacturer's instructions. As mentioned earlier, the enrichment is calculated as a percentage of 2% input. The sequences of ChIP‐qPCR primers were shown in Table S1.

### 2.11 Statistical Analysis

Statistical analyses were processed using GraphPad Prism 8.0.1 software (National Institutes of Health, USA). Significant differences were evaluated through an unpaired Student's *t*‐test (*p* value). All data are presented as means ± the standard error of the mean (SEM). In all cases, the statistical significance of the difference between groups was defined as *p < 0.05, **p < 0.01 and ***p < 0.001 versus controls.

## 3 Results

### 3.1 ASKO Mice Displayed Decreased Systemic Insulin Signalling

**FIGURE 1:**
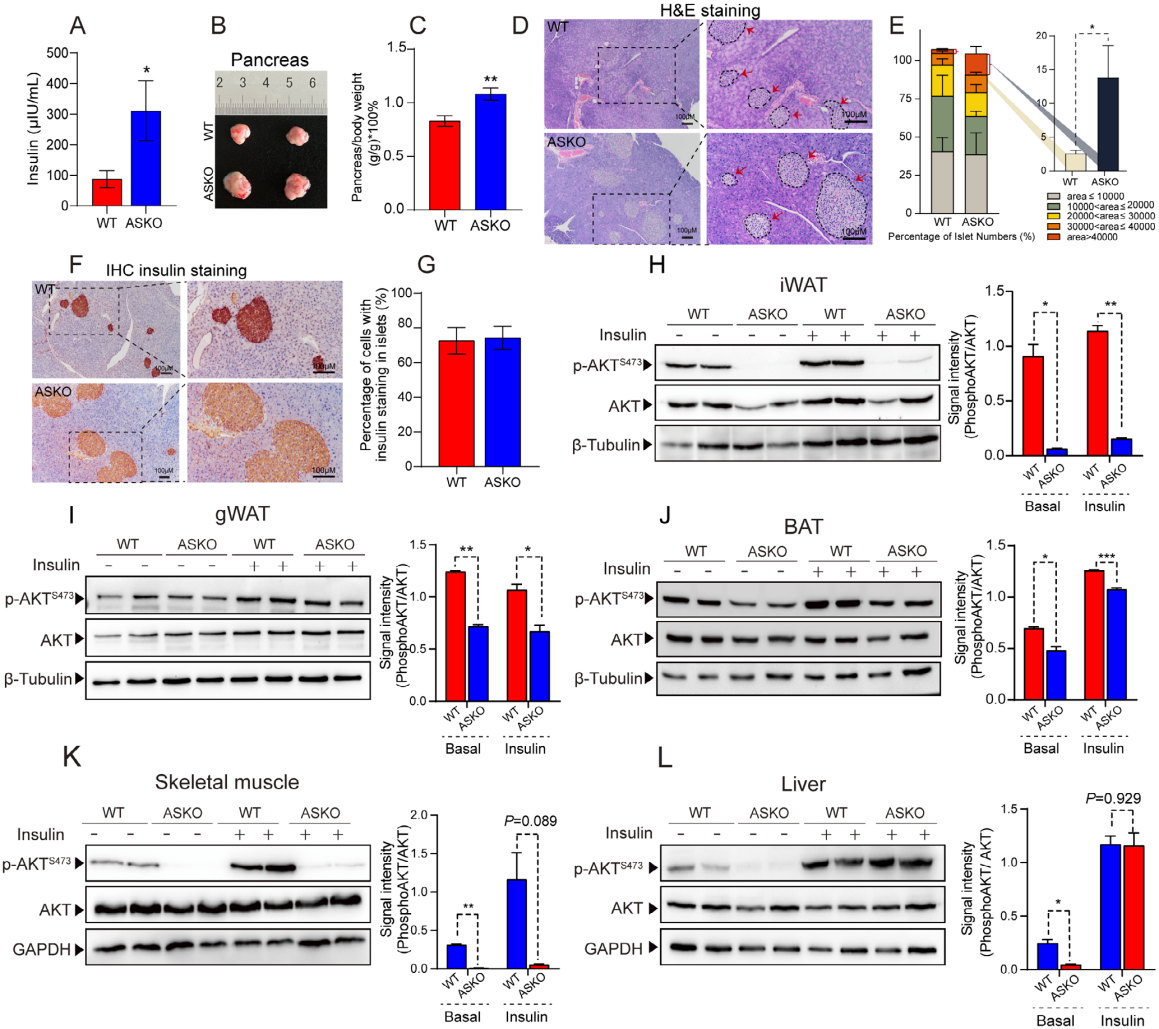
ASKO mice exhibited hyperinsulinaemia and larger islet area. (A) Plasma insulin levels in 6‐month‐old WT and ASKO mice (*n* = 12). (B) Respective image of pancreas tissues from 2‐month‐old WT and ASKO mice. (C) The relative pancreas weight (normalised to body weight) of 6‐month‐old two groups of mice (*n* = 7). (D) Histology images of the pancreas in 6‐month‐old two groups of mice. (E) Statistical analysis of islet area by H&E staining. (F) Insulin immunohistochemical staining of pancreas tissues from two groups of mice. (G) Quantification of the percentage of cells with insulin staining in islets from (F). (H–L) Insulin signalling in iWAT, gWAT, BAT, skeletal muscle and liver of both mice under both basal and insulin stimulated conditions. Mice were injected with 0.75 IU insulin kg^−1^ body weight and the insulin targeted tissues were collected after 10 min. Left: Representative western blotting of p‐AKT^S473^, total AKT. Right: Quantification of p‐AKT^S473^/total AKT from two independent experiments. Data are presented as mean ± SEM. *p < 0.05, **p < 0.01, ***p < 0.001.

We generated the adipocyte‐specific *Rnf20* knockout (ASKO) mice in our previous study and found that these mice suffered hyperinsulinaemia [18]. To further investigate the effect of adipocyte *Rnf20* on insulin signalling, more ASKO and WT littermates were bred, and consistently, the elevated circulating level of insulin was confirmed in 6‐month‐old ASKO mice after fasting (Figure 1A), suggesting that the deletion of the adipocyte *Rnf20* gene might affect the function of the pancreas. Pancreas tissues were isolated from both groups of mice at 2‐month of age, and ASKO mice displayed bigger pancreas compared to control mice (Figure 1B). Because ASKO mice develop progressive fat loss (Figure S1), we weighed the pancreas of both mice at 6 months and found that the relative weight of the pancreas in ASKO mice was significantly higher than that of WT mice (Figure 1C). Histological analysis revealed the enlarged islets in ASKO mice compared to those in controls (Figure 1D); meanwhile, the number of islets was not significantly changed in the two mice (Figure S2). The quantified data showed that the proportion of larger islets (> 40,000 μm^2^) in ASKO mice was three times more than that in WT mice (Figure 1E). Insulin immunohistochemical staining of the pancreas demonstrated that the number of insulin‐positive cells was significantly increased in the islets of ASKO mice (Figure 1F); however, the percentage of these positive cells was not changed in the two mice (Figure 1G). Together, these data suggested that the elevated serum insulin level in ASKO mice might be due to the increased number of β‐cells and enlarged islets.

Both mice and human studies showed that insufficient activation of the PI3K‐AKT pathway is a hallmark of IR and can lead to T2DM [21]. We asked whether the *Rnf20* deletion impaired insulin signalling by performing the level of AKT. The insulin‐targeted tissues, including adipose tissues, skeletal muscle and liver, under both basal and insulin‐induced conditions from both mice were collected for western blotting. Our data revealed a clear reduction of p‐AKT^S473^ in various adipose depots under both conditions (Figure 1H–J, left panel); especially under insulin‐induced conditions, we observed a 92.8% reduction of p‐AKT^S473^ signalling in iWAT (Figure 1H, right panel), a 42.6% reduction in gWAT (Figure 1I, right panel) and a 30.9% reduction in BAT of ASKO mice (Figure 1J, right panel), compared to control mice. In addition, the impaired insulin signalling was not restricted to fat tissues, as the significantly decreased level of p‐AKT^S473^ was also observed in the skeletal muscle of ASKO mice under both basal and insulin‐stimulated conditions (Figure 1K). Despite the level of p‐AKT^S473^ being lower in the liver of ASKO mice under the basal condition, there was no significant alteration under the insulin‐stimulated condition (Figure 1L), suggesting normal hepatic insulin sensitivity in ASKO mice.

### 3.2 Adipocyte *Rnf20* Ablation Suppressed Insulin Signalling in Fat Tissues

**FIGURE 2:**
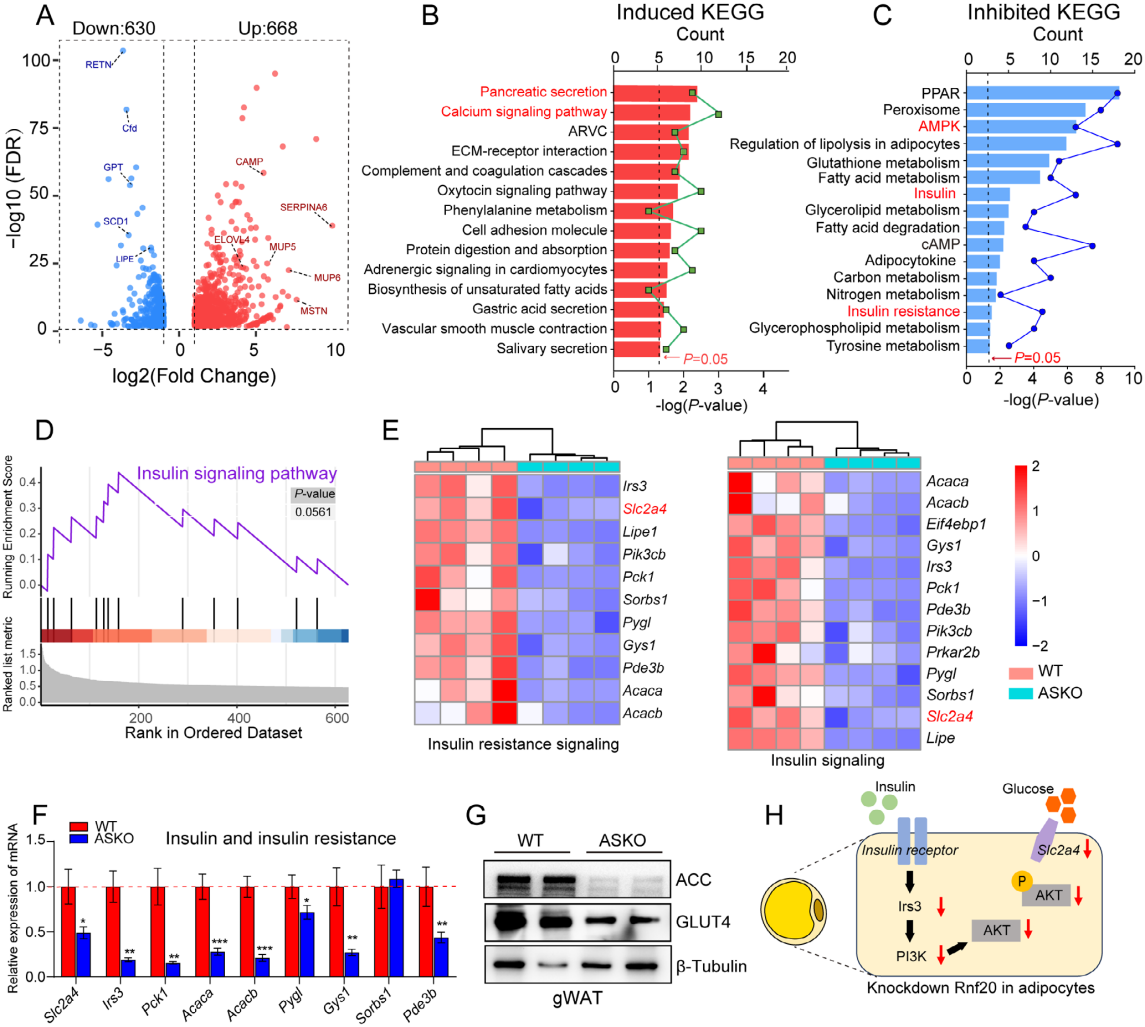
Adipocyte Rnf20 ablation suppressed insulin signalling related genes of fat tissues. (A) Volcano plot showed FC versus *p*‐value for RNA‐Seq data generated from gWAT of 6‐month‐old ASKO mice compared with littermate controls. The significantly upregulated genes are represented by red dots, whereas downregulated genes are represented by blue dots; *n* = 5 mice each. (B–C) KEGG pathway analysis of the upregulated (B) and downregulated genes (C) in gWAT of both mice. (D) GASE analysis of downregulated DEGs in gWAT. (E) Heatmap of genes involved in insulin resistance and insulin signalling. (F) The mRNA expression levels of genes involved in insulin and insulin resistance signalling pathways. (G) Western blot showed the levels of protein ACC and GLUT4 in gWAT of both mice. (H) Schematic diagram of RNF20 knockdown inhibited insulin and glucose transport signallings. Data are presented as mean ± SEM. *p < 0.05, **p < 0.01, ***p < 0.001.

It is commonly recognised that visceral adipose tissues are associated with insulin resistance [22]. To study the detailed molecular mechanisms by which *Rnf20* regulates insulin sensitivity in adipose tissue, genome‐wide RNA sequencing (RNA‐seq) was performed on gWAT of 6‐month‐old WT and ASKO mice (16‐h‐fasted). The principal component analysis (PCA) plot showed a distinguished cluster of gWAT from two groups of mice (Figure S3). A global overview of the changes in gene expression was shown in volcano plots (Figure 2A). Using a criterion of *p*‐value < 0.05 and fold change (FC) > 2, we identified 1298 differentially expressed genes (DEGs), of which 668 were significantly upregulated and 630 were significantly downregulated (Figure 2A). Kyoto Encyclopedia of Genes and Genomes (KEGG) analysis showed that the upregulated DEGs were enriched in pancreatic secretion and calcium signalling pathways (Figure 2B), while the downregulated DEGs were enriched in insulin sensitivity‐related pathways, including AMPK, cAMP, and insulin resistance signalling pathways (Figure 2C). Consistently, gene set enrichment analysis (GSEA) of RNA‐seq data showed that the downregulated DEGs were highly enriched in the insulin signalling pathway (Figure 2D). For better visualisation, heatmaps were built to show that the levels of insulin action‐related genes (*Irs3*, *Slc2a4*, *Pck1*, *Pygl*, *Gysl*, *Sorbs1*, *Pde3b*, *Pik3cb*, *Acaca* and *Acacb*) were markedly decreased by adipose‐specific *Rnf20* ablation (Figure 2E), and the expression levels of these genes were further confirmed by qPCR (Figure 2F). In addition, a significant reduction of these insulin‐related genes was also observed in iWAT and BAT in 6‐month‐old ASKO mice compared to control mice (Figure S4). Besides that, we also observed the down‐regulation of cAMP signalling pathway‐related genes in gWAT of ASKO mice compared to WT littermates (Figure S5), which was also an important pathway for maintaining insulin homoeostasis [23]. Note that two critical genes that are involved in insulin signalling, including the insulin‐sensitive glucose transporter gene, *Slc2a4*, and the rate‐limiting enzyme in fatty acid metabolism, *Acaca* gene, were observed to be downregulated. The proteins that both genes encoded, GLUT4 and ACC, were also decreased in gWAT of ASKO mice (Figure 2G). It is well known that the adipose GLUT4 suppression results in systemic insulin resistance [24, 25]. We speculate that the insulin signalling impairment in ASKO mice is probably via decreasing the levels of GLUT4 (Figure 2H).

### 3.3 *Rnf20* Deficiency Decreased the Level of H3K4me3 in Adipocytes

**FIGURE 3:**
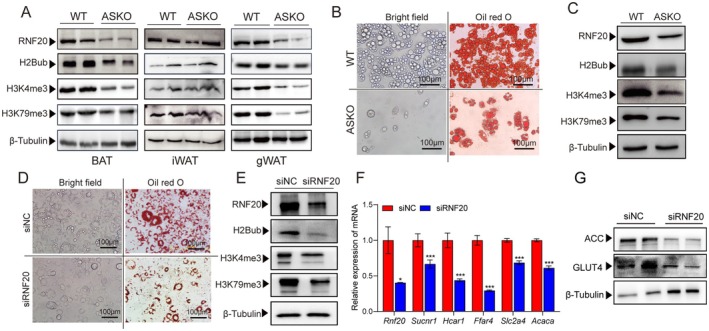
Adipose‐specific deletion of *Rnf20* inhibited the level of histone modifications in adipose tissues. (A) The expression level of proteins, including RNF20 and histone modifications, in the BAT, iWAT, and gWAT from 6‐month‐old WT and ASKO mice. (B) The differentiation efficiency was evaluated by lipid droplet formation under a brightfield microscope and Oil red O staining in primary preadipocytes from WT and ASKO mice. (C) The expression levels of RNF20 and histone modifications in primary adipocytes from two mice. (D) The differentiation efficiency was dramatically decreased in the siRNF20‐treated 3T3‐L1 cells based on lipid droplet formation under a brightfield microscopy and Oil red O staining. (E) The expression level of RNF20 and histone modifications (H2Bub, H3K4me3, H3K79me3) in siNC‐ and siRNF20‐treated cells. (F) The expression levels of *Rnf20* and insulin signalling related genes in siNC and siRNF20 cells. (G) The levels of protein GLUT4 and ACC in siNC and siRNF20 cells. Data are presented as mean ± SEM. *p < 0.05, **p < 0.01, ***p < 0.001.

RNF20 and its mediated H2Bub on promoting gene transcription via cross‐talking with other active histone markers, such as H3K4me3 and H3K79me3, have been reported in various tissues of mammals [26] and histone modifications are involved in the regulation of GLUT4 expression [8, 27, 28]. As expected, the RNF20‐related histone modifications were found to be suppressed in various fat depots of ASKO mice (Figure 3A). Then, we cultured white preadipocytes from 6‐week‐old ASKO and WT mice and induced to differentiate for 8 days. We found that the adipogenic differentiation efficiency was greatly inhibited in preadipocytes from ASKO mice under both brightfield microscopy and ORO staining (Figure 3B), accompanied by the suppressed levels of RNF20, H2Bub, H3K4me3 and H3K79me3 (Figure 3C). Besides that, we also used siRNA to knock down the expression level of the *Rnf20* gene in 3 T3‐L1 cells and then induce differentiation. The strikingly few lipid droplets observed in siRNF20‐transfected cells under both brightfield microscopy (Figure 3D, bottom left) and ORO staining (Figure 3D, bottom right) were consistent with the previous mice studies; adipocyte‐specific *Rnf20* ablation resulted in the loss of weight of fat depots [18]. We found that the levels of RNF20 and histone modifications were also significantly decreased in siRNF20‐transfected cells compared to siNC cells (Figure 3E). Consistent with RNA‐Seq data, the expression levels of insulin signalling‐related genes (*Rnf20*, *Sucnr1*, *Hcar1*, *Ffar4*, *Slc2a4*, and *Acaca*) were also highly reduced in siRNF20‐treated cells (Figure 3F). Consistent with in vivo studies, *Rnf20* knockdown suppressed the protein levels of GLUT4 and ACC (Figure 3G). In vivo and in vitro studies collectively illustrate that RNF20 is critical for maintaining the levels of H2Bub, H3K4me3 and H3K79me3.

### 3.4 Knockdown of *Rnf20* Decreased the H3K4me3 Occupancy of *Slc2a4* in 3T3‐L1 Cells

**FIGURE 4:**
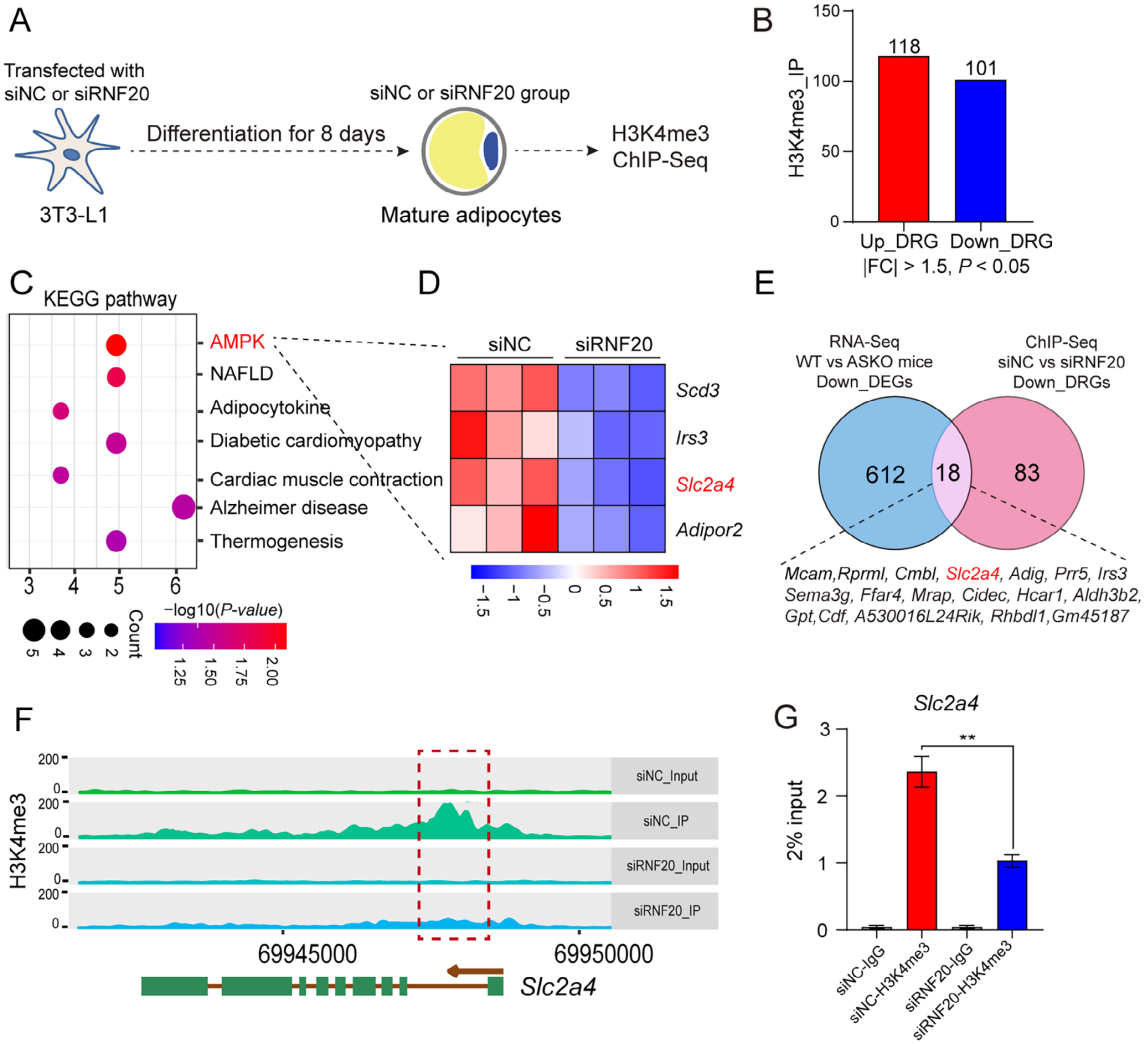
Knockdown of RNF20 decreased the H3K4me3 occupancy of *Slc2a4*. (A) Schematic diagram of transfection of siRNAs in 3T3 cells. (B) The number of Up_DRGs and Down_URGs. (C) Enrichment analysis of the Down_DRGs related genes. (D) Heatmap showing the Down_DRGs related genes in AMPK signalling. (E) Venn diagram indicating the overlap of genes with downregulated expression levels in gWAT of ASKO mice (left, blue) and reduced H3K4me3 modification levels (right, pink) in RNF20‐knockdown cells. (F) Snapshot of H3K4me3 modification near the TSS of the *Slc2a4* gene in siNC and siRNF20 mature adipocytes. (G) Result of ChIP‐qPCR to confirm changes in H3K4me3 modification near the TSS of the *Slc2a4* gene in siNC and siRNF20 cells. Data are presented as mean ± SEM. *p < 0.05, **p < 0.01.

Recent evidence demonstrated a negative correlation between the level of H3K4me3 and insulin resistance in subcutaneous adipose tissues from insulin resistance patients [29]. To further explore the molecular mechanisms of *Rnf20* knockdown that caused the suppression of GLUT4, we performed chromatin immunoprecipitation with sequencing (ChIP‐Seq) analysis of H3K4me3, an active histone marker, with siNC‐ and siRNF20‐transfected cells (Figure 4A). ChIP‐Seq profiles identified 219 differentially enriched region‐related genes (DRGs) in both cells, including a total of 101 genes with lower H3K4me3 enrichment (Down_DRGs), while 118 genes had higher enrichment (Up_DRGs) upon the deletion of the *Rnf20* gene (cut off: *p* < 0.05, FC > 1.5) (Figure 4B, Table S3). Considering the negative relationship between the level of insulin resistance and the enrichment of H3K4me3 [29], we paid more attention to the targeted genes with the reduced H3K4me3 level. We found that the Down_DRGs were mainly enriched in pathways related to fat metabolism, including AMPK, non‐alcoholic fatty acid liver (NAFLD), and adipocytokine (Figure 4C). Mice studies showed that the NAFLD and AMPK signalling pathways were associated with insulin resistance [30, 31]. In addition, heatmap analysis showed that the H3K4me3 peaks of *Scd3*, *Irs3*, *Slc2a4* and *Adipor2* genes were significantly suppressed in the siRNF20 group (Figure 4D). To further correlate chromatin bindings with direct gene regulation, we integrated the RNA‐Seq and ChIP‐Seq data and noticed that a total of 18 downregulated expressed genes were associated with the decreased H3K4me3 levels due to *Rnf20* gene ablation (Figure 4E), suggesting that these genes might be directly regulated by the RNF20‐H2Bub‐H3K4me3 axis. Notably, the H3K4me3 enrichment of the *Slc2a4* gene was significantly decreased in siRNF20‐treated cells compared to those from control cells (Figure 4F). This observation was further confirmed by using ChIP‐qPCR (Figure 4G). Together, these data indicated the expression level of the *Slc2a4* gene was regulated by the RNF20‐H2Bub‐H3K4me3 axis in adipocytes.

## 4 Discussion

**FIGURE 5:**
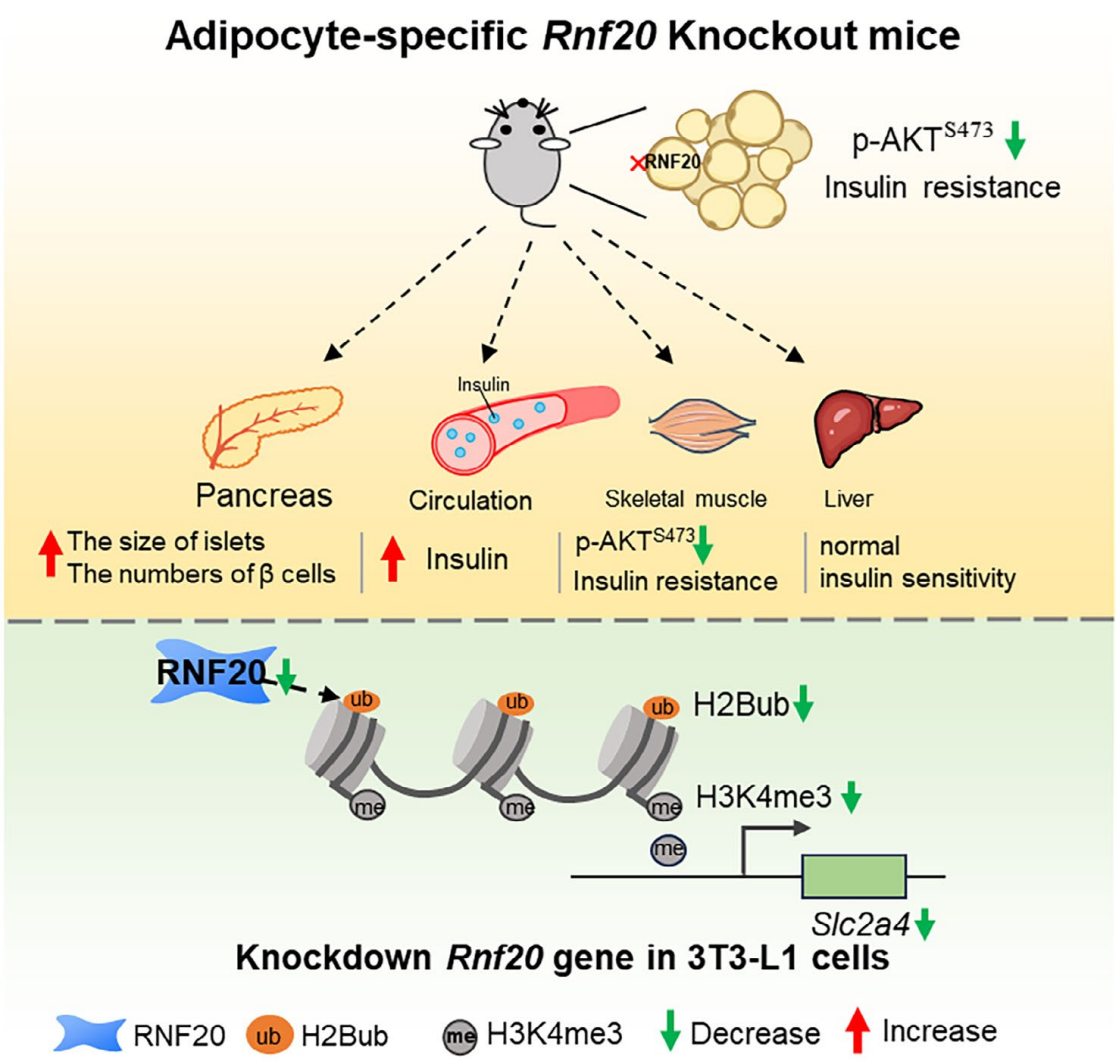
Schematic diagram of the effect of *Rnf20* on glucose systemic metabolism.

Our study reveals a novel role of RNF20 in maintaining systemic insulin homoeostasis by regulating the expression of the *Slc2a4* gene. Specifically, we observed that complete ablation of the *Rnf20* gene in adipocytes, which encodes the E3 ligases responsible for H2B monoubiquitination, led to hyperinsulinaemia and adipose tissue‐specific insulin resistance in mice. Further molecular analysis demonstrated that the loss of RNF20 reduced H3K4me3 modifications at the *Slc2a4* gene locus, thereby decreasing its expression and ultimately contributing to the development of insulin resistance (Figure 5). These findings highlight the importance of adipocyte RNF20 in maintaining glucose metabolism and provide new insights into the epigenetic regulation of insulin signalling.

Here, we found that ASKO mice exhibited elevated circulating insulin levels, accompanied by an increase in islet sizes and beta‐cell numbers, making them a good animal model for investigating the pathogenesis of hyperinsulinaemia. Numerous genetically modified mouse models have demonstrated that defective insulin signalling can lead to hyperinsulinaemia. Examples include the disruption of leptin signalling [32], the lack of *Akt* gene [33], the heterozygous mutation of insulin receptor (*Insr*) [34], the deletion of *Bcl2*‐associated athanogene 3 (*Bag3*) [35], the deficiency of a protein kinase D2 (*Prkd2*) [36], the inhibition of IGF‐receptor(*Igfr*) [37] and the heterozygous mutation of *Seipin* in beta‐cell of islet [38]. To further understand this mechanism, it is crucial to collect more clinical data to clarify the correlation between RNF20 expression levels in adipose tissue and plasma insulin concentration in both healthy individuals and patients with T2DM.

Insulin resistance is a hallmark of hyperinsulinaemia, characterised by a diminished response of insulin‐sensitive tissues (such as adipose tissue, skeletal muscle and liver) to insulin stimulation [2, 39]. Our previous ITT results demonstrated that ASKO mice exhibited mild insulin resistance at 6 months of age [18]. Insulin exerts its effects by activating the AKT signalling pathway, which involves key molecules such as the INRS, IRS, PI3K and AKT [40]. Among these, phosphorylation of AKT at the Ser473 site is widely recognised as a critical biomarker of insulin resistance [41]. Clinical studies have further shown that both systemic and peripheral insulin resistance are strongly associated with reduced AKT activation in adipose tissues [42]. Herein, our study demonstrated that insulin resistance occurs in both adipose tissue and skeletal muscle of ASKO mice, while hepatic insulin sensitivity under insulin stimulation remains unaffected. Further research is needed to determine whether RNF20 indirectly regulates systemic insulin homoeostasis through inter‐organ signalling mechanisms, providing potential new therapeutic targets for treating insulin resistance‐related metabolic disorders.

We revealed the essential role of RNF20 in GLUT4 expression in adipose tissues, as evidenced by the significantly reduced expression of the *Slc2a4* gene and its encoded GLUT4 protein in cell and mice studies. GLUT4 is a key transporter protein predominantly expressed in skeletal muscle and adipose tissue, and its high expression is critical for maintaining insulin sensitivity in these tissues [43]. As early as 1995, studies on GLUT4‐deficient mice (both male and female) revealed postprandial hyperinsulinaemia, highlighting the necessity of GLUT4 for maintaining normal insulin levels [44]. Subsequent research confirmed that GLUT4^+/−^ heterozygous mice exhibited systemic insulin resistance due to reduced GLUT4 levels in adipose tissue and muscle [45]. Muscle‐specific GLUT4 knockout mice developed severe insulin resistance and glucose intolerance [46], while adipose‐specific GLUT4 knockout mice showed impaired insulin responses in both the liver and muscle [47]. Despite that adipocyte RNF20 is essential for GLUT 4 expression, further research is needed to fully elucidate its function in glucose transport rate, glucose phosphorylation activity and insulin‐stimulated GLUT4 translocation.

Initially discovered in yeast, H2B monoubiquitination regulates H3K4me3, an epigenetic mark associated with increased transcription elongation rates [48]. In intestinal inflammation of inflammatory bowel disease (IBD) patients and mouse models, the loss of H2Bub1 following intestinal *Rnf20* or *Rnf40* deletion resulted in decreased H3K4me3 occupancy in the transcribed region of various IBD‐associated genes [16]. In β cell islets, the deletion of *Rnf20* or *Rnf40* reduced H2Bub marks and uncovered several target genes, including *Glut2*, *Ucp2* and MAF BZIP transcription factor A (MafA) [49]. Consistently, we observed that the loss of H2Bub following adipocyte *Rnf20* deletion resulted in reduced H3K4me3 occupancy across the transcribed regions of several AMPK‐related genes (Figure 4C,D), which is a critical pathway for energy metabolism. Notably, the loss of H3K4me4 enrichment occurred in the *Slc2a4* gene locus in *Rnf20* knockdown adipocytes, as well as the decrease in *Slc2a4* mRNA and protein amounts, indicating that the RNF20‐H2Bub‐H3K4me3 axis could directly regulate the expression level of the *Slc2a4* gene. However, whether overexpression of the *Slc2a4* gene in RNF20 knockdown cells could alleviate insulin resistance remains to be investigated, which would better elucidate the role of RNF20 in insulin signalling via *Slc2a4* regulation.

Furthermore, RNF20's function likely extends far beyond regulating gene expression through H2B ubiquitination. Our RNA‐Seq data revealed that the loss of *Rnf20* in adipose tissue triggers widespread differential gene expression, further suggesting that RNF20 may regulate gene expression through other, yet unidentified molecular pathways. Thus, future research should integrate mass spectrometry and protein interaction network analysis to systematically identify RNF20‐dependent protein networks. This comprehensive approach will not only enhance our understanding of RNF20's multidimensional roles in gene regulation and cellular function but also provide new theoretical foundations and research directions for elucidating the molecular mechanisms and developing intervention strategies for metabolic diseases.

## 5 Conclusion

In conclusion, this study uncovered a previously unrecognised role of RNF20 and its mediated H2Bub in establishing broad H3K4me3 domains, which facilitate the transcriptional elongation of the *Slc2a4* gene. These findings not only deepen our understanding of the biological functions of *Rnf20* in maintaining systemic metabolic homoeostasis but also highlight RNF20 as a potential therapeutic target for the treatment of IR and T2DM.

## Author Contributions

**Ying Zhao:** data curation (lead), investigation (equal), methodology (equal) and writing‐original draft (lead). **Xiaojuan Liang:** data curation (equal) and methodology (equal). **Jiayu Tang:** investigation (equal) and data curation (equal). **Chunwei Cao:** data curation (equal) and formal analysis (equal). **Chunhuai Yang:** formal analysis (equal) and methodology (equal). **Shulin Yang:** conceptualisation (supporting) and supervision (equal). **Jianguo Zhao:** funding acquisition (equal), project administration (equal) and resources (equal). **Jinxiang Yuan:** funding acquisition (equal), supervision (equal), writing – review and editing (equal). **Meng Zhang:** funding acquisition (equal), conceptualisation (equal), writing – review and editing (equal). **Yanfang Wang:** conceptualisation (lead), writing – review and editing (lead), funding acquisition (lead), project administration (equal), resources (equal) and supervision (equal).

## Conflicts of Interest

The authors declare no conflicts of interest.

## Supporting information


Appendix S1

**Table S1** The sequence information of qPCR and ChIP‐qPCR.

**Table S2** RNA seq analysis of gWAT in both mice.

**Table S3** ChIP seq analysis of DRGs in siNC and siRNF20 cells.

## Data Availability

The datasets generated during and/or analysed during the current study are not publicly available but are available from the corresponding author on reasonable request.
