# Supplementary material for: Adipocyte RNF20 Knockout Leads to Hyperinsulinemia via the H2Bub‐H3K4me3‐Slc2a4 Axis
Source: J Cell Mol Med. 2025 Jun 16;29(11):e70649. doi: 10.1111/jcmm.70649 (PMC12168218; doi:10.1111/jcmm.70649)
Supplement: Supplementary file 1 — Appendix S1 [file JCMM-29-e70649-s004.pdf]

Supplementary figures and figure legends

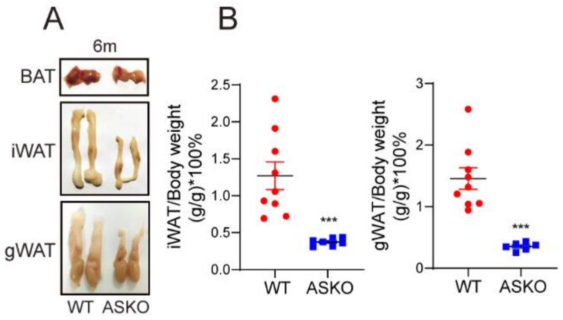

Figure S1 The fat tissues were significantly decreased in ASKO mice. (A) The respective image of fat tissues in 6-month-old WT and ASKO mice. (B) The relative weight of fat tissues in two groups of mice. Data are presented as mean ± SEM.

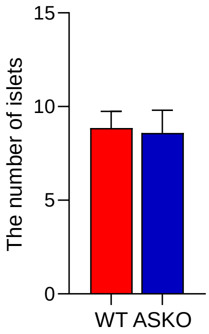

Figure S2 The number of islets in pancreas tissues from WT and ASKO mice.

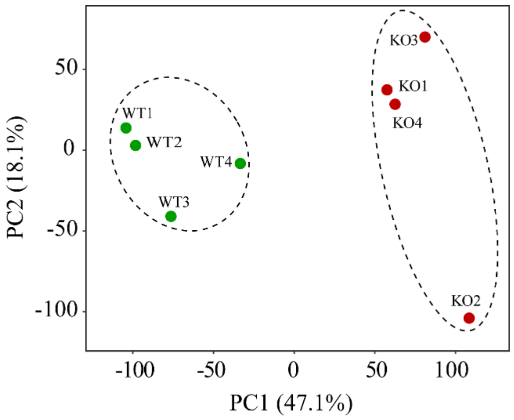

Figure S3 Principal component analysis (PCA) plots representing the different genotypes. Green dots represented as WT mice; red dots represented as ASKO mice.

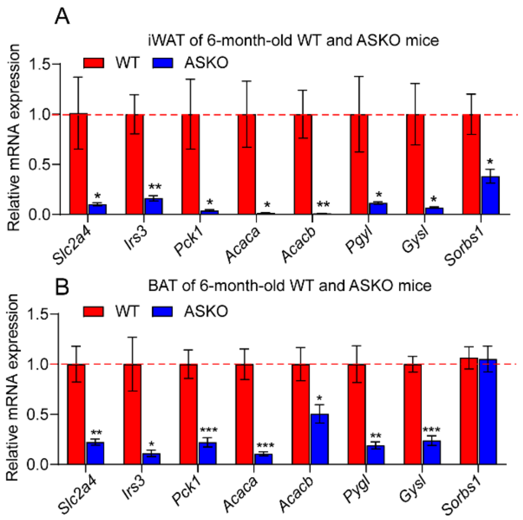

Figure S4 Deletion of adipocyte *Rnf20* gene significantly inhibited insulin resistance-related genes. (A) Expression levels of insulin resistance-related genes were detected by qPCR in iWAT of 6-month-old WT and ASKO mice. (B) Expression levels of insulin resistance-related genes were detected by qPCR in BAT of 6-month-old WT and ASKO mice. *Data are presented as mean ± SEM.* * $P < 0.05$, ** $P < 0.01$, *** $P < 0.001$.

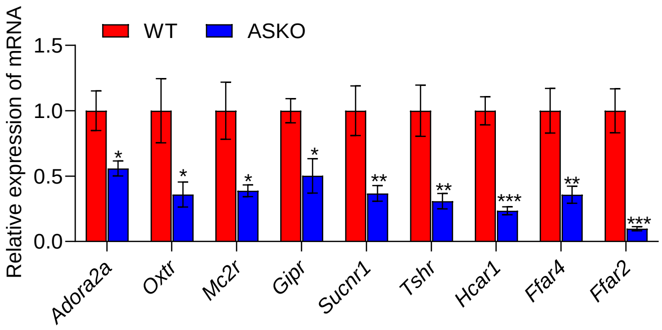

Figure S5 The expression levels of cAMP-related genes were significantly inhibited in gWAT of ASKO mice.
