# Supplementary material for: Adipocyte RNF20 Knockout Leads to Hyperinsulinemia via the H2Bub‐H3K4me3‐Slc2a4 Axis
Source: J Cell Mol Med. 2025 Jun 16;29(11):e70649. doi: 10.1111/jcmm.70649 (PMC12168218; doi:10.1111/jcmm.70649)
Supplement: Supplementary file 2 — Table S1 The sequence information of qPCR and ChIP‐qPCR. [file JCMM-29-e70649-s001.pdf]

Table S1 The sequence information of qPCR and ChIP-qPCR primers

Gene name	sequence
18s	F: GTAACCCGTTGAACCCCATT<br>R: CCATCCAATCGGTAGTAGCG
Irs3	F: CTCGGCTCACCGTTTCCTT<br>R: CGCGCTTGCTAATAGTGCAG
Slc4a2	F: GTGACTGGAACACTGGTCCTA<br>R: CCAGCCACGTTGCATTGTAG
Pck1	F: CTGCATAACGGTCTGGACTTC<br>R: GCCTTCCACGAACTTCCTCAC
Pygl	F: GAGAAGCGACGGCAGATCAG<br>R: CTTGACCAGAGTGAAGTGCAG
Gysl	F: GAACGCAGTGCTTTTCGAGG<br>R: GCTCCGTGTATGGTCCCAC
Sorsb1	F: AGACATGGACCCTACCAAAATCT<br>R: GACTTGCTTTCATGCTTCGGA
Pde3b	F: AAAGCGCAGCCGGTTACTAT<br>R: CCATATTGCGAGCTTCATTTAGC
Acaca	F: CTCCCGATTCATAATTGGGTCTG<br>R: TCGACCTTGTTTTACTAGGTGC
Acacb	F: CGCTCACCAACAGTAAGGTGG<br>R: GCTTGGCAGGGAGTTCCTC
Rnf20	F: AGCAGAAATGCTAGATCAGCG<br>R: GCGGATGTTTTCATCAAACTGG
Sucnr1	F: TCTTGTGAGAATTGGTTGGCAA<br>R: CATCTCCATAGGTCCCCTTATCA
Hcar1	F: CGGGTCGTGCTGTCTCATC<br>R: TCCGAAGGGGTAAGCAGATCA
Ffar4	F: CTTGATCCTCACGGCCTACAT<br>R: CCAGGGTCAGATTAAGCAGGAG
Ffar2	F: CTTGATCCTCACGGCCTACAT<br>R: CCAGGGTCAGATTAAGCAGGAG
Adora2a2	F: GCCATCCCATTCGCCATCA<br>R: GCAATAGCCAAGAGGCTGAAGA
Oxtr	F: GGCCGTGTTCCAGGTTCTC<br>R: TGCAAGTATTTGACCAGACGAC
Mc2r	F: AAGCCTCGTGGCAGTTTTGAA<br>R: TCACAATGCTATGGTATTGCAGG
Gipr	F: CCACTGGGTCCCTACACTG<br>R: TCTGAGCGTCCCACGATCA
Tshr	F: CACCAGGAGGACGACTTCAGA<br>R: GGCAGACTCGAAAATGCAAGA

Slc2a4 ChIP-qPCR	F: CAAGCGGGTCTCACTAGATC<br>R: AGACTCAGGCGCTGCAATAA
