# Supplementary material for: Adipocyte RNF20 Knockout Leads to Hyperinsulinemia via the H2Bub‐H3K4me3‐Slc2a4 Axis
Source: J Cell Mol Med. 2025 Jun 16;29(11):e70649. doi: 10.1111/jcmm.70649 (PMC12168218; doi:10.1111/jcmm.70649)
Supplement: Supplementary file 3 — Table S2 RNA seq analysis of gWAT in both mice. [file JCMM-29-e70649-s003.pdf]

ID	symbol name	WT_1	WT_2	WT_3	WT_4	ASKO_1	ASKO_	ASKO_3	ASKO_4	logFC	PValue	FDR
Up_DEGs												
ENSMUSG00000085218	BB218582	1	2	6	6	121	177	103	119	4.78979262	2.17E-21	2.28E-18
ENSMUSG00000087651	1500009L16Rik	26	29	55	60	127	125	154	161	1.47249047	7.37E-05	0.0029539
ENSMUSG00000030030	1700003E16Rik	29	10	15	94	118	112	203	164	1.77063166	0.0023132	0.0446753
ENSMUSG00000031847	1700030J22Rik	13	15	13	59	75	65	117	114	1.64588177	0.0017386	0.0360494
ENSMUSG00000108207	1810059H22Rik	60	34	25	99	165	151	193	215	1.46065171	0.0007272	0.0185907
ENSMUSG00000097354	2310001H17Rik	138	117	107	218	358	572	422	434	1.33271238	2.53E-05	0.0012233
ENSMUSG00000070282	3000002C10 Rik	31	32	27	37	142	167	218	155	2.13025465	7.68E-10	1.28E-07
ENSMUSG00000038917	3930402G23Rik	12	6	24	6	71	410	59	79	3.33080018	1.64E-06	0.0001137
ENSMUSG00000109089	4833411C07Rik	14	24	17	4	30	305	67	83	2.6575305	0.0001208	0.0044433
ENSMUSG00000108187	4930511E03Rik	56	39	35	33	133	98	126	248	1.59955875	0.0001673	0.0058045
ENSMUSG00000102555	6430511E19Rik	54	59	50	80	154	156	141	168	1.06457226	0.0008552	0.0210832
ENSMUSG00000105095	8430422M14Rik	105	73	56	107	483	281	382	268	1.76666367	1.40E-06	9.93E-05
ENSMUSG00000114452	A530001N23Rik	173	44	9	69	447	348	258	365	1.93862717	0.0016862	0.0352407
ENSMUSG00000083307	AA414768	37	29	63	63	123	135	192	107	1.26219751	0.0007974	0.0199701
ENSMUSG00000068522	Aard	59	24	25	6	191	264	502	237	3.04968282	1.60E-07	1.49E-05
ENSMUSG00000029695	Aass	36	36	35	172	207	160	378	260	1.61516106	0.0022194	0.0433472
ENSMUSG00000072949	Acot1	47	69	52	344	473	467	635	582	1.84512873	0.0006305	0.0165496
ENSMUSG00000034853	Acot11	81	87	122	244	509	300	592	510	1.59456842	5.30E-05	0.0022583
ENSMUSG00000021751	Acox2	7	2	3	38	225	192	162	268	3.84210958	2.30E-08	2.63E-06
ENSMUSG00000031278	Acsl4	838	1143	1230	929	3062	3219	1648	2856	1.08832963	2.18E-05	0.0010836
ENSMUSG00000059430	Actg2	41	80	729	309	6515	878	958	768	2.77529359	0.0004733	0.0132176
ENSMUSG00000056367	Actr3b	92	92	133	144	290	274	308	263	1.02374063	0.0005474	0.0147954
ENSMUSG00000020926	Adam11	148	145	142	819	1798	1833	2493	1700	2.40684648	1.15E-06	8.35E-05
ENSMUSG00000033453	Adamts15	1409	1320	713	1997	7182	3496	10129	6480	2.0555887	8.11E-10	1.34E-07
ENSMUSG00000031994	Adamts8	7	18	62	38	232	111	130	122	2.00871064	0.0002307	0.0075355
ENSMUSG00000036040	Adamtsl2	22	25	173	57	6655	1221	11890	13466	6.68362646	3.44E-18	2.27E-15
ENSMUSG00000021536	Adcy2	15	19	21	58	85	83	101	78	1.35858314	0.0021334	0.0420368
ENSMUSG00000050994	Adgb	73	40	61	214	538	485	734	597	2.35393834	2.33E-07	2.04E-05
ENSMUSG00000028782	Adgrb2	92	99	105	102	280	264	351	296	1.29449037	1.03E-05	0.0005696
ENSMUSG00000040812	Agbl2	20	22	15	108	167	110	208	171	1.75882364	0.0017973	0.0370032
ENSMUSG00000021575	Ahrr	14	35	17	31	67	165	60	70	1.58013328	0.0007267	0.0185907
ENSMUSG00000022868	Ahsg	16	11	25	16	88	223	82	84	2.48946227	7.75E-08	7.84E-06

ENSMUSG00000022763	Aifm3	53	79	39	131	305	237	450	247	1.76659512	2.28E-05	0.0011211
ENSMUSG00000028527	Ak4	32	32	70	77	139	109	267	167	1.43716826	0.0007691	0.0194238
ENSMUSG00000052131	Akr1b7	1	128	11	17	64714	34	3	17	8.41032039	0.0001868	0.0063232
ENSMUSG00000029368	Alb	233	107	264	103	678	563	898	1069	1.89463375	1.61E-06	0.0001121
ENSMUSG00000020256	Aldh1l2	7	22	26	28	93	121	82	74	1.87003578	1.28E-05	0.00069
ENSMUSG00000019102	Aldh3a1	2	3	5	3	72	124	167	80	4.74983685	2.51E-18	1.71E-15
ENSMUSG00000028737	Aldh4a1	952	893	631	791	2830	1550	1928	2508	1.14463127	5.46E-05	0.0023122
ENSMUSG00000038763	Alpk3	21	39	18	28	122	107	159	151	2.05210008	2.31E-07	2.03E-05
ENSMUSG00000040310	Alx4	79	62	59	88	282	238	343	295	1.72285023	8.62E-08	8.56E-06
ENSMUSG00000075232	Amd1	1060	1023	1156	1615	3619	2905	3658	2931	1.15944987	9.54E-08	9.37E-06
ENSMUSG00000027460	Angpt4	30	220	239	516	6060	1653	8492	9004	4.43325108	4.11E-12	1.01E-09
ENSMUSG00000028989	Angptl7	6	47	105	18	322	198	169	120	1.93182101	0.0026204	0.0489721
ENSMUSG00000031543	Ank1	59	66	84	97	1260	133	101	125	2.16566278	0.0022032	0.0430596
ENSMUSG00000069601	Ank3	44	63	105	628	1169	682	1088	844	1.96769301	0.0017215	0.0357749
ENSMUSG00000055114	Anxa13	3	3	0	4	860	2	19	4	6.16588688	8.66E-05	0.0033848
ENSMUSG00000021950	Anxa8	353	401	248	588	1472	1349	540	1639	1.37358262	0.0002147	0.007101
ENSMUSG00000062444	Ap3b2	33	27	60	90	179	208	455	204	2.0579752	5.99E-06	0.0003552
ENSMUSG00000022548	Apod	611	1238	315	4066	5007	4842	10531	5461	1.81509946	0.0007068	0.0181961
ENSMUSG00000023013	Aqp2	21	7	72	92	552	290	258	323	2.66337812	6.99E-06	0.0004076
ENSMUSG00000032204	Aqp9	84	63	67	226	578	263	461	238	1.56161801	0.0007314	0.0186579
ENSMUSG00000041444	Arhgap32	938	961	773	1161	2085	2543	2485	2880	1.09283487	1.51E-06	0.0001064
ENSMUSG00000036198	Arhgap36	11	9	3	10	302	304	253	460	5.01113312	4.32E-27	8.88E-24
ENSMUSG00000078954	Arhgap8	49	26	34	69	181	164	224	246	1.92116461	6.10E-07	4.81E-05
ENSMUSG00000028919	Arhgef19	25	19	64	337	785	592	1061	1126	2.80162097	4.28E-05	0.0018957
ENSMUSG00000037683	Armc3	2	4	9	53	65	54	169	116	2.35946574	0.0019313	0.0390099
ENSMUSG00000015709	Arnt2	13	6	52	160	189	211	557	282	2.21251677	0.0021935	0.0429097
ENSMUSG00000035277	Arx	421	746	624	620	1761	1491	2547	3089	1.6056584	1.18E-07	1.13E-05
ENSMUSG00000031382	Asb11	10	11	7	39	78	51	255	74	2.52047798	8.57E-05	0.0033575
ENSMUSG00000021200	Asb2	111	69	151	136	454	692	189	237	1.44955133	0.0005973	0.0158414
ENSMUSG00000020704	Asic2	60	44	37	59	150	161	169	192	1.45528066	1.73E-05	0.0008989
ENSMUSG00000076441	Ass1	136	126	287	304	600	728	601	427	1.19502852	0.0004406	0.0124529
ENSMUSG00000028373	Astn2	117	122	69	156	252	321	431	302	1.20014029	0.0004924	0.0136392
ENSMUSG00000031376	Atp2b3	2	9	9	11	158	72	561	233	4.77139774	2.92E-12	7.45E-10
ENSMUSG00000020566	Atp6v1c2	29	24	40	72	258	241	160	318	2.30439631	1.99E-08	2.36E-06

ENSMUSG00000006567	Atp7b	8	22	5	41	98	98	115	108	2.19296834	4.36E-05	0.0019205
ENSMUSG00000028457	Atp8b5	38	36	43	79	126	117	167	115	1.15104939	0.0016958	0.0353703
ENSMUSG00000037053	Azgp1	1	2	0	0	165	131	124	82	6.8908528	3.99E-26	6.92E-23
ENSMUSG00000111658	B930092H01 gene	9	13	29	24	159	167	244	150	2.98748008	1.91E-12	5.10E-10
ENSMUSG00000085133	B930095G15Rik	63	55	88	157	259	283	381	330	1.52672101	3.33E-05	0.0015437
ENSMUSG00000044361	BC024139	17	20	26	44	78	113	94	152	1.75748643	1.92E-05	0.0009771
ENSMUSG00000033722	BC034090	187	131	188	399	1036	737	1157	1180	1.92968806	5.25E-08	5.47E-06
ENSMUSG00000061877	BC048679	0	3	0	6	0	30	972	0	6.48348552	0.0012989	0.0288284
ENSMUSG00000057246	BC051142	64	101	71	174	313	386	419	290	1.50365972	3.01E-05	0.001415
ENSMUSG00000000861	Bcl11a	49	30	37	70	136	398	81	155	1.73210961	0.000331	0.0099069
ENSMUSG00000046598	Bdh1	15	15	44	40	332	123	154	126	2.44192796	1.10E-06	8.07E-05
ENSMUSG00000042750	Bex2	4	9	5	120	212	186	377	217	2.64131645	0.0013901	0.0305832
ENSMUSG00000046432	Bex3	135	158	195	491	576	620	1076	681	1.34264095	0.0007583	0.0192139
ENSMUSG00000030103	Bhlhe40	650	531	1605	1171	1676	2316	2702	3474	1.10124093	0.0007635	0.019327
ENSMUSG00000031963	Bmper	1288	1866	1217	2069	5620	3741	7648	5835	1.55166256	1.83E-09	2.78E-07
ENSMUSG00000052430	Bmpr1b	29	38	65	314	492	414	575	423	1.87620798	0.0014121	0.0309077
ENSMUSG00000032589	Bsn	45	67	66	58	163	294	237	332	1.82477262	1.65E-07	1.53E-05
ENSMUSG00000028392	Bspry	17	20	22	191	360	204	387	296	2.1107817	0.0014096	0.0308652
ENSMUSG00000040794	C1qtnf4	28	15	11	64	105	84	193	154	1.92658025	0.0002818	0.0087691
ENSMUSG00000015451	C4a	34	21	21	61	198	183	176	198	2.18796535	4.48E-08	4.76E-06
ENSMUSG00000026405	C4bp	63	8	1	13	68	10	2607	12	4.64205845	0.0013107	0.0290567
ENSMUSG00000079105	C7	4631	6085	6717	12437	20907	19111	17935	15174	1.02769117	2.00E-05	0.0010083
ENSMUSG00000015968	Cacna1d	101	104	136	343	589	579	645	427	1.45618276	0.000181	0.0061827
ENSMUSG00000020866	Cacna1g	290	446	187	517	1105	1134	925	887	1.20209935	0.0002569	0.0081835
ENSMUSG00000021991	Cacna2d3	13	12	42	85	189	180	285	248	2.33615706	1.42E-05	0.0007566
ENSMUSG00000020723	Cacng4	34	48	25	68	252	235	396	226	2.3823186	2.16E-09	3.23E-07
ENSMUSG00000032076	Cadm1	221	230	249	766	1338	1747	1847	1301	1.82949657	1.26E-06	9.08E-05
ENSMUSG00000028558	Calr4	22	12	30	28	324	216	485	370	3.65084919	8.22E-18	5.07E-15
ENSMUSG00000057897	Camk2b	86	71	68	411	648	459	895	892	1.96025715	0.0001953	0.0065661
ENSMUSG00000038357	Camp	1	7	0	2	739	106	658	649	7.4125661	6.99E-20	5.87E-17
ENSMUSG00000021373	Cap2	35	58	124	102	403	172	190	227	1.39112136	0.0016433	0.0346176
ENSMUSG00000031981	Capn9	13	21	19	15	249	137	162	215	3.20222714	6.39E-15	2.67E-12
ENSMUSG00000027556	Car1	67	62	58	45	321	242	264	348	2.0458813	1.76E-09	2.69E-07
ENSMUSG00000032373	Car12	12	41	28	32	217	39	364	474	3.01727083	2.87E-06	0.0001892

ENSMUSG00000027562	Car2	93	130	107	340	891	971	1070	905	2.25955874	4.45E-09	6.12E-07
ENSMUSG00000043541	Casc1	22	6	13	109	168	134	237	164	2.0030824	0.0024299	0.0463321
ENSMUSG00000060227	Casc4	290	335	398	702	963	922	1559	1050	1.1196467	0.0004713	0.0131755
ENSMUSG00000033597	Caskin1	32	40	48	52	118	106	127	91	1.08059209	0.0013238	0.0293344
ENSMUSG00000024039	Cbs	169	139	133	200	472	346	487	576	1.27584235	4.28E-05	0.0018957
ENSMUSG00000047696	Ccdc144b	57	21	16	21	261	266	204	243	2.75827695	1.31E-09	2.09E-07
ENSMUSG00000039492	Ccdc27	13	18	20	76	124	88	132	103	1.57548739	0.0021173	0.0418238
ENSMUSG00000045106	Ccdc73	39	50	47	59	95	124	159	113	1.04364506	0.0020435	0.0407424
ENSMUSG00000041117	Ccdc8	284	220	592	505	938	951	996	1108	1.05608091	0.0008984	0.0219207
ENSMUSG00000050677	Ccdc96	20	19	21	75	118	94	213	188	1.93872301	0.0001213	0.004453
ENSMUSG00000035352	Ccl12	37	41	39	52	105	147	179	120	1.41274752	3.79E-05	0.0017147
ENSMUSG00000071005	Ccl19	29	15	65	69	147	210	239	152	1.8056632	5.13E-05	0.0021933
ENSMUSG00000073888	Ccl27a	54	48	42	45	183	179	198	172	1.65503279	2.63E-07	2.28E-05
ENSMUSG00000028195	Ccn1	382	402	1074	1055	1705	1568	1452	2764	1.1163057	0.0017891	0.0368905
ENSMUSG00000005124	Ccn4	10	21	57	67	146	111	219	156	1.78795678	0.0004343	0.0123142
ENSMUSG00000047139	Cd24a	579	587	538	1901	2650	2391	2993	1823	1.19766981	0.0008597	0.0211456
ENSMUSG00000028459	Cd72	199	127	179	163	422	1709	326	350	1.72434764	0.0003237	0.0097356
ENSMUSG00000048895	Cdk5r1	52	38	68	98	137	215	229	207	1.34681574	0.0001914	0.006456
ENSMUSG00000020990	Cdkl1	23	25	31	53	263	67	75	82	1.63669666	0.001783	0.036791
ENSMUSG00000068740	Celsr2	472	452	234	938	1381	2352	1563	2197	1.55462214	1.41E-05	0.0007517
ENSMUSG00000040729	Cep126	97	91	71	280	339	329	498	419	1.30121667	0.0019238	0.0389164
ENSMUSG00000044948	Cfap43	66	65	72	359	393	362	865	560	1.72670595	0.0012173	0.0276035
ENSMUSG00000078490	Cfap74	103	77	53	258	398	398	479	411	1.51895186	0.0004262	0.0121537
ENSMUSG00000040809	Chil3	55	100	224	103	356	628	125	564	1.51200164	0.0024336	0.0463509
ENSMUSG00000024843	Chka	370	377	450	880	1357	1321	1512	948	1.04031179	0.0008128	0.0202771
ENSMUSG00000030077	Chl1	49	31	46	65	206	382	119	259	2.03876511	6.20E-07	4.87E-05
ENSMUSG00000022041	Chrna2	32	60	80	433	683	1002	577	518	1.96449673	0.0010785	0.0253454
ENSMUSG00000057337	Chst3	79	84	93	176	308	748	248	310	1.6017968	7.18E-05	0.0028919
ENSMUSG00000024526	Cidea	43	31	86	53	200	152	199	298	1.73044944	1.52E-05	0.0008016
ENSMUSG00000051159	Cited1	5	0	5	9	909	10	28	5	5.41112104	0.0001174	0.0043473
ENSMUSG00000022512	Cldn1	22	22	28	91	202	132	159	131	1.69207336	0.0003564	0.010474
ENSMUSG00000050520	Cldn8	1	7	0	0	530	0	171	0	6.07642369	0.001168	0.0267037
ENSMUSG00000022949	Clic6	183	206	73	1188	2035	2586	3063	2294	2.36194595	5.81E-05	0.002435
ENSMUSG00000022037	Clu	5737	3552	3961	5347	20820	26841	37092	23460	2.24546283	3.74E-22	4.23E-19

ENSMUSG00000022225	Cma1	394	407	394	622	1463	1813	2393	1064	1.60315475	7.33E-08	7.44E-06
ENSMUSG00000005864	Cnga2	40	44	26	104	203	105	224	143	1.40125198	0.0021651	0.0424799
ENSMUSG00000022025	Cnmd	28	58	22	28	254	495	457	280	3.12403135	1.88E-13	6.04E-11
ENSMUSG00000001349	Cnn1	90	97	945	493	8128	1089	1285	1208	2.64890367	0.0003392	0.0100768
ENSMUSG00000017167	Cntnap1	485	365	289	389	1552	1158	1387	1566	1.59734132	2.97E-08	3.32E-06
ENSMUSG00000024330	Col11a2	49	43	40	50	172	239	144	155	1.65897529	6.09E-07	4.81E-05
ENSMUSG00000058806	Col13a1	12	25	64	122	200	156	292	186	1.67485604	0.0023016	0.0445143
ENSMUSG00000045672	Col27a1	323	281	206	433	2189	2180	3847	2955	2.88671344	8.67E-21	8.30E-18
ENSMUSG00000091345	Col6a5	7912	5620	7862	12445	24325	22185	23967	19134	1.13222186	1.09E-07	1.05E-05
ENSMUSG00000025650	Col7a1	61	48	44	76	313	205	201	162	1.66119379	4.48E-06	0.0002796
ENSMUSG00000056174	Col8a2	66	96	214	558	747	670	909	779	1.50828892	0.002471	0.0468756
ENSMUSG00000031849	Comp	14	19	80	72	1400	1801	1628	1891	4.93125836	5.62E-24	7.42E-21
ENSMUSG00000025488	Cox8b	35	19	55	41	147	185	243	195	2.07952829	6.53E-08	6.69E-06
ENSMUSG00000001865	Cpa3	328	349	400	649	1564	1686	2367	1251	1.71855902	7.53E-09	9.91E-07
ENSMUSG00000025196	Cpn1	3	2	0	35	69	62	196	81	3.12413091	0.0004815	0.0134052
ENSMUSG00000024008	Cpne5	10	7	5	20	105	63	295	60	3.36027718	1.31E-07	1.24E-05
ENSMUSG00000025431	Crisp1	20	184	9	27	22273	2	1331	17	6.33651694	0.0002763	0.0086484
ENSMUSG00000025952	Crygc	0	0	0	0	1218	0	0	0	11.1737307	0.0001983	0.0066575
ENSMUSG00000030905	Crym	56	51	38	75	169	208	220	142	1.453675	2.46E-05	0.001199
ENSMUSG00000042109	Csdc2	81	105	127	273	613	444	703	574	1.74459781	4.20E-06	0.0002647
ENSMUSG00000000416	Cttnbp2	33	41	79	82	226	129	235	177	1.45225801	0.000241	0.0077665
ENSMUSG00000026726	Cubn	25	20	39	59	109	139	104	134	1.49391531	0.0001231	0.0045079
ENSMUSG00000031778	Cx3cl1	380	312	244	687	1239	1214	1869	1134	1.47617035	1.17E-05	0.0006406
ENSMUSG00000052336	Cx3cr1	201	88	138	292	719	988	403	503	1.57171724	6.87E-05	0.0027968
ENSMUSG00000019590	Cyb561	352	329	376	1201	1475	1044	3156	1239	1.37225418	0.0018258	0.0374105
ENSMUSG00000024087	Cyp1b1	2765	3111	2660	3374	8590	11906	9135	8430	1.37737286	1.71E-14	6.77E-12
ENSMUSG00000025197	Cyp2c23	27	13	36	11	84	119	68	83	1.71492713	0.0001419	0.0050592
ENSMUSG00000052974	Cyp2f2	106	52	4	67	303	297	354	297	2.13117002	0.000433	0.0122851
ENSMUSG00000085772	D630024D03Rik	24	17	18	35	116	107	83	98	1.81803472	1.86E-06	0.0001285
ENSMUSG00000085171	D830026I12Rik	221	194	245	199	661	659	623	709	1.33594274	4.86E-07	3.96E-05
ENSMUSG00000032500	Dclk3	72	57	152	269	580	358	656	677	1.8139058	4.97E-05	0.0021392
ENSMUSG00000022129	Dct	39	35	32	46	158	85	145	156	1.56301116	3.22E-05	0.0015036
ENSMUSG00000028194	Ddah1	81	68	198	636	863	782	1024	1185	1.75443387	0.0011078	0.0258154
ENSMUSG00000056544	Defb21	0	0	0	0	0	0	538	0	9.94749193	0.0004023	0.011573

ENSMUSG00000074681	Defb23	0	1	1	5	5	4	1590	2	7.52102364	4.36E-05	0.0019205
ENSMUSG00000075571	Defb30	27	2	2	1	179	3	2095	0	5.78402707	0.0008878	0.0217094
ENSMUSG00000054763	Defb42	27	4	3	14	898	8	997	5	5.01766286	8.85E-05	0.0034429
ENSMUSG00000009092	Derl3	84	51	55	77	203	755	246	195	2.05094093	7.74E-06	0.0004471
ENSMUSG00000026208	Des	1184	1101	2070	1780	6985	3234	2949	2581	1.09946082	0.0004446	0.0125472
ENSMUSG00000022861	Dgkg	24	31	37	85	241	240	299	244	2.27236966	2.06E-08	2.43E-06
ENSMUSG00000020834	Dhrs13	57	39	46	55	133	152	110	124	1.10132668	0.0008338	0.0206901
ENSMUSG00000040035	Disp2	74	54	369	149	607	410	865	583	1.68847636	0.0007943	0.0199224
ENSMUSG00000038060	Dlec1	107	77	55	379	682	612	719	723	1.90308193	0.0001056	0.0039705
ENSMUSG00000003279	Dlgap1	8	4	6	30	181	62	167	115	3.20826185	1.29E-07	1.22E-05
ENSMUSG00000020871	Dlx4	18	22	12	39	94	113	73	94	1.75129759	2.44E-05	0.0011901
ENSMUSG00000005237	Dnah2	29	21	19	220	297	362	419	325	2.05271063	0.0014749	0.0319927
ENSMUSG00000041144	Dnah7b	46	50	41	200	281	277	404	303	1.66316271	0.0005377	0.0145903
ENSMUSG00000033826	Dnah8	40	29	50	122	105	314	185	181	1.42524578	0.0021419	0.0421735
ENSMUSG00000030882	Dnhd1	205	251	154	245	595	915	675	718	1.45948224	4.64E-07	3.83E-05
ENSMUSG00000039716	Dock3	12	18	16	7	133	92	144	178	3.06933418	8.36E-12	1.92E-09
ENSMUSG00000030584	Dpf1	66	34	78	97	227	188	198	218	1.32696627	0.0002219	0.0072934
ENSMUSG00000061576	Dpp6	1	5	0	43	503	25	54	50	3.51191268	0.0025479	0.0480043
ENSMUSG00000025478	Dpysl4	12	15	11	20	736	466	756	815	5.29696111	6.01E-39	2.78E-35
ENSMUSG00000000223	Drp2	19	27	51	107	162	167	154	151	1.38899364	0.0025734	0.0483713
ENSMUSG00000018648	Dusp14	46	26	45	141	201	223	320	275	1.73229715	0.0001942	0.0065373
ENSMUSG00000042662	Dusp15	10	23	18	48	113	51	121	60	1.5497961	0.0022667	0.0440219
ENSMUSG00000037784	Dzip1l	144	145	193	493	650	607	827	810	1.32291589	0.0005818	0.0155394
ENSMUSG00000018983	E2f2	145	78	127	119	395	575	185	334	1.35908432	0.0003246	0.0097514
ENSMUSG00000026051	Ecrg4	16	40	59	174	572	389	914	892	3.03904566	9.03E-08	8.91E-06
ENSMUSG00000041220	Elovl6	395	278	417	473	1219	777	1025	1005	1.09345865	0.0001212	0.0044525
ENSMUSG00000021728	Emb	271	284	397	525	2761	2571	1388	2273	2.33472149	6.76E-15	2.80E-12
ENSMUSG00000022468	Endou	105	93	149	139	182	527	389	328	1.25283677	0.0003807	0.0110384
ENSMUSG00000028434	Epb41l4b	72	53	57	235	391	304	382	328	1.50635071	0.0008477	0.0209429
ENSMUSG00000029869	Ephb6	512	361	324	361	1103	932	802	1139	1.05402125	0.0002977	0.0091176
ENSMUSG00000053367	Epp13	37	0	0	14	11	14	2835	28	5.49127556	0.0013017	0.0288671
ENSMUSG00000019936	Epyc	47	38	13	117	170	148	292	266	1.7693516	0.0009247	0.0224252
ENSMUSG00000062209	Erbb4	28	60	10	82	113	232	335	383	2.28229328	5.70E-05	0.0023908
ENSMUSG00000084128	Esrp2	8	13	9	81	296	103	220	137	2.553081	0.0001467	0.0051907

ENSMUSG00000025932	Eya1	11	10	45	21	199	107	200	131	2.61434211	1.86E-07	1.69E-05
ENSMUSG00000010461	Eya4	111	89	121	71	399	236	430	354	1.56952564	7.63E-06	0.0004413
ENSMUSG00000026579	F5	331	507	229	352	838	1337	838	861	1.13222219	0.0003328	0.0099428
ENSMUSG00000052125	F730043M19Rik	287	445	278	473	1313	1355	778	1679	1.50422334	2.41E-06	0.000163
ENSMUSG00000028773	Fabp3	5	9	16	64	169	209	192	109	2.6088479	1.87E-05	0.0009562
ENSMUSG00000024665	Fads2	856	858	735	2357	5917	2451	3970	3163	1.44595251	5.59E-05	0.0023513
ENSMUSG00000021750	Fam107a	222	253	555	370	695	1382	1214	968	1.32151269	6.01E-05	0.0025052
ENSMUSG00000021234	Fam161b	85	67	87	130	240	202	210	242	1.00377545	0.0016421	0.034605
ENSMUSG00000035095	Fam167a	26	20	71	356	655	383	757	586	2.13275267	0.0016411	0.0345967
ENSMUSG00000041817	Fam169a	9	5	11	58	160	111	265	130	2.77437465	1.40E-05	0.0007429
ENSMUSG00000030207	Fam234b	195	226	259	396	778	560	697	614	1.03460341	0.0005055	0.0139006
ENSMUSG00000074505	Fat3	12	9	13	70	177	116	147	142	2.25243437	9.42E-05	0.003631
ENSMUSG00000034532	Fbxo16	8	9	10	43	103	78	139	123	2.41631477	6.82E-06	0.0004002
ENSMUSG00000041556	Fbxo2	9	58	43	24	488	389	272	214	3.04961578	2.61E-09	3.83E-07
ENSMUSG00000005339	Fcer1a	13	22	30	51	156	197	240	104	2.31434721	1.81E-07	1.66E-05
ENSMUSG00000048031	Fcrl5	31	8	13	18	79	789	7	33	3.29209519	0.0009688	0.0232311
ENSMUSG00000022871	Fetub	22	29	6	25	169	277	124	138	2.78322719	6.26E-09	8.36E-07
ENSMUSG00000027208	Fgf7	121	100	166	153	331	391	408	370	1.19143908	3.75E-05	0.0017052
ENSMUSG00000030849	Fgfr2	536	489	425	1082	1995	1436	1943	1722	1.22375353	7.10E-05	0.0028719
ENSMUSG00000034295	Fhod3	70	70	128	160	822	246	377	356	1.83104736	2.99E-05	0.0014033
ENSMUSG00000074971	Fibin	99	159	271	286	490	461	593	497	1.06490507	0.0018413	0.0375628
ENSMUSG00000024222	Fkbp5	1507	4524	2256	4079	9895	7890	14963	10654	1.53584172	6.87E-07	5.36E-05
ENSMUSG00000028354	Fmn2	11	8	7	27	84	137	112	89	2.70188019	5.24E-09	7.07E-07
ENSMUSG00000001334	Fndc5	206	195	35	300	885	753	1073	879	2.00023362	2.36E-05	0.001155
ENSMUSG00000038402	Foxf2	23	16	18	13	206	163	188	245	3.21924087	2.72E-16	1.41E-13
ENSMUSG00000027004	Frzb	5	15	76	24	134	174	147	203	2.19757167	0.0002096	0.0069892
ENSMUSG00000049307	Fut4	63	67	81	100	200	340	183	167	1.21874306	0.0002921	0.0089854
ENSMUSG00000078612	Fyb2	62	104	69	249	465	298	415	265	1.3252307	0.0024092	0.0460648
ENSMUSG00000038860	Garnl3	473	493	410	457	1233	1052	1556	1351	1.21218942	5.39E-06	0.0003278
ENSMUSG00000031451	Gas6	9287	11697	8216	13378	34204	25631	49176	48150	1.60844388	6.16E-11	1.27E-08
ENSMUSG00000038233	Gask1a	20	39	33	83	133	132	164	196	1.5791243	0.000235	0.0076296
ENSMUSG00000035540	Gc	13	0	0	2	22	10	791	28	5.44941792	0.0001841	0.0062464
ENSMUSG00000017943	Gdap1l1	6	6	4	56	80	109	113	116	2.30091343	0.0007162	0.0184056
ENSMUSG00000019359	Gdpd2	14	34	11	59	147	80	111	96	1.61943492	0.001502	0.0324108

ENSMUSG00000030703	Gdpd3	72	79	92	68	104	126	623	961	2.27412558	0.0001968	0.0066109
ENSMUSG00000040471	Ggt6	17	10	15	26	166	120	165	219	3.02620893	5.64E-13	1.61E-10
ENSMUSG00000050953	Gja1	1882	1935	2016	7648	8774	7497	12261	9298	1.24916758	0.0006708	0.0173937
ENSMUSG00000025059	Gk	209	336	302	1429	1595	1367	2675	1711	1.46141893	0.002189	0.0428438
ENSMUSG00000024827	Gldc	45	69	96	49	711	246	385	480	2.54774626	2.19E-09	3.26E-07
ENSMUSG00000028020	Glrb	20	17	37	19	95	106	93	65	1.65446761	2.86E-05	0.0013522
ENSMUSG00000067736	Gm10222	9	19	12	15	2135	14	18	17	5.06646473	0.0001267	0.0045966
ENSMUSG00000079644	Gm1110	46	2	0	8	49	39	1228	34	4.2370436	0.0011666	0.0267033
ENSMUSG00000085419	Gm11734	19	5	21	13	81	91	96	119	2.44531376	6.25E-08	6.46E-06
ENSMUSG00000086765	Gm11827	16	26	6	40	86	189	90	81	2.03181034	8.38E-05	0.0032947
ENSMUSG00000078502	Gm13212	64	86	107	85	168	271	211	215	1.04435065	0.0008397	0.0207721
ENSMUSG00000085591	Gm13479	17	11	8	13	107	148	122	176	3.18627566	3.39E-14	1.30E-11
ENSMUSG00000085767	Gm13563	35	39	40	56	99	129	127	123	1.20491711	0.0002582	0.0082011
ENSMUSG00000086746	Gm15222	39	49	42	48	96	155	137	122	1.21820957	0.0002509	0.0080169
ENSMUSG00000085971	Gm15411	19	8	10	48	117	144	143	187	2.53365314	7.17E-07	5.56E-05
ENSMUSG00000070858	Gm1673	47	32	71	42	110	121	196	101	1.17276373	0.0025217	0.0476077
ENSMUSG00000061331	Gm17132	35	69	47	55	116	162	169	164	1.2736192	0.0002792	0.0087227
ENSMUSG00000097183	Gm17501	25	22	62	44	104	115	143	95	1.30578563	0.0010229	0.0242443
ENSMUSG00000102504	Gm21955	17	17	36	74	112	99	243	139	1.79869009	0.0003751	0.0109015
ENSMUSG00000098041	Gm26981	40	21	24	46	118	106	92	120	1.44964466	0.0001443	0.0051258
ENSMUSG00000112466	Gm29674	26	13	11	32	109	121	170	123	2.38222432	2.77E-08	3.12E-06
ENSMUSG00000109603	Gm32389	24	18	47	42	93	155	81	103	1.43516932	0.0003228	0.0097135
ENSMUSG00000111147	Gm33699	71	79	114	69	180	258	169	251	1.07095219	0.0011739	0.0268169
ENSMUSG00000115762	Gm34907	51	81	67	67	260	186	265	404	1.7894235	9.96E-07	7.41E-05
ENSMUSG00000103839	Gm37607	21	28	16	30	102	106	83	105	1.7645551	2.21E-06	0.0001507
ENSMUSG00000105556	Gm43080	78	79	43	74	356	305	225	244	1.74282388	7.08E-07	5.50E-05
ENSMUSG00000106641	Gm43534	0	2	2	27	89	60	213	107	3.69613859	1.92E-05	0.0009771
ENSMUSG00000109005	Gm45221	17	36	21	37	66	85	118	74	1.33730074	0.0009989	0.0238398
ENSMUSG00000112342	Gm47031	141	209	228	235	483	528	591	575	1.14097908	4.70E-05	0.0020386
ENSMUSG00000111824	Gm47102	21	33	64	36	114	205	87	178	1.63402514	0.0001322	0.0047681
ENSMUSG00000113383	Gm47267	37	54	27	32	152	165	129	174	1.74016184	2.50E-06	0.000168
ENSMUSG00000113032	Gm47578	41	69	172	83	260	358	276	446	1.60720804	0.0001236	0.0045166
ENSMUSG00000112580	Gm47673	40	34	29	47	155	187	203	207	2.03352885	2.05E-09	3.08E-07
ENSMUSG00000114367	Gm48126	25	33	36	43	90	120	95	138	1.40815112	6.61E-05	0.0027098

ENSMUSG00000112349	Gm48132	27	34	59	35	162	207	174	181	1.93522319	3.33E-08	3.66E-06
ENSMUSG00000113393	Gm48145	46	42	77	46	131	180	128	225	1.36700224	0.0001834	0.0062388
ENSMUSG00000112049	Gm48269	15	25	31	33	110	159	167	216	2.36782769	1.55E-09	2.41E-07
ENSMUSG00000110779	Gm48271	58	100	89	121	211	250	232	217	1.02340966	0.0011678	0.0267037
ENSMUSG00000118125	Gm50387	36	26	27	40	93	91	92	145	1.42178865	0.0001431	0.0050901
ENSMUSG00000111290	Gm5122	3	9	8	9	128	118	96	108	3.65381277	1.18E-16	6.32E-14
ENSMUSG00000109857	Gm53058	42	29	18	18	207	134	144	147	2.25181972	1.19E-07	1.14E-05
ENSMUSG00000098050	Gm5345	6	17	29	29	133	210	124	148	2.64238123	2.86E-09	4.09E-07
ENSMUSG00000084950	Gm5577	50	86	49	87	243	142	228	227	1.35092121	0.0002573	0.0081896
ENSMUSG00000107092	Gm7993	22	30	23	60	47	130	152	106	1.40428551	0.0022366	0.0435903
ENSMUSG00000050157	Gm867	22	28	24	108	144	264	258	171	1.93928241	0.0001059	0.0039752
ENSMUSG00000090588	Gm9573	0	0	0	0	1129	0	0	0	11.0643058	0.0002105	0.0070106
ENSMUSG00000095332	Gm9821	67	92	111	107	237	254	264	247	1.12857592	0.0001564	0.0054835
ENSMUSG00000054618	Gm9951	71	51	107	122	217	140	350	278	1.23212635	0.0019121	0.0387929
ENSMUSG00000031748	Gnao1	486	458	920	777	2081	1811	2060	1546	1.23573182	5.88E-06	0.0003507
ENSMUSG00000021340	Gpld1	275	297	251	312	1144	782	1147	1399	1.69880721	6.36E-09	8.46E-07
ENSMUSG00000056679	Gpr173	48	37	98	84	180	166	294	252	1.47874202	0.0001532	0.005391
ENSMUSG00000068696	Gpr88	23	42	48	64	242	231	289	259	2.25760296	3.57E-10	6.34E-08
ENSMUSG00000072966	Gprasp2	35	35	42	303	414	329	719	379	1.93256304	0.0018231	0.0373833
ENSMUSG00000045441	Gprin3	332	338	533	268	640	1445	1118	905	1.17402683	0.0002339	0.0076106
ENSMUSG00000036292	Gramd1c	58	36	41	93	217	185	206	163	1.48250535	6.17E-05	0.0025565
ENSMUSG00000042942	Greb1l	125	231	99	209	432	411	493	580	1.24223281	0.0003689	0.0107441
ENSMUSG00000071723	Gspt2	200	215	188	413	552	545	938	607	1.10959506	0.0009216	0.0223607
ENSMUSG00000067235	H2-Q10	814	624	181	392	2821	2962	2871	3739	2.30079782	2.80E-09	4.02E-07
ENSMUSG00000027360	Hdc	136	163	105	511	491	736	851	664	1.32511787	0.0023423	0.0450337
ENSMUSG00000038422	Hdhd3	114	105	308	142	358	195	731	948	1.48865826	0.0026195	0.0489721
ENSMUSG00000027875	Hmgcs2	3692	5923	4242	4119	10176	19183	7828	9040	1.04277021	4.42E-05	0.0019416
ENSMUSG00000025813	Homer2	90	75	61	115	296	107	325	496	1.58138492	0.0004949	0.013682
ENSMUSG00000028572	Hook1	92	94	54	393	651	543	837	580	1.80316195	0.0003244	0.0097514
ENSMUSG00000028785	Hpca	304	273	57	333	1175	1069	1577	1004	2.01977897	4.90E-06	0.0003015
ENSMUSG00000029445	Hpd	40	56	33	25	108	134	90	105	1.18767264	0.0019403	0.0390791
ENSMUSG00000047759	Hs3st3a1	23	43	43	48	131	133	89	114	1.28889331	0.0003537	0.0104279
ENSMUSG00000031891	Hsd11b2	18	32	13	24	119	75	71	309	2.44153986	1.32E-05	0.000705
ENSMUSG00000030825	Hsd17b14	114	88	33	114	341	205	218	342	1.37674582	0.0012867	0.0286147

ENSMUSG00000000732	Icosl	379	404	596	744	1071	2661	1784	1296	1.38907946	8.56E-06	0.0004884
ENSMUSG00000062157	Ifnlr1	17	22	12	53	112	178	140	121	2.12339624	2.49E-06	0.0001675
ENSMUSG00000030323	Ift122	629	505	521	869	1850	1072	1644	1553	1.0084995	0.0004493	0.0126435
ENSMUSG00000039323	Igfbp2	181	352	350	231	1003	553	1058	1325	1.54934815	1.29E-05	0.000695
ENSMUSG00000076614	Ighg1	469	156	212	31	1768	2996	762	2699	2.89387836	2.46E-06	0.0001652
ENSMUSG00000094561	Ighv1-22	17	6	5	21	746	130	22	66	4.03655408	9.27E-06	0.0005218
ENSMUSG00000096490	Igkv10-94	59	39	50	43	79	476	200	210	1.99986727	2.69E-05	0.0012865
ENSMUSG00000096422	Igkv12-44	131	57	51	50	211	249	236	202	1.31242639	0.0011445	0.026373
ENSMUSG00000076526	Igkv12-98	41	18	26	33	1111	64	29	178	3.29923403	0.0001408	0.0050285
ENSMUSG00000076514	Igkv17-121	76	32	80	22	282	703	142	74	2.16082939	0.0005392	0.0146237
ENSMUSG00000095335	Igkv3-5	149	16	130	54	365	2466	70	414	2.87419054	0.0004352	0.0123322
ENSMUSG00000076547	Igkv4-70	35	13	7	31	177	103	45	93	1.98726856	0.0004403	0.0124507
ENSMUSG00000076540	Igkv4-80	13	7	29	13	39	561	24	57	3.08770317	0.0003001	0.0091745
ENSMUSG00000095794	Igkv6-17	102	31	43	16	857	430	112	259	2.78679382	1.88E-05	0.00096
ENSMUSG00000076587	Igkv6-20	14	3	4	3	22	498	29	15	4.10660913	9.27E-05	0.003581
ENSMUSG00000076586	Igkv8-21	18	23	57	21	115	74	70	109	1.35929779	0.0026481	0.049357
ENSMUSG00000031111	Igsf1	17	21	21	46	259	193	185	355	2.97788647	3.96E-12	9.76E-10
ENSMUSG00000042035	Igsf3	284	212	309	387	753	645	771	776	1.03142975	0.0001876	0.0063427
ENSMUSG00000034275	Igsf9b	47	46	50	118	282	233	166	167	1.43354986	0.0002778	0.0086835
ENSMUSG00000015966	Il17rb	99	198	50	287	578	1045	483	933	1.97290674	2.22E-05	0.0011004
ENSMUSG00000043088	Il17re	278	228	181	301	1048	926	1097	1359	1.87948236	1.77E-10	3.43E-08
ENSMUSG00000044244	Il20rb	108	110	128	349	490	438	752	565	1.44234805	0.0003003	0.0091745
ENSMUSG00000039760	Il22ra2	10	15	21	11	114	149	71	118	2.68284368	2.96E-10	5.39E-08
ENSMUSG00000032968	Inha	47	26	40	79	205	137	142	85	1.29743537	0.0021192	0.0418465
ENSMUSG00000026638	Irf6	41	26	89	236	558	247	400	260	1.68483365	0.0025806	0.0484411
ENSMUSG00000060969	Irx1	1	8	1	12	347	11	66	26	4.10682926	6.60E-05	0.0027098
ENSMUSG00000019139	Isyna1	808	683	745	1319	2988	3781	2931	2102	1.44482889	1.26E-08	1.56E-06
ENSMUSG00000037254	Itih2	13	26	54	65	244	295	297	183	2.42543695	4.96E-08	5.21E-06
ENSMUSG00000006522	Itih3	7	27	46	62	132	223	140	156	1.93048762	9.51E-05	0.0036503
ENSMUSG00000021922	Itih4	148	72	48	112	752	1413	847	1014	3.08662809	3.18E-15	1.37E-12
ENSMUSG00000031239	Itm2a	75	143	243	325	725	914	641	546	1.58255828	3.97E-05	0.0017809
ENSMUSG00000040724	Kcna2	194	227	201	245	847	771	699	666	1.494198	1.56E-08	1.91E-06
ENSMUSG00000062785	Kcnc3	663	643	338	596	1494	1899	1783	1622	1.29381186	4.99E-06	0.0003068
ENSMUSG00000040896	Kcnd3	161	163	132	434	798	489	707	509	1.23841462	0.0014786	0.0320419

ENSMUSG00000090122	Kcne1l	54	26	130	74	560	272	985	1058	3.09404227	3.19E-09	4.53E-07
ENSMUSG00000047330	Kcne4	157	230	321	410	674	863	548	686	1.0369427	0.0009419	0.0227632
ENSMUSG00000051726	Kcnf1	1	1	0	56	89	106	257	212	3.31951838	0.0017969	0.0370032
ENSMUSG00000074575	Kcng1	13	23	46	64	431	106	172	118	2.27256811	6.35E-05	0.0026175
ENSMUSG00000059852	Kcng2	34	12	22	25	109	60	82	105	1.65316366	0.0001521	0.0053578
ENSMUSG00000038319	Kcnh2	116	118	536	272	1385	513	1235	1534	1.93126488	6.18E-05	0.0025601
ENSMUSG00000037624	Kcnk2	178	126	204	340	725	508	714	650	1.3560956	4.36E-05	0.0019205
ENSMUSG00000063142	Kcnma1	47	50	103	135	271	231	284	228	1.34185316	0.0003535	0.0104276
ENSMUSG00000058740	Kcnt1	60	121	55	123	1046	1690	1447	1095	3.5781981	1.91E-23	2.41E-20
ENSMUSG00000051727	Kctd14	25	27	20	172	379	343	546	377	2.52382933	2.09E-05	0.001047
ENSMUSG00000014602	Kif1a	22	31	59	74	227	119	123	105	1.37568147	0.0018941	0.0384691
ENSMUSG00000022629	Kif21a	359	634	845	539	1559	1430	1977	2058	1.28854062	1.21E-05	0.0006612
ENSMUSG00000026494	Kif26b	10	4	20	114	239	133	192	213	2.18993061	0.0022963	0.0444265
ENSMUSG00000023999	Kif6	2	5	10	59	129	72	190	227	2.82004206	0.0002043	0.006838
ENSMUSG00000032489	Kif9	71	59	53	272	359	336	453	359	1.48346114	0.0018086	0.0371691
ENSMUSG00000042155	Klhl23	102	115	178	144	376	272	360	326	1.03548256	0.0007897	0.0198227
ENSMUSG00000020627	Klhl29	191	245	364	365	840	721	1110	1045	1.4086406	2.96E-06	0.0001938
ENSMUSG00000090799	Klhl33	151	165	119	125	363	233	375	410	1.0127902	0.0022499	0.0437876
ENSMUSG00000022875	Kng1	0	2	1	1	138	263	105	122	6.83177594	2.49E-26	4.45E-23
ENSMUSG00000060459	Kng2	31	46	24	31	677	995	765	715	4.26414254	2.34E-34	8.10E-31
ENSMUSG00000032796	Lama1	43	53	13	118	1000	651	1002	838	3.6814044	4.32E-13	1.28E-10
ENSMUSG00000026639	Lamb3	152	169	241	194	749	479	619	954	1.62134923	4.74E-07	3.88E-05
ENSMUSG00000026479	Lamc2	79	77	88	373	1002	702	839	772	2.19137713	3.11E-06	0.0002024
ENSMUSG00000033595	Lgi3	116	106	45	147	743	309	628	602	2.18801134	2.16E-07	1.93E-05
ENSMUSG00000049556	Lingo1	25	15	15	30	64	43	188	58	1.7729719	0.0009529	0.0229482
ENSMUSG00000053846	Lipg	23	98	52	136	238	604	880	126	2.29209088	0.0001234	0.0045152
ENSMUSG00000048814	Lonrf2	41	52	65	61	192	226	187	242	1.66674393	1.54E-07	1.44E-05
ENSMUSG00000016239	Lonrf3	76	59	111	154	409	300	530	339	1.72047979	2.30E-06	0.0001562
ENSMUSG00000024529	Lox	1393	1419	3085	2516	4209	6839	5806	6155	1.17658426	1.79E-06	0.0001238
ENSMUSG00000036832	Lpar3	26	33	62	50	133	116	179	173	1.5464386	4.39E-05	0.0019285
ENSMUSG00000027253	Lrp4	155	159	121	513	751	1004	1010	632	1.5777459	0.0001374	0.0049289
ENSMUSG00000090291	Lrrc10b	11	28	61	149	249	180	283	250	1.72889872	0.0024946	0.0471952
ENSMUSG00000052316	Lrrc15	5	11	10	16	113	132	164	348	3.89557127	1.19E-13	3.97E-11
ENSMUSG00000049939	Lrrc4	140	66	119	83	479	265	440	432	1.70114693	4.24E-06	0.0002673

ENSMUSG00000046807	Lrrc75b	16	34	26	420	731	523	1103	609	2.38220954	0.0019207	0.0388938
ENSMUSG00000021090	Lrrc9	46	35	39	95	130	151	164	133	1.15355565	0.0023536	0.0452196
ENSMUSG00000060780	Lrrtm1	30	17	21	44	662	187	535	546	3.84888489	3.02E-15	1.31E-12
ENSMUSG00000055003	Lrtm2	0	0	4	3	153	342	514	389	7.27779527	5.17E-29	1.37E-25
ENSMUSG00000103409	Lsmem2	19	7	23	56	94	96	81	87	1.51336755	0.0021962	0.0429395
ENSMUSG00000002020	Ltbp2	1935	2130	2131	2617	8517	6778	17504	12030	2.07270252	1.88E-14	7.35E-12
ENSMUSG00000028701	Lurap1	150	130	127	129	407	382	415	482	1.36104306	2.55E-06	0.0001705
ENSMUSG00000027375	Mal	17	35	67	174	513	182	502	209	2.04742351	0.0005916	0.0157293
ENSMUSG00000024479	Mal2	71	21	65	137	605	146	338	257	1.95932928	0.000269	0.0084527
ENSMUSG00000040147	Maob	2069	2217	1808	1863	7396	4509	8587	6987	1.50192637	1.28E-10	2.54E-08
ENSMUSG00000052727	Map1b	1163	1126	1337	2848	4426	3741	4367	3751	1.07180426	5.51E-05	0.0023265
ENSMUSG00000051590	Map3k19	22	34	15	118	116	217	185	228	1.72484693	0.0016731	0.0350444
ENSMUSG00000041020	Map7d2	31	32	38	35	255	118	355	165	2.43802557	1.06E-08	1.34E-06
ENSMUSG00000004864	Mapk13	31	27	13	152	207	188	336	203	1.82921955	0.0022524	0.0438003
ENSMUSG00000022887	Masp1	168	136	175	117	454	446	364	373	1.15880249	9.88E-05	0.0037694
ENSMUSG00000007480	Mc5r	35	49	10	56	146	146	117	122	1.52678283	0.0009024	0.0219798
ENSMUSG00000086392	Mccc1os	38	18	25	27	96	66	60	105	1.30949696	0.0015839	0.033738
ENSMUSG00000061068	Mcpt4	451	396	442	795	2014	2151	3669	1770	1.93016315	2.83E-10	5.16E-08
ENSMUSG00000032717	Mdfi	54	41	51	55	159	151	140	157	1.30577967	3.96E-05	0.0017785
ENSMUSG00000043557	Mdga1	36	20	47	75	175	118	242	314	2.00488341	1.11E-05	0.0006124
ENSMUSG00000036466	Megf11	17	26	16	66	80	90	129	116	1.47071063	0.0018453	0.0375888
ENSMUSG00000042436	Mfap4	100	118	392	423	1251	798	2190	1908	2.34342532	5.80E-07	4.63E-05
ENSMUSG00000028655	Mfsd2a	2	0	0	13	964	61	161	104	6.19515376	4.79E-08	5.06E-06
ENSMUSG00000054942	Miga1	233	261	266	536	831	829	1137	941	1.26289115	5.07E-05	0.0021762
ENSMUSG00000039533	Mmd2	30	45	12	308	581	289	722	530	2.21838569	0.001948	0.0392048
ENSMUSG00000020000	Moxd1	9	6	57	45	171	97	120	102	1.83264163	0.0018204	0.037341
ENSMUSG00000052373	Mpp3	121	140	149	269	757	718	832	615	1.83528776	3.85E-09	5.38E-07
ENSMUSG00000016386	Mpped2	9	3	12	15	181	187	113	140	3.70076968	4.04E-16	2.00E-13
ENSMUSG00000032092	Mpzl2	56	69	33	183	365	411	376	394	1.91739333	1.79E-05	0.0009203
ENSMUSG00000070305	Mpzl3	74	84	80	107	249	236	212	192	1.07978549	0.0003345	0.0099739
ENSMUSG00000070547	Mrgprb1	69	63	78	105	297	475	502	279	2.01014662	1.62E-09	2.50E-07
ENSMUSG00000050425	Mrgprb2	19	4	14	33	90	115	163	63	2.34203878	9.19E-06	0.0005186
ENSMUSG00000074109	Mrgprx2	22	20	16	66	136	261	232	126	2.32449006	1.18E-06	8.60E-05
ENSMUSG00000024680	Ms4a2	25	13	25	38	92	114	181	60	1.86136114	4.24E-05	0.0018826

ENSMUSG00000026100	Mstn	3	0	7	1	350	198	355	540	6.71337686	7.39E-27	1.37E-23
ENSMUSG00000005667	Mthfd2	148	232	198	327	1850	837	2858	1617	2.72538338	1.98E-12	5.26E-10
ENSMUSG00000038065	Mturn	2782	2620	3902	3575	5602	7414	9089	9542	1.01773235	1.17E-06	8.49E-05
ENSMUSG00000066108	Muc5b	12	0	1	15	24	14	1329	15	5.33754584	0.0002247	0.007364
ENSMUSG00000066154	Mup3	6	9	14	3	307	110	120	67	3.94279569	1.72E-10	3.33E-08
ENSMUSG00000058523	Mup5	0	0	0	0	10	3	1005	689	11.6270588	1.78E-09	2.70E-07
ENSMUSG00000019982	Myb	26	26	24	200	305	267	345	301	1.91810521	0.0013997	0.0307223
ENSMUSG00000020061	Mybpc1	4	2	8	4	553	46	20	17	4.87593991	4.78E-06	0.0002952
ENSMUSG00000056328	Myh1	26	29	32	16	87	32	119	163	1.68133899	0.0011588	0.0266234
ENSMUSG00000018830	Myh11	3060	4040	11214	7438	37507	13056	11220	12705	1.29283491	0.0014574	0.0316886
ENSMUSG00000027470	Mylk2	1	3	53	32	222	100	345	226	3.10926818	8.30E-05	0.0032712
ENSMUSG00000009214	Mymk	0	2	1	0	179	51	280	417	7.80549224	1.25E-18	8.99E-16
ENSMUSG00000072720	Myo18b	3	5	18	42	322	60	81	85	2.80527876	0.0001689	0.0058501
ENSMUSG00000033590	Myo5c	24	12	17	164	564	638	567	680	3.27029559	5.79E-07	4.63E-05
ENSMUSG00000000197	Nalcn	67	95	98	239	380	330	645	460	1.61210897	7.87E-05	0.0031331
ENSMUSG00000020572	Nampt	3324	3936	3098	3392	9196	9210	18352	10617	1.49255197	6.95E-10	1.18E-07
ENSMUSG00000052512	Nav2	75	98	128	652	993	903	1303	1123	1.96051389	0.0002695	0.0084633
ENSMUSG00000039542	Ncam1	24	30	52	107	510	276	350	256	2.46727858	1.11E-07	1.07E-05
ENSMUSG00000053702	Nebl	46	72	57	114	254	211	228	252	1.4401289	3.88E-05	0.0017519
ENSMUSG00000021365	Nedd9	1016	1256	1481	1484	5153	2602	7894	8472	1.94221109	6.35E-10	1.08E-07
ENSMUSG00000006435	Neurl1a	23	22	33	88	156	117	114	142	1.42540614	0.0017177	0.0357095
ENSMUSG00000026442	Nfasc	72	52	43	84	221	373	158	160	1.54991909	6.30E-05	0.0026018
ENSMUSG00000021032	Ngb	52	40	21	22	226	157	178	151	2.08422303	7.70E-07	5.94E-05
ENSMUSG00000038879	Nipal2	38	33	38	127	284	200	257	253	1.82754449	3.90E-05	0.0017593
ENSMUSG00000041596	Nlrp5-ps	31	18	19	30	85	77	110	83	1.56539998	4.44E-05	0.001948
ENSMUSG00000040998	Npnt	94	81	248	335	692	705	610	615	1.5418014	0.0001356	0.0048724
ENSMUSG00000025582	Nptx1	5	22	62	45	455	93	171	37	2.27061234	0.0021277	0.0419647
ENSMUSG00000060275	Nrg2	30	34	26	75	358	335	500	375	2.97906842	6.28E-14	2.23E-11
ENSMUSG00000039114	Nrn1	20	10	20	17	190	180	161	123	2.98860238	2.08E-14	8.03E-12
ENSMUSG00000024109	Nrxn1	7	19	17	19	108	161	220	211	3.20822676	2.40E-13	7.54E-11
ENSMUSG00000029126	Nsg1	216	238	349	297	795	500	983	944	1.28408863	4.31E-05	0.001902
ENSMUSG00000117406	Ntn3	85	66	113	88	361	359	388	461	1.87456763	1.37E-09	2.16E-07
ENSMUSG00000059146	Ntrk3	157	227	401	346	1679	1610	1315	1859	2.2494345	6.39E-13	1.80E-10
ENSMUSG00000020591	Ntsr2	65	28	60	16	169	303	275	226	2.20351649	1.52E-06	0.0001067

ENSMUSG00000073295	Nudt11	13	11	9	64	170	54	136	75	1.93787762	0.0019757	0.039647
ENSMUSG00000045348	Nyap1	22	38	34	123	183	187	206	170	1.53162718	0.0011113	0.0258649
ENSMUSG00000027848	Olfml3	443	713	1023	1593	3029	1743	3629	2086	1.23024942	0.0003316	0.0099202
ENSMUSG00000059729	Olfr1385	23	21	36	20	89	107	127	122	1.86180871	5.86E-07	4.66E-05
ENSMUSG00000061039	Olfr920	53	44	40	49	111	207	156	133	1.39604165	4.27E-05	0.001893
ENSMUSG00000048368	Omd	78	69	72	118	292	285	569	363	1.8865817	1.25E-07	1.19E-05
ENSMUSG00000010311	Optc	34	29	45	22	209	113	118	125	1.83329044	5.87E-06	0.0003506
ENSMUSG00000061540	Orm2	65	60	54	67	261	226	284	278	1.80362089	8.04E-09	1.04E-06
ENSMUSG00000022330	Osr2	50	59	27	163	438	283	341	268	1.8992962	6.77E-05	0.0027632
ENSMUSG00000005950	P2rx5	71	93	117	77	423	699	254	679	2.22239723	5.10E-09	6.93E-07
ENSMUSG00000040276	Pacsin1	9	10	25	88	114	196	191	118	1.98648656	0.0009019	0.0219792
ENSMUSG00000028927	Padi2	170	139	116	293	345	561	529	380	1.05078052	0.0021459	0.0421974
ENSMUSG00000005447	Pafah1b3	135	92	114	212	340	356	398	273	1.02743045	0.0017042	0.0354822
ENSMUSG00000020051	Pah	3	3	6	9	113	349	186	158	4.9318346	1.09E-20	1.02E-17
ENSMUSG00000028370	Pappa	65	139	75	132	330	363	242	207	1.17941265	0.0010872	0.0255181
ENSMUSG00000073530	Pappa2	51	97	107	142	327	354	256	220	1.26619568	0.0002949	0.0090542
ENSMUSG00000041423	Paqr6	45	49	72	110	274	126	202	229	1.34085011	0.0007252	0.0185699
ENSMUSG00000022439	Parvg	122	169	184	159	342	665	583	672	1.54064655	8.32E-07	6.33E-05
ENSMUSG00000061859	Patj	303	272	140	686	1184	1240	1192	1010	1.4524533	0.0001795	0.0061436
ENSMUSG00000073591	Pcdhb22	143	137	131	173	333	330	446	463	1.14565765	0.0001165	0.0043273
ENSMUSG00000102543	Pcdhgc5	20	20	19	45	102	85	91	132	1.71090894	2.81E-05	0.0013332
ENSMUSG00000038370	Pcp4l1	106	167	464	624	1280	716	1166	935	1.36201795	0.0024944	0.0471952
ENSMUSG00000075270	Pde11a	63	162	74	176	604	791	545	682	2.17719681	5.71E-09	7.69E-07
ENSMUSG00000004347	Pde1c	9	12	11	21	618	43	37	37	3.55557997	6.15E-05	0.0025523
ENSMUSG00000054728	Phactr1	290	297	307	382	846	1018	1712	820	1.49752821	9.63E-07	7.21E-05
ENSMUSG00000057457	Phex	1	4	1	2	331	193	726	523	7.43752367	2.17E-29	6.02E-26
ENSMUSG00000037747	Phyhipl	33	45	8	42	260	223	173	241	2.50831827	8.31E-08	8.37E-06
ENSMUSG00000026413	Pkp1	0	1	20	0	981	266	641	980	6.83494093	2.03E-12	5.38E-10
ENSMUSG00000028751	Pla2g2e	47	27	88	56	199	254	165	275	1.75615342	7.56E-06	0.0004374
ENSMUSG00000041193	Pla2g5	2	3	16	116	273	217	228	247	2.61866682	0.0018575	0.0378228
ENSMUSG00000024973	Plaat5	26	29	28	37	83	115	114	132	1.597611	5.61E-06	0.0003381
ENSMUSG00000051177	Plcb1	650	920	986	805	2293	2270	2330	2111	1.13417399	6.12E-07	4.82E-05
ENSMUSG00000029055	Plch2	9	19	10	5	130	49	36	258	3.16509263	4.06E-06	0.0002565
ENSMUSG00000041757	Plekha6	277	215	269	1135	1440	1201	1751	1501	1.40072096	0.0014757	0.0319985

ENSMUSG00000039713	Plekhg5	1954	2095	1931	2209	5201	3847	7103	6311	1.17571763	1.22E-07	1.16E-05
ENSMUSG00000040852	Plekhh2	503	443	414	483	1598	1372	1369	1402	1.34826177	7.09E-08	7.22E-06
ENSMUSG00000035172	Plekhh3	699	625	860	671	1750	1452	1879	2079	1.04527021	2.80E-05	0.0013324
ENSMUSG00000032374	Plod2	420	491	575	786	1404	1521	1445	1710	1.14705681	8.81E-06	0.0005026
ENSMUSG00000044667	Plppr4	16	16	64	19	306	179	116	259	2.64111176	5.33E-07	4.32E-05
ENSMUSG00000042251	Pm20d1	144	52	92	98	435	285	550	549	1.95245293	6.96E-07	5.41E-05
ENSMUSG00000038400	Pmepa1	3757	4064	10964	3042	12591	5536	23196	22366	1.28798624	0.0026055	0.048793
ENSMUSG00000002012	Pnck	20	25	53	83	230	143	166	98	1.56785536	0.0007828	0.0196818
ENSMUSG00000070802	Pnmal2	49	24	63	177	322	294	324	243	1.67462773	0.0005201	0.0141943
ENSMUSG00000002588	Pon1	128	49	42	45	1108	1011	1133	1126	3.73262126	7.38E-19	5.46E-16
ENSMUSG00000029167	Ppargc1a	258	166	127	272	490	657	606	762	1.31794192	6.12E-05	0.0025421
ENSMUSG00000039457	Ppl	1123	1077	966	1555	3844	6074	4356	4519	1.69816101	1.28E-15	5.93E-13
ENSMUSG00000034613	Ppm1h	544	665	598	902	1522	1620	1889	2104	1.12000549	1.02E-05	0.0005679
ENSMUSG00000035829	Ppp1r26	37	46	29	128	173	196	205	237	1.49789662	0.0007811	0.0196601
ENSMUSG00000052221	Ppp1r36	8	3	3	149	319	205	455	307	2.7885597	0.0024159	0.0461444
ENSMUSG00000021209	Ppp4r4	251	351	190	269	1512	1195	1567	2036	2.28216068	3.49E-13	1.06E-10
ENSMUSG00000028591	Pramef12	12	13	20	24	176	187	288	398	3.65346674	1.45E-16	7.68E-14
ENSMUSG00000039410	Prdm16	317	321	488	456	928	815	1013	2145	1.36841779	9.11E-05	0.0035301
ENSMUSG00000085069	Prdm16os	7	17	83	48	228	225	213	325	2.4337108	1.59E-05	0.000832
ENSMUSG00000041669	Prima1	9	15	19	18	337	151	722	801	4.77851941	4.82E-17	2.73E-14
ENSMUSG00000073600	Prob1	71	88	61	431	741	710	876	888	2.07187037	7.07E-05	0.0028616
ENSMUSG00000070368	Prok1	4	2	3	10	98	172	69	132	4.31295879	9.40E-16	4.50E-13
ENSMUSG00000029086	Prom1	91	133	84	1608	2741	1880	4669	2294	2.39793303	0.0010071	0.0240238
ENSMUSG00000045725	Prr15	20	18	23	34	478	51	89	58	2.58812556	0.0001704	0.0058873
ENSMUSG00000027978	Prss12	102	86	74	70	517	424	540	724	2.43717498	5.43E-13	1.57E-10
ENSMUSG00000028036	Ptgfr	73	29	111	25	272	581	285	352	2.33136309	1.44E-06	0.0001019
ENSMUSG00000032487	Ptgs2	48	53	65	59	12584	62	77	95	5.6015159	2.62E-05	0.0012616
ENSMUSG00000038764	Ptpn3	240	200	362	401	846	853	892	721	1.18879909	2.83E-05	0.001344
ENSMUSG00000042515	Pwwp3b	29	66	62	101	585	225	521	341	2.44492601	2.63E-08	3.00E-06
ENSMUSG00000030674	Qprt	50	59	40	77	153	192	167	139	1.2328391	0.000223	0.0073214
ENSMUSG00000017639	Rab11fip4	95	93	215	674	819	777	1362	847	1.59635433	0.0018271	0.0374109
ENSMUSG00000029923	Rab19	47	28	43	61	120	136	124	84	1.08614403	0.0024634	0.0467778
ENSMUSG00000024511	Rab27b	50	43	81	87	184	144	194	202	1.20711267	0.0005069	0.0139289
ENSMUSG00000020175	Rab36	148	81	101	281	411	378	566	429	1.28258759	0.0009189	0.0223042

ENSMUSG00000038020	Rapgefl1	20	24	29	62	119	111	141	118	1.593658	7.51E-05	0.0030041
ENSMUSG00000027339	Rassf2	1742	1627	1454	2119	4644	7093	4287	4388	1.25441251	1.33E-09	2.11E-07
ENSMUSG00000032940	Rbm11	18	9	9	90	130	119	351	134	2.31016863	0.0006463	0.0168923
ENSMUSG00000043639	Rbm20	30	39	31	70	123	116	150	147	1.38547347	0.0002156	0.007115
ENSMUSG00000038132	Rbm24	14	10	48	64	222	61	160	112	1.80722882	0.0018192	0.037331
ENSMUSG00000024968	Rcor2	258	234	345	215	1423	1026	2035	2019	2.34934768	3.40E-13	1.04E-10
ENSMUSG00000070645	Ren1	0	0	79	29	226	296	247	218	2.95452236	0.0026519	0.0494111
ENSMUSG00000040855	Reps2	497	592	597	887	1878	1780	1879	1583	1.19188304	1.53E-06	0.0001069
ENSMUSG00000030110	Ret	294	253	755	868	3608	1444	6081	4380	2.60687563	2.78E-09	4.00E-07
ENSMUSG00000024186	Rgs11	39	67	52	207	532	468	632	456	2.2698241	8.76E-07	6.63E-05
ENSMUSG00000026360	Rgs2	545	391	694	628	4600	3796	3730	4798	2.63134584	1.57E-28	3.78E-25
ENSMUSG00000039960	Rhou	947	732	509	1403	2429	2610	2685	2147	1.17466867	3.67E-05	0.0016736
ENSMUSG00000040649	Rimklb	30	16	17	108	174	157	326	176	2.04122815	0.0002896	0.0089331
ENSMUSG00000005251	Ripk4	146	185	171	178	456	560	482	497	1.25893435	2.63E-06	0.0001746
ENSMUSG00000068407	Rnase12	11	1	0	5	28	11	555	2	4.78593244	0.0005957	0.0158068
ENSMUSG00000001313	Rnd2	135	123	213	196	528	379	593	399	1.23983636	8.06E-05	0.0031945
ENSMUSG00000010086	Rnf112	53	33	22	48	159	58	166	150	1.4935305	0.0011625	0.0266772
ENSMUSG00000021720	Rnf180	56	54	67	283	539	467	561	502	1.93331873	6.32E-05	0.0026081
ENSMUSG00000048911	Rnf24	978	835	821	652	2285	1557	2062	2693	1.09686563	6.09E-05	0.0025337
ENSMUSG00000032850	Rnft2	126	126	162	187	539	389	434	455	1.32365992	6.06E-06	0.0003588
ENSMUSG00000051169	Rpusd3	80	75	88	116	238	264	212	278	1.18411215	8.44E-05	0.0033162
ENSMUSG00000019880	Rspo3	64	76	59	84	423	289	371	231	1.92976703	1.25E-08	1.55E-06
ENSMUSG00000056054	S100a8	11	23	33	45	105	71	127	60	1.43714474	0.0017479	0.036202
ENSMUSG00000033208	S100b	17	10	70	35	161	131	220	232	2.24498294	1.59E-05	0.0008306
ENSMUSG00000025203	Scd2	2414	1930	3403	4585	9370	4578	10849	7009	1.11533711	0.0001059	0.0039752
ENSMUSG00000022123	Scel	57	39	39	32	508	382	646	547	3.34253234	1.16E-19	9.58E-17
ENSMUSG00000044770	Scml4	37	48	51	134	163	305	221	186	1.422953	0.0006638	0.0172424
ENSMUSG00000070304	Scn2b	54	44	46	80	130	178	130	127	1.04423734	0.0018447	0.0375888
ENSMUSG00000046480	Scn4b	84	101	99	127	808	990	848	818	2.78612462	8.11E-24	1.05E-20
ENSMUSG00000023033	Scn8a	29	21	24	88	109	145	196	136	1.59406885	0.0006333	0.0166146
ENSMUSG00000003585	Sec14l2	24	28	44	82	448	92	133	98	1.8851689	0.001074	0.0252717
ENSMUSG00000019368	Sec14l4	32	50	31	381	818	787	971	866	2.5837185	6.97E-05	0.0028273
ENSMUSG00000068877	Selenbp2	9	7	11	23	78	69	250	95	3.02949287	6.54E-08	6.69E-06
ENSMUSG00000050379	Septin6	528	543	447	551	1422	1347	1313	1418	1.11851323	5.46E-06	0.0003317

ENSMUSG00000071179	Serpina16	0	6	0	1	88	20	351	44	5.78316115	7.04E-08	7.18E-06
ENSMUSG00000071178	Serpina1b	13	14	15	13	97	112	95	119	2.64218769	3.08E-12	7.70E-10
ENSMUSG00000021081	Serpina1f	19	24	0	4	8307	1	3162	4	7.61082581	5.65E-05	0.0023726
ENSMUSG00000079012	Serpina3m	64	33	13	75	411	191	331	349	2.5172261	4.75E-07	3.88E-05
ENSMUSG00000016262	Sertad4	169	140	183	220	493	468	1040	898	1.75338589	4.71E-07	3.87E-05
ENSMUSG00000061186	Sfmbt2	19	36	40	51	133	133	195	139	1.76537625	2.94E-06	0.000193
ENSMUSG00000035296	Sgcg	1	4	3	4	112	505	145	222	5.98405461	1.45E-20	1.34E-17
ENSMUSG00000037833	Sh2d4b	36	69	82	89	169	215	147	207	1.14082452	0.0011616	0.026667
ENSMUSG00000040666	Sh3bgr	29	22	88	63	547	115	178	134	2.03704827	0.0004187	0.0119814
ENSMUSG00000044461	Shisa2	60	142	162	301	349	339	881	562	1.43362093	0.0015827	0.0337245
ENSMUSG00000041889	Shisa4	87	66	76	117	331	267	306	400	1.6394803	4.73E-07	3.88E-05
ENSMUSG00000062760	Shisal1	6	15	13	7	279	152	311	295	4.36841664	2.06E-20	1.81E-17
ENSMUSG00000022899	Slc15a2	340	408	477	276	1298	1519	1126	1236	1.48659684	7.34E-08	7.44E-06
ENSMUSG00000032902	Slc16a1	1517	2064	1405	2423	6534	8450	4424	8309	1.61325719	2.25E-11	4.88E-09
ENSMUSG00000025094	Slc18a2	69	69	73	111	224	273	272	189	1.28664867	4.07E-05	0.0018202
ENSMUSG00000020142	Slc1a4	254	158	328	201	596	543	1236	845	1.49483793	2.75E-05	0.0013115
ENSMUSG00000040966	Slc22a2	22	22	34	17	82	117	58	93	1.57715363	8.28E-05	0.0032669
ENSMUSG00000021265	Slc25a29	194	208	145	207	614	311	761	430	1.20631981	0.0006279	0.0165202
ENSMUSG00000040740	Slc25a34	9	39	14	61	267	534	131	207	2.91320529	5.47E-07	4.40E-05
ENSMUSG00000021509	Slc25a48	17	13	11	22	118	86	211	136	2.84389926	1.93E-10	3.68E-08
ENSMUSG00000036196	Slc26a8	31	34	27	39	89	145	121	114	1.53879052	9.27E-06	0.0005218
ENSMUSG00000036298	Slc2a13	480	510	380	403	1645	941	1495	2669	1.64633746	1.35E-06	9.66E-05
ENSMUSG00000042195	Slc35f2	9	21	2	84	234	80	318	88	2.41015736	0.0021469	0.0421974
ENSMUSG00000010064	Slc38a3	52	28	101	203	1593	494	741	1928	3.41746805	2.46E-09	3.63E-07
ENSMUSG00000010122	Slc47a1	19	29	11	54	158	106	170	125	2.03854243	1.29E-05	0.000695
ENSMUSG00000068323	Slc4a5	2	3	1	1	182	45	132	118	5.72925782	2.75E-17	1.59E-14
ENSMUSG00000027463	Slc52a3	140	143	201	404	953	726	1153	1277	1.96294779	8.48E-08	8.46E-06
ENSMUSG00000021565	Slc6a19	5	3	1	45	95	156	169	109	3.04865612	8.64E-05	0.0033814
ENSMUSG00000055368	Slc6a2	56	15	29	68	1011	647	1284	935	4.25728747	1.94E-20	1.73E-17
ENSMUSG00000028542	Slc6a9	230	182	211	422	802	564	1162	1169	1.56472844	9.37E-06	0.000526
ENSMUSG00000031596	Slc7a2	245	335	604	653	1622	2109	2273	1445	1.75108208	2.47E-08	2.82E-06
ENSMUSG00000022756	Slc7a4	62	66	56	200	442	311	529	357	1.84327937	1.77E-05	0.0009138
ENSMUSG00000040010	Slc7a5	265	217	323	710	1535	1409	1833	1180	1.7189246	7.93E-07	6.08E-05
ENSMUSG00000031558	Slit2	184	212	560	482	1075	1118	975	916	1.24583226	0.0003098	0.0094098

ENSMUSG00000033214	Slitrk5	21	28	14	27	206	268	237	135	2.92274326	1.35E-13	4.46E-11
ENSMUSG00000038059	Smim3	98	73	88	175	331	293	413	284	1.33576581	0.0001077	0.0040334
ENSMUSG00000045667	Smtnl2	96	93	188	238	1186	546	1861	2245	3.00850121	4.09E-11	8.55E-09
ENSMUSG00000047793	Sned1	1463	1114	940	1781	5425	4594	4241	3699	1.47496874	3.97E-10	6.97E-08
ENSMUSG00000049313	Sorl1	763	682	717	1025	2005	2659	1878	1913	1.11597735	1.71E-06	0.0001186
ENSMUSG00000001494	Sost	0	0	7	2	933	3	11	6	6.47282095	0.0001492	0.0052646
ENSMUSG00000000567	Sox9	307	216	128	771	1452	1185	1677	1509	1.7801473	4.12E-05	0.0018383
ENSMUSG00000024352	Spata24	14	24	27	45	80	101	108	80	1.47002662	0.0002125	0.0070515
ENSMUSG00000074476	Spc24	31	12	64	29	134	114	84	111	1.42937629	0.0018427	0.0375763
ENSMUSG00000061878	Sphk1	93	110	148	397	698	542	598	478	1.38670745	0.0008189	0.0204046
ENSMUSG00000050074	Spink8	71	17	1	14	3100	88	3823	62	5.77496841	3.19E-06	0.0002073
ENSMUSG00000074227	Spint2	631	644	714	2182	2860	2051	3689	2355	1.14946005	0.0014079	0.0308406
ENSMUSG00000038156	Spon1	1209	1387	1210	1820	5722	4623	7005	5441	1.74127873	3.65E-16	1.84E-13
ENSMUSG00000067889	Sptbn2	56	56	34	59	707	115	104	74	2.02010616	0.0019693	0.039533
ENSMUSG00000074899	Sptbn5	108	150	87	144	544	498	362	486	1.65830143	1.94E-07	1.76E-05
ENSMUSG00000112449	Srp54b	15	40	32	3	131	180	80	61	1.99172385	0.0010884	0.0255358
ENSMUSG00000031995	St14	55	52	77	251	541	327	451	238	1.60366777	0.0010188	0.0241677
ENSMUSG00000022885	St6gal1	813	886	1288	1122	2081	3527	3918	2075	1.2059751	2.60E-06	0.0001733
ENSMUSG00000035459	Stab2	30	74	54	124	145	608	180	185	1.67745171	0.0009675	0.0232188
ENSMUSG00000039954	Stk32a	3	4	26	23	532	116	1189	608	5.21962864	6.70E-12	1.58E-09
ENSMUSG00000036863	Syde2	784	955	872	658	1591	1660	2390	2565	1.03518983	0.0001272	0.0046111
ENSMUSG00000009394	Syn2	215	364	272	253	1328	807	1451	1923	2.04012801	1.67E-09	2.56E-07
ENSMUSG00000016200	Syt14	83	109	101	84	236	246	386	358	1.41182362	1.86E-05	0.0009562
ENSMUSG00000026452	Syt2	45	78	74	129	527	180	746	686	2.46763805	1.34E-07	1.27E-05
ENSMUSG00000000782	Tcf7	193	203	181	453	433	1380	1030	442	1.37560295	0.0009037	0.0219896
ENSMUSG00000052415	Tchh	50	36	25	80	316	676	396	468	2.98146685	2.79E-13	8.71E-11
ENSMUSG00000062859	Tcp11	15	8	6	50	71	48	164	93	2.00650028	0.0014019	0.0307588
ENSMUSG00000028011	Tdo2	72	165	58	143	423	422	352	293	1.47070466	0.0001327	0.0047796
ENSMUSG00000045968	Teddm2	50	67	28	66	121	237	162	119	1.28332954	0.0011214	0.0259582
ENSMUSG00000046694	Tent5b	124	149	171	231	439	636	484	857	1.56377862	1.40E-06	9.93E-05
ENSMUSG00000029359	Tesc	50	47	58	139	224	166	391	220	1.51485001	0.000437	0.0123642
ENSMUSG00000029664	Tfpi2	60	79	143	106	223	189	293	304	1.11388367	0.0017518	0.0362569
ENSMUSG00000048503	Tlcd5	203	175	170	245	474	592	574	495	1.1369799	3.07E-05	0.0014396
ENSMUSG00000062545	Tlr12	13	13	7	98	93	176	184	164	1.99552197	0.0025795	0.0484358

ENSMUSG00000056498	Tmem154	294	307	251	383	592	1182	577	789	1.04088823	0.0005217	0.0142188
ENSMUSG00000024245	Tmem178	45	39	42	51	147	115	193	227	1.66731296	4.84E-06	0.0002978
ENSMUSG00000055692	Tmem191c	104	127	96	134	331	358	324	261	1.17189407	6.81E-05	0.0027735
ENSMUSG00000034435	Tmem30b	27	26	31	228	714	278	615	337	2.43149356	0.0001638	0.0057016
ENSMUSG00000023153	Tmem52	6	9	5	89	203	86	360	328	2.96397077	0.0001869	0.0063232
ENSMUSG00000032268	Tmprss5	25	11	53	33	96	112	64	139	1.48072375	0.0014397	0.0313889
ENSMUSG00000024793	Tnfrsf25	223	270	214	346	599	563	1068	717	1.20608216	0.0001207	0.0044433
ENSMUSG00000050395	Tnfsf15	15	28	36	53	152	152	106	164	1.85326811	4.58E-06	0.0002849
ENSMUSG00000027692	Tnik	93	132	121	156	372	428	399	254	1.24370772	4.96E-05	0.0021371
ENSMUSG00000031250	Tnmd	1	1	0	5	137	55	200	144	5.91587345	1.73E-17	1.02E-14
ENSMUSG00000064179	Tnnt1	25	26	34	67	132	100	186	133	1.59886621	9.67E-05	0.0036994
ENSMUSG00000048546	Tob2	1797	2332	2401	2398	5809	7490	9327	9337	1.55514595	3.41E-14	1.30E-11
ENSMUSG00000041272	Tox	60	37	74	86	184	322	203	173	1.48611458	3.23E-05	0.0015044
ENSMUSG00000000296	Tpd52l1	36	42	15	104	311	146	377	219	2.16544601	3.51E-05	0.0016158
ENSMUSG00000024173	Tpsab1	100	109	38	247	367	1577	1113	540	2.5603896	1.20E-06	8.66E-05
ENSMUSG00000033825	Tpsb2	490	479	552	937	2222	3216	2841	1647	1.72935344	6.99E-10	1.18E-07
ENSMUSG00000019787	Trdn	2	8	18	2	134	263	193	176	4.35754839	1.79E-14	7.05E-12
ENSMUSG00000031026	Trim66	20	34	31	50	78	273	87	93	1.66054549	0.0004313	0.0122543
ENSMUSG00000040350	Trim7	89	76	92	298	400	491	538	373	1.4422296	0.0005639	0.0151385
ENSMUSG00000022510	Trp63	43	39	38	90	532	405	581	496	2.99484247	5.27E-16	2.59E-13
ENSMUSG00000052387	Trpm3	54	105	77	264	911	978	1407	917	2.82091632	7.52E-11	1.52E-08
ENSMUSG00000005952	Trpv1	146	88	89	85	398	375	238	446	1.53303787	1.61E-05	0.0008431
ENSMUSG00000049580	Tsku	301	167	341	299	903	725	917	757	1.29705701	1.20E-05	0.0006561
ENSMUSG00000038984	Tspyl5	64	65	65	80	159	170	201	170	1.06548969	0.0004711	0.0131755
ENSMUSG00000006784	Ttc25	16	19	17	127	187	139	278	216	1.97353262	0.0012446	0.0280622
ENSMUSG00000085873	Ttc39aos1	13	31	18	62	148	107	156	129	1.86439655	6.02E-05	0.002506
ENSMUSG00000030276	Ttll3	140	134	81	338	700	930	903	789	1.9885614	2.88E-07	2.48E-05
ENSMUSG00000030137	Tuba8	52	18	14	21	206	164	96	180	2.30423518	2.63E-06	0.0001746
ENSMUSG00000045007	Tubg2	48	27	52	45	130	192	187	173	1.69362725	1.12E-06	8.21E-05
ENSMUSG00000022615	Tymp	31	32	35	105	126	131	222	206	1.50326017	0.0008567	0.0210979
ENSMUSG00000067049	Unc93a	22	30	4	16	82	138	100	87	2.16345662	1.99E-05	0.0010046
ENSMUSG00000033427	Upb1	21	35	29	71	87	131	114	115	1.24441345	0.0021771	0.04268
ENSMUSG00000032010	Usp2	272	242	343	698	984	1172	1493	1350	1.42572816	2.03E-05	0.0010211
ENSMUSG00000037568	Vash2	30	28	39	21	449	42	128	43	2.22581472	0.0012562	0.0282074

ENSMUSG00000011171	Vipr2	42	28	34	25	205	159	159	284	2.35207959	1.97E-09	2.98E-07
ENSMUSG00000024076	Vit	601	413	735	716	1165	1972	1730	1438	1.06433463	6.97E-05	0.0028273
ENSMUSG00000050122	Vwa3b	13	4	8	72	104	102	206	114	2.20895972	0.0012696	0.0283829
ENSMUSG00000029636	Wasf3	114	110	148	190	346	320	423	290	1.02068411	0.0008051	0.0201316
ENSMUSG00000039099	Wdr93	207	176	282	230	1005	403	957	841	1.57659372	5.60E-06	0.0003381
ENSMUSG00000070529	Wfdc10	14	2	2	4	27	1	538	2	4.34642673	0.0021812	0.0427353
ENSMUSG00000067704	Wfdc13	2	5	0	3	2	1	550	2	5.43470284	0.0012076	0.0274267
ENSMUSG00000070533	Wfdc8	28	7	3	81	68	60	1068	103	3.20777827	0.0012667	0.0283631
ENSMUSG00000041245	Wnk3	32	36	44	45	84	155	89	90	1.11148493	0.0016079	0.0340345
ENSMUSG00000035112	Wnk4	117	93	56	253	566	560	671	430	1.83062482	9.58E-06	0.000536
ENSMUSG00000074987	Wt1os	86	62	42	69	229	233	234	239	1.55036189	5.51E-06	0.000334
ENSMUSG00000015342	Xk	69	48	83	116	439	235	195	175	1.46082376	0.0003129	0.009482
ENSMUSG00000045064	Zc2hc1c	57	46	59	169	260	208	403	302	1.57698131	0.000275	0.0086117
ENSMUSG00000039981	Zc3h12d	110	68	117	101	351	536	300	319	1.62488291	5.26E-07	4.27E-05
ENSMUSG00000031428	Zcchc18	22	27	27	70	104	146	99	93	1.32250254	0.001494	0.0323196
ENSMUSG00000057895	Zfp105	74	94	94	115	218	278	273	291	1.20616711	5.21E-05	0.0022255
ENSMUSG00000031351	Zfp185	1162	920	817	1993	3266	1604	4015	3149	1.04016275	0.0011862	0.0270407
ENSMUSG00000047342	Zfp286	25	24	46	56	92	127	123	106	1.29366243	0.0005323	0.0144722
ENSMUSG00000043903	Zfp469	24	39	83	44	171	165	212	220	1.74567598	1.27E-05	0.0006866
ENSMUSG00000044676	Zfp612	231	207	162	334	556	861	709	668	1.28797862	1.57E-05	0.0008223
ENSMUSG00000111063	Zfp660	39	32	34	64	113	227	195	162	1.75091953	2.16E-06	0.0001478
ENSMUSG00000032425	Zfp949	382	380	805	409	1733	2663	2439	1648	1.81103509	1.52E-09	2.36E-07
ENSMUSG00000038630	Zkscan16	9	15	18	27	153	89	134	75	2.43874692	4.46E-08	4.76E-06
ENSMUSG00000036086	Zranb3	65	93	149	131	284	465	302	277	1.31339475	9.16E-05	0.0035437
Down_DEGs												
ENSMUSG00000031214	Ophn1	2150	2368	1903	2321	1211	1791	1126	1267	-1.0009976	5.69E-06	0.0003413
ENSMUSG00000043644	0610009L18 Rik	107	111	98	118	66	64	81	50	-1.0242817	0.0017121	0.0356347
ENSMUSG00000097616	1110019D14 Rik	147	125	171	128	73	72	72	82	-1.2209951	0.000106	0.0039764
ENSMUSG00000054676	1600014C10Rik	3099	3283	3277	2543	1013	1644	864	1234	-1.669519	1.60E-12	4.31E-10
ENSMUSG00000027327	1700037H04Rik	2653	2644	2935	2576	726	915	623	934	-2.0517463	6.07E-20	5.18E-17
ENSMUSG00000099349	1700047G03Rik	243	182	1289	159	88	65	42	101	-2.9137944	3.46E-06	0.0002224
ENSMUSG00000070997	1700055D18 Rik	107	103	109	82	50	47	44	55	-1.3253351	9.13E-05	0.0035336
ENSMUSG00000044854	1700056E22Rik	147	118	126	86	66	61	50	84	-1.1671872	0.0012373	0.0279594
ENSMUSG00000101856	1700096K18 Rik	430	383	335	314	151	198	243	278	-1.0484363	0.0009708	0.0232589

ENSMUSG00000100594	2810414N06 Rik	211	197	198	144	86	124	88	125	-1.1313106	0.0005258	0.0143163
ENSMUSG00000053714	4732471J01 Rik	348	310	266	256	132	121	85	123	-1.6556287	2.27E-07	2.01E-05
ENSMUSG00000097073	9430037G07 Rik	84	69	55	81	38	37	32	14	-1.5535964	0.0001464	0.0051831
ENSMUSG00000097462	9530026P05 Rik	276	320	323	337	95	123	127	117	-1.7325433	1.28E-09	2.05E-07
ENSMUSG00000006462	A530013C23Rik	82	83	109	77	12	18	12	10	-3.0407343	4.20E-14	1.57E-11
ENSMUSG00000043122	A530016L24Rik	5638	4112	5983	3943	2915	1337	3140	4108	-1.0493412	0.000483	0.0134407
ENSMUSG00000046764	A530053G22 Rik	571	791	874	778	391	251	299	371	-1.47315	3.34E-07	2.83E-05
ENSMUSG00000054181	A930012O16 Rik	115	102	93	84	54	67	70	47	-1.0317541	0.0024326	0.0463509
ENSMUSG00000055782	Abcd2	20387	24314	22767	18993	6454	3558	5549	7237	-2.2027428	1.95E-19	1.54E-16
ENSMUSG00000000686	Abhd15	887	1315	998	782	601	579	384	763	-1.0718259	0.0004918	0.0136392
ENSMUSG00000032540	Abhd5	3384	5252	3166	4438	2267	2263	1508	2230	-1.2682657	3.01E-08	3.35E-06
ENSMUSG00000032735	Ablim3	3708	4159	3738	3375	1810	2401	1462	1996	-1.2699955	7.53E-10	1.27E-07
ENSMUSG00000010651	Acaa1b	2145	2357	2802	1592	864	1718	632	901	-1.4317972	1.69E-06	0.0001172
ENSMUSG00000036880	Acaa2	2918	3633	4361	3683	2031	2400	1896	1991	-1.1005274	5.95E-09	7.97E-07
ENSMUSG00000020532	Acaca	12445	20369	7510	9413	2675	2413	2519	3544	-2.4655874	6.41E-16	3.12E-13
ENSMUSG00000042010	Acacb	4831	13311	4181	8817	1412	4884	1288	2331	-1.9846883	1.06E-06	7.81E-05
ENSMUSG00000090150	Acad11	3892	5131	5756	3079	1075	872	1080	1239	-2.3541057	6.05E-21	5.89E-18
ENSMUSG00000029545	Acads	4287	4011	3740	3381	2297	2135	1954	2736	-1.0522845	3.56E-07	3.00E-05
ENSMUSG00000056938	Acbd4	5197	4387	4270	3002	2282	2734	1861	2270	-1.1934644	1.07E-07	1.04E-05
ENSMUSG00000052392	Acot4	439	567	589	522	104	185	174	169	-2.039251	6.54E-12	1.55E-09
ENSMUSG00000001348	Acp5	8020	12006	9417	9177	2417	3464	1521	3151	-2.1740727	2.09E-18	1.45E-15
ENSMUSG00000018796	Acsl1	31732	41026	42865	24303	24062	10552	19822	27458	-1.0504619	0.0002808	0.0087554
ENSMUSG00000027605	Acss2	1499	1324	1436	1140	735	588	637	800	-1.2586202	1.45E-06	0.0001027
ENSMUSG00000035948	Acss3	2163	3356	1926	2373	1087	2003	1061	1146	-1.2083432	5.39E-06	0.0003278
ENSMUSG00000026834	Acvr1c	7709	10390	5408	7085	3583	3291	1980	4530	-1.4921076	5.74E-08	5.94E-06
ENSMUSG00000022894	Adamts5	10272	8045	13549	10758	6192	4460	4712	5628	-1.2971442	3.02E-10	5.47E-08
ENSMUSG00000020709	Adap2	810	1997	2177	1069	640	1244	782	873	-1.0781319	0.0011095	0.0258443
ENSMUSG00000056492	Adgrf5	9513	14375	13385	10987	6113	11068	4824	7671	-1.0094592	1.55E-05	0.0008156
ENSMUSG00000039167	Adgrl4	2892	3685	3537	2870	1412	2503	882	1486	-1.3604562	3.75E-07	3.13E-05
ENSMUSG00000025911	Adhfe1	5895	6081	5369	4811	2205	2371	2018	2907	-1.5192501	9.32E-14	3.19E-11
ENSMUSG00000044405	Adig	3599	4553	3688	3960	489	731	493	553	-3.1050701	2.91E-45	1.79E-41
ENSMUSG00000022878	Adipoq	103365	106964	105996	70598	12666	11465	15594	19300	-3.0059506	8.95E-35	3.31E-31
ENSMUSG00000030790	Adm	248	271	359	634	155	295	123	196	-1.254372	0.0006978	0.0179995
ENSMUSG00000020178	Adora2a	1003	1078	706	893	410	661	215	420	-1.4268543	2.29E-05	0.0011264

ENSMUSG00000045875	Adra1a	364	299	304	234	200	148	150	202	-1.0730076	0.0007661	0.0193575
ENSMUSG00000027335	Adra1d	345	378	489	389	262	210	138	221	-1.2277153	6.51E-05	0.0026752
ENSMUSG00000031489	Adrb3	38246	23132	19540	26635	3039	3059	937	2220	-3.8485382	4.29E-28	9.16E-25
ENSMUSG00000050103	Agmo	1631	1664	1816	1448	584	899	704	585	-1.5482347	2.13E-10	3.99E-08
ENSMUSG00000031980	Agt	12090	15063	6472	13480	3895	3967	4465	4173	-1.8123434	2.23E-13	7.08E-11
ENSMUSG00000029772	Ahcyl2	5333	6707	5277	5387	2623	2980	2140	3243	-1.3445435	2.87E-11	6.06E-09
ENSMUSG00000020085	Aifm2	7097	7370	7441	5874	4109	3545	2840	4377	-1.1938508	7.57E-09	9.91E-07
ENSMUSG00000028792	Ak2	7382	8229	8608	7257	2460	3155	2474	2823	-1.8258769	3.03E-24	4.32E-21
ENSMUSG00000075296	Aldh3b2	408	422	1052	505	243	127	223	360	-1.5784284	0.0001013	0.0038407
ENSMUSG00000004105	Angptl2	3928	5004	6422	4531	2033	2400	2319	3709	-1.2083042	1.97E-07	1.78E-05
ENSMUSG00000002289	Angptl4	17608	19419	14345	14585	6214	13158	1686	6291	-1.6019025	4.01E-05	0.0017981
ENSMUSG00000074771	Ankef1	5289	5752	4695	4229	398	398	348	530	-3.8733593	5.70E-57	7.90E-53
ENSMUSG00000024659	Anxa1	5993	5026	12127	7145	3480	3827	4054	3370	-1.3169052	2.13E-08	2.49E-06
ENSMUSG00000019326	Aoc3	55119	52666	60212	41740	6510	4852	5524	7445	-3.395195	1.38E-49	1.10E-45
ENSMUSG00000024480	Ap3s1	3337	3176	4521	2757	1787	1884	2124	1810	-1.1503789	4.55E-08	4.83E-06
ENSMUSG00000033096	Apmap	5246	5484	5289	4613	2139	1733	1803	2160	-1.6870951	2.15E-17	1.25E-14
ENSMUSG00000033576	Apol6	12745	15721	15849	12294	3833	3950	4986	5832	-1.8940948	2.83E-19	2.18E-16
ENSMUSG00000022892	App	23658	30969	36321	27574	17837	17948	14496	17280	-1.0984274	2.64E-09	3.86E-07
ENSMUSG00000028427	Aqp7	4026	5420	4298	3986	1070	1909	609	1371	-2.1517201	7.44E-14	2.61E-11
ENSMUSG00000019987	Arg1	26	42	430	91	19	29	24	35	-2.7012936	0.0001771	0.0060824
ENSMUSG00000034842	Art3	8311	7061	9001	6355	2003	2394	2517	2179	-2.0548129	7.81E-26	1.31E-22
ENSMUSG00000048355	Arxes1	827	787	1149	925	233	232	195	308	-2.2095785	9.12E-15	3.72E-12
ENSMUSG00000048040	Arxes2	3264	4044	3602	4183	632	722	590	726	-2.7886355	4.19E-41	2.11E-37
ENSMUSG00000029752	Asns	2419	2739	2103	2296	1143	1025	1072	1517	-1.2975247	4.29E-08	4.62E-06
ENSMUSG00000038539	Atf5	3234	2804	3274	3457	1562	1745	1220	1330	-1.4176797	8.87E-13	2.46E-10
ENSMUSG00000007097	Atp1a2	23225	25817	19778	16203	13333	10543	12341	14160	-1.0532586	1.14E-06	8.31E-05
ENSMUSG00000027546	Atp9a	5815	5868	8069	6137	2211	2210	2228	2041	-1.8615434	5.10E-24	6.91E-21
ENSMUSG00000060802	B2m	69880	67885	58886	43913	32060	35192	33364	28749	-1.2042191	3.44E-09	4.85E-07
ENSMUSG00000033849	B3galt2	12305	12409	6739	8636	1251	585	1125	1822	-3.3558433	3.02E-22	3.49E-19
ENSMUSG00000084960	B430010123Rik	647	476	401	270	58	48	38	62	-3.4296584	1.70E-17	1.01E-14
ENSMUSG00000086432	B430119L08 Rik	519	518	520	365	339	106	205	253	-1.366917	0.0003545	0.0104405
ENSMUSG00000085211	B430219N15Rik	256	262	280	160	52	37	47	59	-2.5862767	2.39E-12	6.22E-10
ENSMUSG00000043740	B430306N03Rik	50	54	86	97	42	41	27	37	-1.2342739	0.0010402	0.0245912
ENSMUSG00000028416	Bag1	5572	5706	6121	4445	2280	2304	2484	2589	-1.4725897	3.44E-14	1.30E-11

ENSMUSG00000002083	Bbc3	521	303	380	332	189	257	161	177	-1.2803552	4.81E-05	0.0020799
ENSMUSG00000092564	BC051226	324	264	251	297	161	182	173	163	-1.0391337	0.000234	0.0076106
ENSMUSG00000002015	Bcap31	6228	6523	8787	6093	2917	2645	3239	2698	-1.5506615	1.46E-15	6.70E-13
ENSMUSG00000000317	Bcl6b	1034	824	912	872	549	394	541	681	-1.0341159	0.0002597	0.008236
ENSMUSG00000001999	Blvra	542	817	1212	783	448	503	483	413	-1.1431625	6.89E-05	0.0028007
ENSMUSG00000027358	Bmp2	305	573	345	371	227	295	215	225	-1.0332858	0.0008799	0.0215639
ENSMUSG00000078566	Bnip3	8447	12698	11472	9760	5448	4791	5216	8186	-1.1249819	5.54E-07	4.44E-05
ENSMUSG00000071657	Bscl2	7636	7529	5049	6433	3579	3030	2652	3296	-1.383994	1.08E-10	2.17E-08
ENSMUSG00000040283	Btnl9	3778	6219	4760	3901	1524	3399	699	2207	-1.5741597	5.48E-06	0.0003322
ENSMUSG00000116138	C030006K11Rik	1432	1576	1362	1387	761	730	723	770	-1.2417796	1.41E-07	1.33E-05
ENSMUSG00000023571	C1qtnf12	1622	1558	1500	1726	1078	531	516	575	-1.5244972	3.30E-07	2.80E-05
ENSMUSG00000071347	C1qtnf9	1922	1752	2205	1599	866	933	659	950	-1.4280734	3.89E-09	5.42E-07
ENSMUSG00000024371	C2	1763	2822	1274	2241	979	1065	752	991	-1.3973588	5.82E-07	4.64E-05
ENSMUSG00000022181	C6	3765	4465	1276	2205	834	629	661	635	-2.4026663	2.10E-11	4.59E-09
ENSMUSG00000097574	C920006O11 Rik	315	349	258	288	118	203	164	133	-1.2800248	2.84E-05	0.0013447
ENSMUSG00000069806	Cacng7	573	505	597	632	166	148	150	181	-2.1208165	1.67E-14	6.68E-12
ENSMUSG00000051146	Camk2n2	194	132	141	109	54	61	47	61	-1.6734995	2.42E-06	0.0001634
ENSMUSG00000027559	Car3	594578	551399	549568	400742	191848	110837	153247	240723	-1.8750641	5.45E-13	1.57E-10
ENSMUSG00000000805	Car4	1487	2220	1623	1743	731	1690	412	904	-1.2420287	0.0002971	0.009103
ENSMUSG00000031373	Car5b	7744	9354	6616	7272	2812	2253	2556	2954	-1.8435958	9.74E-19	7.11E-16
ENSMUSG00000031883	Car7	236	276	310	247	167	154	141	177	-1.0288578	0.0003806	0.0110384
ENSMUSG00000007655	Cav1	78911	79646	100089	64471	40833	22907	37519	47145	-1.400318	9.80E-09	1.24E-06
ENSMUSG00000000058	Cav2	11945	15939	19839	12500	6826	4672	7013	7730	-1.4756472	1.24E-10	2.48E-08
ENSMUSG00000045954	Cavin2	19479	22367	26998	15924	7249	8501	6022	8021	-1.8062366	4.50E-18	2.84E-15
ENSMUSG00000037060	Cavin3	6493	4096	7970	4607	2730	2156	1653	2485	-1.6453348	4.37E-11	9.12E-09
ENSMUSG00000022665	Ccdc80	46024	41077	41339	43809	15051	14557	12421	18565	-1.7968426	5.66E-21	5.61E-18
ENSMUSG00000004814	Ccl24	315	454	520	466	185	127	198	218	-1.5398686	9.71E-07	7.25E-05
ENSMUSG00000018927	Ccl6	3618	3838	7079	6358	1920	4554	2009	2934	-1.1629794	1.59E-05	0.0008327
ENSMUSG00000000184	Ccnd2	5864	6548	9503	5294	3510	4514	3134	3606	-1.1779936	2.15E-08	2.51E-06
ENSMUSG00000025804	Ccr1	364	526	511	470	201	386	191	251	-1.1671199	0.000129	0.0046668
ENSMUSG00000025510	Cd151	8475	8977	11946	9117	5689	5378	5040	5598	-1.1121491	5.41E-10	9.35E-08
ENSMUSG00000028076	Cd1d1	7786	5765	10021	4231	2015	1948	882	1087	-2.5324011	9.67E-16	4.58E-13
ENSMUSG00000051906	Cd209f	734	1310	1083	2217	658	575	932	571	-1.2283707	0.0001799	0.0061535
ENSMUSG00000079168	Cd209g	414	795	651	1072	390	280	398	357	-1.305199	4.37E-05	0.0019218

ENSMUSG00000017309	Cd300lg	7406	10146	8889	7373	3301	6626	2298	4553	-1.3257236	5.44E-07	4.39E-05
ENSMUSG00000060703	Cd302	2754	3899	3651	3032	1702	2239	2279	1739	-1.0430374	3.69E-07	3.09E-05
ENSMUSG00000002944	Cd36	103563	135601	145982	104000	51036	67070	46247	59177	-1.4278546	1.10E-13	3.71E-11
ENSMUSG00000032679	Cd59a	2809	3070	6303	2818	1627	1729	1388	1902	-1.4545179	4.36E-08	4.68E-06
ENSMUSG00000068686	Cd59b	122	80	144	98	53	52	80	49	-1.2102597	0.0007969	0.0199701
ENSMUSG00000031871	Cdh5	10486	12215	10265	9617	5868	7775	4147	7065	-1.0809241	3.31E-07	2.81E-05
ENSMUSG00000039496	Cdnf	180	180	226	147	85	147	84	133	-1.0093317	0.0024908	0.0471704
ENSMUSG00000033022	Cdo1	38735	40392	27858	29595	16633	9170	15834	13642	-1.5965568	6.40E-11	1.31E-08
ENSMUSG00000030878	Cdr2	1993	1884	2808	1257	996	744	678	1143	-1.4433071	1.44E-06	0.000102
ENSMUSG00000039518	Cdsn	3239	1410	4171	2064	573	378	272	651	-2.8130119	4.67E-14	1.73E-11
ENSMUSG00000056501	Cebpb	984	367	483	134	76	121	179	178	-2.157279	4.45E-05	0.0019511
ENSMUSG00000018509	Cenpv	682	566	523	562	242	364	194	231	-1.489117	3.67E-07	3.08E-05
ENSMUSG00000008206	Cers4	1819	1809	2219	1853	943	1053	910	1068	-1.2443304	1.07E-08	1.34E-06
ENSMUSG00000056973	Ces1d	43374	45779	34763	32204	6073	8094	7361	6250	-2.8017965	4.01E-41	2.11E-37
ENSMUSG00000031725	Ces1f	7506	5240	2623	3734	738	1874	521	758	-2.6480075	1.02E-11	2.28E-09
ENSMUSG00000061780	Cfd	317373	286105	148436	286390	18139	20729	17818	20896	-4.0465793	4.59E-55	4.24E-51
ENSMUSG00000078185	Chml	601	714	884	706	381	530	354	466	-1.0394308	8.47E-05	0.0033226
ENSMUSG00000014077	Chp1	13694	15469	17656	13605	5319	6929	4914	5822	-1.691923	1.79E-20	1.63E-17
ENSMUSG00000060002	Chpt1	26599	27383	27815	19337	15884	14765	12974	14507	-1.0976969	1.88E-08	2.28E-06
ENSMUSG00000031283	Chrdl1	8657	13640	11453	9699	4603	5195	4591	5259	-1.4402751	1.88E-13	6.04E-11
ENSMUSG00000027221	Chst1	8868	8943	6781	6230	2514	2843	1585	3389	-1.8824004	1.09E-13	3.71E-11
ENSMUSG00000036599	Chst12	2566	1867	2667	2201	1542	1472	978	1206	-1.1321339	1.20E-06	8.66E-05
ENSMUSG00000035930	Chst4	313	116	106	237	70	76	66	133	-1.4544312	0.0011183	0.0259084
ENSMUSG00000037493	Cib2	2588	3197	4155	2446	931	677	808	821	-2.2184054	2.41E-19	1.88E-16
ENSMUSG00000030278	Cidec	63530	92378	96151	58856	14781	11944	11716	21042	-2.6701557	6.48E-25	9.98E-22
ENSMUSG00000032578	Cish	1386	255	853	4115	757	792	158	202	-2.0488671	0.0016111	0.0340559
ENSMUSG00000036960	Clca2	229	191	130	103	40	36	42	49	-2.2750949	1.86E-08	2.26E-06
ENSMUSG00000041378	Cldn5	5211	5193	3508	5689	2727	3060	1348	1809	-1.4361763	2.74E-08	3.09E-06
ENSMUSG00000033082	Clec1a	358	516	508	312	220	312	196	277	-1.0566231	0.000649	0.0169473
ENSMUSG00000022235	Cmbl	3341	2976	4080	2515	1843	1792	1740	2039	-1.0921252	3.36E-07	2.84E-05
ENSMUSG00000014633	Cmc2	198	228	273	208	130	112	139	89	-1.2355414	6.80E-05	0.0027735
ENSMUSG00000000326	Comt	6518	7009	11287	6580	3290	3444	2654	2855	-1.6462656	6.32E-15	2.66E-12
ENSMUSG00000034361	Cpne2	1974	1305	1695	1299	950	1027	798	1073	-1.0046912	8.95E-05	0.0034784
ENSMUSG00000028607	Cpt2	3636	3323	3170	3161	2053	1777	1694	2452	-1.0268266	1.25E-06	9.06E-05

ENSMUSG00000026853	Crat	10754	11184	9707	8843	4860	3891	3316	5458	-1.5001267	4.92E-12	1.19E-09
ENSMUSG00000027357	Crls1	1642	1528	1565	1455	706	930	757	840	-1.2371422	8.60E-08	8.55E-06
ENSMUSG00000070495	Ctcfl	875	1031	794	749	418	567	426	573	-1.1023494	5.35E-05	0.0022697
ENSMUSG00000087382	Ctcflos	2167	2535	2179	1922	1247	1413	1138	1555	-1.0161737	5.45E-06	0.0003314
ENSMUSG00000052928	Ctif	1580	1384	2061	1781	880	1194	848	1180	-1.0185428	1.70E-05	0.0008835
ENSMUSG00000028988	Ctnnbip1	936	793	983	702	469	487	445	622	-1.0480443	0.0001256	0.0045672
ENSMUSG00000007891	Ctsd	15855	16256	23313	17172	11291	12055	9772	11029	-1.0046566	2.29E-08	2.63E-06
ENSMUSG00000016256	Ctsz	4984	5304	7345	6102	3222	4707	3461	3094	-1.0065473	7.93E-08	8.01E-06
ENSMUSG00000029417	Cxcl9	1464	1926	1715	938	822	1116	479	750	-1.2484798	7.29E-05	0.0029355
ENSMUSG00000063694	Cycs	2496	3059	3630	2616	1895	1177	1694	1996	-1.0796984	6.98E-06	0.0004076
ENSMUSG00000063415	Cyp26b1	462	988	473	716	360	317	406	286	-1.2399302	0.0001407	0.0050285
ENSMUSG00000022818	Cyp2ab1	73	80	75	75	41	32	19	38	-1.5052445	6.68E-05	0.0027304
ENSMUSG00000025479	Cyp2e1	119006	149732	37018	95679	12873	19527	7980	16330	-3.1493268	6.51E-17	3.58E-14
ENSMUSG00000028713	Cyp4b1	3281	4223	3591	2958	1720	2686	1368	1848	-1.1941691	2.10E-07	1.88E-05
ENSMUSG00000091586	Cyp4f17	1107	1359	590	1974	457	827	424	514	-1.4785731	2.56E-05	0.0012349
ENSMUSG00000079057	Cyp4v3	1934	2074	3250	1684	1022	1939	987	1302	-1.0733419	8.00E-05	0.0031741
ENSMUSG00000041134	Cyyr1	2007	3058	2370	2563	1215	2671	655	1507	-1.0421166	0.0011481	0.0264324
ENSMUSG00000055639	Dach1	568	417	526	356	276	232	241	265	-1.1759628	7.77E-05	0.0030981
ENSMUSG00000026385	Dbi	23177	19484	26428	18590	11939	7736	11047	10790	-1.361285	1.47E-10	2.89E-08
ENSMUSG00000042672	Dcst1	747	1111	870	759	344	327	287	313	-1.7521886	2.67E-10	4.88E-08
ENSMUSG00000027901	Dennd2d	971	2080	818	1221	467	950	446	451	-1.4639486	3.93E-05	0.0017659
ENSMUSG00000048489	Depp1	1161	1808	1884	845	295	884	135	269	-2.1899285	2.95E-06	0.0001933
ENSMUSG00000022555	Dgat1	5939	6274	5538	5529	1967	1817	1854	1720	-1.9566904	8.08E-26	1.32E-22
ENSMUSG00000011382	Dhdh	2876	3068	3277	2595	994	1364	1059	1175	-1.6641488	9.79E-16	4.60E-13
ENSMUSG00000022210	Dhrs4	1751	1922	2203	1729	1009	1070	914	1048	-1.2039928	4.95E-08	5.21E-06
ENSMUSG00000042569	Dhrs7b	1815	1889	1815	1701	1055	1186	758	956	-1.1679442	5.54E-07	4.44E-05
ENSMUSG00000025815	Dhtkd1	557	891	611	639	307	407	430	477	-1.0319972	0.0003491	0.0103319
ENSMUSG00000027314	Dll4	1288	964	1337	974	620	551	304	584	-1.4400842	1.87E-06	0.0001294
ENSMUSG00000048138	Dmrt2	1736	2475	2117	970	1021	1073	1024	1286	-1.034169	0.0005288	0.014389
ENSMUSG00000031093	Dock11	2458	2087	3361	2278	984	1718	846	1063	-1.4468556	1.06E-08	1.33E-06
ENSMUSG00000075419	Dolk	1816	2239	2166	1965	1061	946	949	1201	-1.264189	2.30E-08	2.63E-06
ENSMUSG00000020057	Dram1	6626	6453	7070	6337	2178	2483	2048	2896	-1.7552672	2.54E-20	2.20E-17
ENSMUSG00000027900	Dram2	2566	3009	3267	2555	1472	1961	1510	1552	-1.1102046	3.26E-08	3.59E-06
ENSMUSG00000004947	Dtx2	3446	3600	2801	2718	1447	1518	1336	1812	-1.3398339	1.27E-09	2.04E-07

ENSMUSG00000039384	Dusp10	1048	924	955	502	189	316	185	253	-2.1814636	8.04E-11	1.62E-08
ENSMUSG00000039661	Dusp26	154	179	194	147	91	139	68	95	-1.0820456	0.0010152	0.0241144
ENSMUSG00000003518	Dusp3	3443	4088	6593	3849	2186	3017	1934	2611	-1.1725971	2.04E-07	1.84E-05
ENSMUSG00000031530	Dusp4	902	1172	934	1049	470	697	460	632	-1.1445315	1.39E-05	0.0007413
ENSMUSG00000031383	Dusp9	92	311	253	112	54	58	83	125	-1.5490913	0.0011108	0.0258635
ENSMUSG00000027490	E2f1	361	236	411	233	161	232	146	155	-1.1433174	0.0004524	0.0127032
ENSMUSG00000072596	Ear2	550	378	533	671	223	314	197	204	-1.4765769	4.10E-07	3.41E-05
ENSMUSG00000057098	Ebf1	6111	7057	6265	5020	2387	3926	2506	3948	-1.2444148	2.05E-08	2.43E-06
ENSMUSG00000053898	Ech1	11832	15018	14075	11533	5625	6391	6100	6910	-1.3628633	1.63E-13	5.30E-11
ENSMUSG00000024132	Eci1	3059	4074	3955	3135	1814	1600	1467	1668	-1.4092638	7.93E-12	1.83E-09
ENSMUSG00000021416	Eci3	481	383	495	258	266	130	64	87	-1.8552872	4.30E-05	0.0019011
ENSMUSG00000022122	Ednrb	2004	3144	3191	2952	1090	1636	1003	1111	-1.5184657	2.36E-11	5.10E-09
ENSMUSG00000036611	Eepd1	6970	8038	9377	8867	5326	2933	5336	5964	-1.0341639	8.30E-06	0.0004755
ENSMUSG00000003070	Efna2	153	86	147	91	49	63	66	55	-1.3326661	0.0002371	0.0076799
ENSMUSG00000074364	Ehd2	36125	36463	40039	28014	15719	13651	14294	19255	-1.4510293	1.42E-12	3.89E-10
ENSMUSG00000022853	Ehhadh	1351	1914	1324	2205	768	668	510	802	-1.5861184	6.74E-09	8.95E-07
ENSMUSG00000031490	Eif4ebp1	8042	7671	9339	7098	2821	3431	2125	3018	-1.7941691	1.36E-19	1.11E-16
ENSMUSG00000022217	Emc9	1006	981	1329	1035	618	485	526	558	-1.2737752	7.35E-07	5.69E-05
ENSMUSG00000024053	Emilin2	2613	2226	4492	2639	1511	1552	1239	1632	-1.2932498	7.47E-08	7.57E-06
ENSMUSG00000091575	ENSMUSG000000	2913	4167	1694	2752	217	735	176	217	-3.4490803	2.05E-15	9.15E-13
ENSMUSG00000019978	Epb41l2	11340	9812	15288	8101	5051	5566	5701	5906	-1.2960273	9.34E-10	1.52E-07
ENSMUSG00000037577	Ephx3	125	124	123	92	89	66	53	66	-1.0535821	0.0023408	0.0450204
ENSMUSG00000055493	Epm2a	511	511	567	447	264	325	306	349	-1.0061201	0.0001707	0.0058873
ENSMUSG00000001946	Esam	3506	3773	3778	3517	2234	2432	1814	2218	-1.0396415	2.64E-08	3.00E-06
ENSMUSG00000025366	Esyt1	12397	10828	13589	12008	6095	6513	4473	6764	-1.3243077	2.49E-12	6.45E-10
ENSMUSG00000043251	Exoc3l	221	239	196	162	117	96	53	99	-1.4628403	4.64E-05	0.0020216
ENSMUSG00000031444	F10	106	104	145	151	66	60	52	84	-1.2221013	0.000263	0.0083097
ENSMUSG00000062515	Fabp4	237642	314753	386768	217601	60692	85520	50384	67029	-2.4345933	6.61E-27	1.26E-23
ENSMUSG00000030630	Fah	3815	5431	3268	3605	2588	2534	1929	2526	-1.0532275	2.43E-06	0.0001634
ENSMUSG00000037709	Fam13a	6058	7672	4122	8592	1322	4067	1590	1706	-1.9298107	7.72E-10	1.29E-07
ENSMUSG00000057858	Fam204a	742	863	1034	846	514	532	570	459	-1.0361825	3.58E-05	0.0016416
ENSMUSG00000021414	Fam217a	126	189	286	120	62	103	75	74	-1.495881	7.35E-05	0.00295
ENSMUSG00000028439	Fam219a	732	647	668	615	416	428	251	451	-1.0801795	0.000166	0.0057715
ENSMUSG00000035764	Fbxo45	2414	3194	2165	3453	1108	946	1008	1224	-1.6716446	7.66E-13	2.15E-10

ENSMUSG00000026938	Fcna	1152	3532	1876	3214	707	1145	492	655	-2.0037277	5.73E-09	7.69E-07
ENSMUSG00000032051	Fdx1	1082	1322	1202	1283	755	742	685	759	-1.0221775	1.56E-05	0.0008212
ENSMUSG00000051314	Ffar2	1437	1450	1470	1789	191	207	80	246	-3.3706848	2.85E-22	3.37E-19
ENSMUSG00000054200	Ffar4	880	706	780	488	310	213	279	330	-1.6280327	2.89E-07	2.49E-05
ENSMUSG00000008090	Fgfrl1	7221	8245	9090	5823	3850	3346	3916	4818	-1.220596	1.03E-08	1.31E-06
ENSMUSG00000048486	Fitm2	5310	7337	5747	5926	2860	3284	2409	3653	-1.2895726	2.02E-10	3.81E-08
ENSMUSG00000000838	Fmr1	6981	5586	17672	6511	5426	4000	4475	4917	-1.2331237	4.78E-05	0.0020678
ENSMUSG00000056602	Fry	5031	4297	8001	4162	2727	3123	2316	3585	-1.1579124	8.24E-07	6.29E-05
ENSMUSG00000029581	Fscn1	3850	3093	2672	2268	1601	1701	1056	1674	-1.287483	3.59E-07	3.01E-05
ENSMUSG00000050708	Ftl1	25508	24710	28419	27919	13830	16548	9023	12193	-1.3419965	3.49E-12	8.69E-10
ENSMUSG00000031400	G6pdx	5607	6890	9051	4586	2835	2406	2461	2666	-1.6222364	8.16E-13	2.28E-10
ENSMUSG00000022707	Gbe1	1550	1559	1854	1379	755	632	1103	1079	-1.1126733	4.50E-05	0.0019661
ENSMUSG00000025127	Gcgr	101	121	59	96	39	43	19	20	-1.9475786	5.67E-06	0.0003413
ENSMUSG00000034424	Gcsh	1795	1767	2567	1871	982	881	1030	1089	-1.2875301	2.95E-08	3.31E-06
ENSMUSG00000040888	Gfer	674	934	753	790	428	454	372	375	-1.2471446	1.57E-06	0.0001093
ENSMUSG00000051335	Gfod1	937	1520	952	854	610	936	342	748	-1.0087307	0.0026035	0.0487853
ENSMUSG00000055737	Ghr	40153	50963	34986	31273	19586	22460	10999	24656	-1.324775	2.41E-07	2.11E-05
ENSMUSG00000040006	Ginm1	5789	5716	7125	4882	3418	3260	3814	3698	-1.0187272	1.12E-07	1.08E-05
ENSMUSG00000034872	Gipc3	120	105	130	97	75	58	36	67	-1.2249877	0.0007193	0.0184508
ENSMUSG00000030406	Gipr	462	553	293	433	176	234	113	212	-1.552929	6.03E-06	0.0003573
ENSMUSG00000034520	Gjc1	786	1242	1030	1062	479	597	592	602	-1.1515429	9.13E-06	0.0005168
ENSMUSG00000030048	Gkn3	98	110	145	65	43	40	67	56	-1.3095352	0.0008075	0.020184
ENSMUSG00000036395	Glb1l2	15762	14667	12370	10983	5514	2690	3039	3108	-2.1964843	1.52E-16	7.95E-14
ENSMUSG00000021102	Glrx5	2160	2431	3008	1390	730	551	886	1100	-1.7462406	1.43E-08	1.75E-06
ENSMUSG00000100954	Gm10138	60	53	83	91	40	48	33	26	-1.2471034	0.0006446	0.0168547
ENSMUSG00000097773	Gm10614	89	114	92	94	41	64	37	30	-1.4838807	3.61E-05	0.0016519
ENSMUSG00000075514	Gm13375	474	292	607	320	187	248	278	265	-1.0837639	0.00091	0.0221178
ENSMUSG00000094786	Gm14403	230	243	351	209	133	181	132	154	-1.0786561	0.0004428	0.0125095
ENSMUSG00000074466	Gm15417	333	381	323	333	170	127	254	140	-1.2754886	9.33E-05	0.0035986
ENSMUSG00000083443	Gm15519	494	760	1267	563	133	49	104	153	-3.0706927	1.50E-12	4.06E-10
ENSMUSG00000086914	Gm16124	65	101	74	113	37	66	51	40	-1.1581073	0.0012843	0.0285855
ENSMUSG00000089941	Gm16168	88	92	91	108	9	5	4	13	-3.8669457	3.43E-17	1.96E-14
ENSMUSG00000084512	Gm22482	262	311	300	274	26	17	36	37	-3.581387	1.65E-21	1.76E-18
ENSMUSG00000076258	Gm23935	25050	35403	27286	19713	14881	12677	6331	16535	-1.3891405	1.01E-06	7.50E-05

ENSMUSG00000101925	Gm28156	192	292	269	278	36	21	51	53	-2.9470206	4.61E-14	1.72E-11
ENSMUSG00000117664	Gm36860	2456	3491	3093	2957	259	122	244	417	-3.7940814	5.25E-27	1.04E-23
ENSMUSG00000103047	Gm37310	130	171	122	417	94	70	75	80	-1.6491025	9.93E-05	0.0037834
ENSMUSG00000112246	Gm40761	198	76	124	72	17	19	22	23	-2.8386531	1.37E-09	2.16E-07
ENSMUSG00000106073	Gm42892	83	177	122	89	46	56	41	65	-1.4771	0.0001422	0.0050649
ENSMUSG00000105135	Gm43667	153	140	204	118	87	95	82	96	-1.0647691	0.001008	0.0240346
ENSMUSG00000107655	Gm44220	365	399	340	433	157	187	183	182	-1.4078191	2.83E-07	2.45E-05
ENSMUSG00000089783	Gm454	1109	1066	692	413	33	3	8	27	-5.8067271	2.94E-19	2.23E-16
ENSMUSG00000109674	Gm45470	3373	3065	1658	2477	518	675	309	556	-2.6772447	1.14E-17	6.90E-15
ENSMUSG00000107838	Gm45769	143	121	151	126	99	73	80	72	-1.0259139	0.0013982	0.0307094
ENSMUSG00000114709	GM47920	77	91	80	79	40	60	40	44	-1.1299093	0.0008212	0.0204504
ENSMUSG00000117814	Gm50431	348	399	425	390	236	183	284	245	-1.001692	0.0004811	0.0134013
ENSMUSG00000072624	Gm5460	167	98	173	70	7	3	8	6	-4.6669683	2.52E-18	1.71E-15
ENSMUSG00000029633	Gm5578	91	86	129	73	27	10	15	19	-2.6835365	8.64E-10	1.42E-07
ENSMUSG00000097440	Gm6277	245	252	212	156	72	98	91	94	-1.5929012	1.51E-06	0.0001063
ENSMUSG00000027523	Gnas	1478	726	727	428	385	493	397	498	-1.2476691	0.0008813	0.0215811
ENSMUSG00000031985	Gnpat	8130	8522	6987	6190	3889	4444	3115	4296	-1.2269585	1.62E-09	2.50E-07
ENSMUSG00000042532	Golga7b	79	50	205	79	54	53	10	34	-1.7261713	0.0018782	0.0381607
ENSMUSG00000024978	Gpam	8189	11338	9399	13212	4845	4430	4613	5057	-1.4328641	5.21E-14	1.90E-11
ENSMUSG00000026827	Gpd2	1762	1925	1469	1567	557	592	474	540	-1.937282	7.25E-15	2.98E-12
ENSMUSG00000022579	Gpihbp1	2871	4026	4249	3016	1510	2896	1213	1956	-1.2119295	2.20E-06	0.0001502
ENSMUSG00000046856	Gpr1	52	66	81	68	48	26	30	35	-1.2125697	0.0014059	0.0308221
ENSMUSG00000037661	Gpr160	597	1013	838	760	349	778	319	511	-1.0247981	0.0015023	0.0324108
ENSMUSG00000044317	Gpr4	493	554	507	497	286	442	194	322	-1.0274272	0.0006123	0.0161702
ENSMUSG00000022546		2683	2613	3241	2288	282	269	248	318	-3.5663214	7.08E-46	4.91E-42
ENSMUSG00000063856	Gpx1	23766	25938	27155	21924	7042	10204	6419	7615	-1.9632744	8.52E-25	1.28E-21
ENSMUSG00000075706	Gpx4	16557	17341	20745	14503	9646	8933	7474	10803	-1.197097	1.25E-09	2.01E-07
ENSMUSG00000004344	Gpx5	710	0	0	0	1	0	5	0	-7.1329219	0.001596	0.0338657
ENSMUSG00000050069	Grem2	864	1482	579	1738	432	559	416	432	-1.6339109	1.52E-06	0.0001067
ENSMUSG00000020656	Grhl1	342	483	165	340	213	198	142	164	-1.1985869	0.0012826	0.0285578
ENSMUSG00000022564	Grina	19530	19590	16770	17725	9553	13462	9788	10093	-1.0854909	1.77E-09	2.70E-07
ENSMUSG00000025934	Gsta3	4167	3552	1976	2790	1338	869	1448	1017	-1.7196111	2.85E-09	4.08E-07
ENSMUSG00000025068	Gsto1	1150	1410	1382	1081	383	483	591	544	-1.6228429	1.41E-09	2.20E-07
ENSMUSG00000038155	Gstp2	307	188	30	259	96	68	77	16	-1.9168529	0.0025685	0.0483017

ENSMUSG00000021033	Gstz1	20374	19267	17755	14539	8785	5460	9371	10379	-1.3695641	9.63E-09	1.23E-06
ENSMUSG00000003865	Gys1	3996	4419	3290	3379	1421	819	1236	1570	-1.8660946	5.02E-13	1.46E-10
ENSMUSG00000051627	H1f4	155	88	279	82	62	76	22	33	-1.944869	0.0001212	0.0044525
ENSMUSG00000018102	H2bc4	2081	1723	2885	1817	1529	1317	887	1146	-1.0888015	1.97E-05	0.0009995
ENSMUSG00000047246	H2bc6	200	155	373	130	122	116	81	55	-1.4896814	0.0003636	0.0106227
ENSMUSG00000073411	H2-D1	63467	72169	48188	52412	28545	39962	27546	27222	-1.2524197	8.99E-10	1.47E-07
ENSMUSG00000056116	H2-T22	4887	5809	4146	3938	2196	2695	1997	1901	-1.406255	2.68E-11	5.69E-09
ENSMUSG00000060981	H4c8	298	219	494	228	52	71	52	40	-2.819855	1.47E-13	4.82E-11
ENSMUSG00000028980	H6pd	14022	20905	17000	19188	6022	10270	7175	8748	-1.4414279	6.08E-13	1.72E-10
ENSMUSG00000049241	Hcar1	5154	4144	3257	5087	814	1229	435	851	-2.7145393	3.91E-21	3.94E-18
ENSMUSG00000032338	Hcn4	187	285	149	185	93	98	82	82	-1.4870548	1.29E-05	0.000695
ENSMUSG00000030532	Hddc3	529	348	386	517	245	254	86	122	-1.6326231	1.90E-05	0.0009653
ENSMUSG00000042770	Hebp1	2618	2668	3669	1978	1457	1137	1607	1747	-1.1628044	3.68E-06	0.0002348
ENSMUSG00000031209	Heph	3482	3161	3072	3434	996	1015	1092	1165	-1.9140464	3.81E-21	3.92E-18
ENSMUSG00000040289	Hey1	401	430	544	394	181	188	100	170	-1.7602405	1.94E-08	2.32E-06
ENSMUSG00000028864	Hgf	206	217	261	185	124	166	132	100	-1.0365757	0.000654	0.017044
ENSMUSG00000029776	Hibadh	6519	7558	6693	6234	3555	3738	3934	4117	-1.1101462	8.72E-10	1.43E-07
ENSMUSG00000041426	Hibch	4014	4787	3926	3636	1468	1265	1450	1760	-1.752899	3.88E-16	1.94E-13
ENSMUSG00000020928	Higd1b	218	214	205	161	89	118	69	84	-1.4559989	6.98E-06	0.0004076
ENSMUSG00000061436	Hipk2	2940	5055	4826	2982	2234	2122	1751	3431	-1.0158219	0.0001392	0.0049819
ENSMUSG00000055632	Hmcn2	1386	1805	1118	1091	547	574	342	465	-1.7956053	1.75E-09	2.68E-07
ENSMUSG00000046711	Hmga1	604	651	1593	762	397	696	340	299	-1.3543948	0.0001832	0.0062388
ENSMUSG00000026986	Hnmt	950	845	1070	735	370	360	451	378	-1.5001124	2.99E-08	3.34E-06
ENSMUSG00000001657	Hoxc8	4063	4630	4110	3022	1892	1994	2161	2689	-1.1551375	1.70E-07	1.56E-05
ENSMUSG00000025630	Hprt	2859	3241	4981	3047	1921	1747	1996	1736	-1.2152401	2.18E-08	2.54E-06
ENSMUSG00000071001	Hrct1	1207	1210	1153	1044	566	1120	507	672	-1.0038775	0.0004292	0.0122091
ENSMUSG00000027195	Hsd17b12	3940	4935	4443	3604	1935	1625	1989	2067	-1.4426379	3.05E-12	7.66E-10
ENSMUSG00000024507	Hsd17b4	6808	8566	10304	6412	3218	2726	3139	3382	-1.6500733	2.68E-15	1.19E-12
ENSMUSG00000028383	Hsdl2	4871	5271	7348	3814	2210	2330	1666	2192	-1.6372634	4.81E-13	1.41E-10
ENSMUSG00000033249	Hsf4	156	155	101	124	69	95	79	59	-1.1390409	0.0008515	0.0210185
ENSMUSG00000074793	Hspa12b	2942	2895	3504	2543	1568	2661	1194	1906	-1.005065	2.92E-05	0.0013775
ENSMUSG00000041548	Hspb8	3258	2671	4690	2573	1894	1231	1297	1397	-1.4611848	7.71E-09	1.01E-06
ENSMUSG00000039745	Htatip2	855	875	1012	843	660	533	385	425	-1.1292529	4.23E-05	0.0018775
ENSMUSG00000007872	Id3	2649	2811	5363	3906	2057	2819	1555	1806	-1.123937	3.34E-06	0.0002157

ENSMUSG00000079017	Ifi27l2a	20679	14912	19556	12641	1066	911	1267	741	-4.3868971	4.11E-63	7.59E-59
ENSMUSG00000059108	Ifitm6	176	156	159	188	88	84	85	66	-1.3615352	8.28E-06	0.0004748
ENSMUSG00000033581	Igf2bp2	425	736	513	617	213	404	230	232	-1.3942983	6.69E-06	0.0003935
ENSMUSG00000096108	Ighv11-2	32	153	45	1342	131	13	16	11	-3.3623768	0.0014541	0.0316283
ENSMUSG00000094345	Igkv14-126	63	333	101	2584	312	84	84	48	-2.7282885	0.0023236	0.0448005
ENSMUSG00000047182	Irs3	2419	2504	2175	1410	403	602	279	362	-2.6914877	3.60E-18	2.35E-15
ENSMUSG00000051243	Islr2	273	203	344	308	99	94	98	104	-1.7920103	4.47E-09	6.12E-07
ENSMUSG00000024601	Isoc1	5548	5333	6104	4935	2106	1938	2199	2231	-1.6608845	6.71E-19	5.03E-16
ENSMUSG00000086784	Isoc2a	2277	2599	2367	2393	1260	1152	1333	1604	-1.1361037	1.77E-07	1.62E-05
ENSMUSG00000032243	Itga11	579	435	438	346	194	373	201	198	-1.2203617	0.0001838	0.0062443
ENSMUSG00000027111	Itga6	4632	4786	5882	4171	3105	3140	2049	3362	-1.0317452	8.97E-07	6.78E-05
ENSMUSG00000062352	Itgb1bp1	1767	2570	2623	2373	917	861	780	968	-1.6877488	1.30E-13	4.32E-11
ENSMUSG00000025780	Itih5	15292	22210	30181	16446	5417	8667	5217	5659	-2.0535216	3.43E-18	2.27E-15
ENSMUSG00000001552	Jup	8441	9799	9884	7311	3739	4494	3314	4468	-1.444824	1.14E-13	3.84E-11
ENSMUSG00000058743	Kcnj14	235	96	475	123	71	30	108	106	-1.8191901	0.0008439	0.0208577
ENSMUSG00000043673	Kcns3	1234	1080	1356	924	271	210	68	82	-3.1639167	2.86E-13	8.83E-11
ENSMUSG00000041633	Kctd12b	1418	2012	1892	1341	651	979	696	848	-1.3735096	9.78E-08	9.57E-06
ENSMUSG00000033287	Kctd17	3266	2875	3343	3106	1932	1691	1676	2018	-1.0697748	4.80E-08	5.06E-06
ENSMUSG00000062960	Kdr	6309	7767	5852	6230	3302	4054	2038	3139	-1.3666943	5.24E-10	9.11E-08
ENSMUSG00000029195	Klb	2195	2113	1055	1757	680	664	398	706	-1.8463311	4.03E-09	5.57E-07
ENSMUSG00000073209	Klf14	118	208	429	182	64	27	89	129	-1.8488806	0.0002957	0.0090652
ENSMUSG00000078234	Klhdc7a	10075	14865	10183	8636	3637	3753	3615	5954	-1.6646823	1.66E-11	3.65E-09
ENSMUSG00000031605	Klhl2	5395	6121	4152	4897	2364	2154	2307	2676	-1.410232	1.20E-11	2.68E-09
ENSMUSG00000019718	L3hypdh	832	997	865	837	486	333	418	481	-1.3241122	1.59E-06	0.0001111
ENSMUSG00000039682	Lap3	2257	2403	2757	2039	1176	1199	1164	1163	-1.3010036	8.13E-10	1.34E-07
ENSMUSG00000022257	Laptm4b	2635	2533	2779	2658	1539	1459	1392	1479	-1.1428766	4.58E-09	6.26E-07
ENSMUSG00000025762	Larp1b	920	893	1012	802	585	390	483	489	-1.1823661	1.39E-05	0.000742
ENSMUSG00000035202	Lars2	17398	23740	18755	19389	14478	14087	7714	11596	-1.0244552	2.32E-06	0.0001572
ENSMUSG00000032401	Lctl	645	346	1039	291	123	233	40	156	-2.3817966	3.33E-06	0.0002151
ENSMUSG00000063229	Ldha	14268	14171	16102	12649	7860	6580	7817	8484	-1.1829683	2.48E-10	4.58E-08
ENSMUSG00000059201	Lep	16762	22402	53162	19555	7018	2805	6213	8838	-2.4215026	1.97E-10	3.75E-08
ENSMUSG00000068220	Lgals1	17319	16491	31273	16672	9956	6621	11465	10895	-1.3415223	9.29E-08	9.14E-06
ENSMUSG00000024972	Lgals12	14765	15236	9303	11472	2463	2279	2207	3060	-2.6436196	5.43E-29	1.37E-25
ENSMUSG00000033880	Lgals3bp	10077	9320	6640	7499	4829	4884	5880	4422	-1.0495975	4.94E-07	4.02E-05

ENSMUSG00000045312	Lhfpl2	3594	2466	7291	3037	1731	1052	1537	1748	-1.6991259	5.06E-08	5.30E-06
ENSMUSG00000024395	Lims2	3243	3762	4711	3198	1663	2674	1417	1910	-1.2632361	1.70E-08	2.07E-06
ENSMUSG00000003123	Lipe	47531	50524	50272	38972	5727	6631	3778	8352	-3.2322014	3.69E-38	1.57E-34
ENSMUSG00000030725	Lipt2	237	150	228	204	126	97	115	101	-1.1841487	0.0001862	0.0063099
ENSMUSG00000028266	Lmo4	3820	4117	4016	3724	2216	2213	1931	2170	-1.1715826	1.47E-10	2.88E-08
ENSMUSG00000033446	Lpar6	1812	1734	1953	1329	943	1204	781	995	-1.1041156	8.07E-06	0.0004644
ENSMUSG00000026623	Lpgat1	12165	13406	23535	15207	8204	5723	7276	7670	-1.4249797	1.87E-10	3.59E-08
ENSMUSG00000020593	Lpin1	9021	12164	12395	8589	3690	4201	3611	4602	-1.6833423	4.95E-17	2.77E-14
ENSMUSG00000015568	Lpl	200152	233206	219214	143877	98885	90404	76233	114024	-1.3664183	3.16E-10	5.71E-08
ENSMUSG00000037095	Lrg1	9553	10067	8025	13231	4739	4864	3320	5075	-1.4690986	1.24E-12	3.40E-10
ENSMUSG00000063458	Lrmda	302	319	325	300	134	168	159	147	-1.3309197	1.48E-06	0.0001043
ENSMUSG00000027961	Lrrc39	572	590	1042	451	120	86	115	158	-2.7460348	6.17E-14	2.21E-11
ENSMUSG00000045201	Lrrc3b	93	87	111	87	64	37	36	44	-1.3412283	0.0002199	0.0072454
ENSMUSG00000046908	Ltb4r1	92	85	131	131	61	60	67	56	-1.1234743	0.0005553	0.0149507
ENSMUSG00000020377	Ltc4s	842	1023	1588	1100	269	332	172	271	-2.4108824	2.89E-15	1.26E-12
ENSMUSG00000026344	Lypd1	124	82	40	87	26	18	3	20	-2.607234	4.62E-06	0.0002871
ENSMUSG00000069515	Lyz1	1463	968	1166	1629	292	227	330	358	-2.3904808	6.69E-16	3.23E-13
ENSMUSG00000110884	m47416	149	144	138	97	36	35	55	74	-1.6914733	1.83E-05	0.0009418
ENSMUSG00000036278	Macrod1	1030	1256	890	829	398	401	500	569	-1.3985273	1.37E-06	9.78E-05
ENSMUSG00000031147	Magix	181	236	289	236	147	127	116	155	-1.068325	0.0004331	0.0122851
ENSMUSG00000038886	Man2a2	9042	8295	7131	8309	4740	5813	4146	4550	-1.0708501	5.13E-09	6.95E-07
ENSMUSG00000023307	Marchf5	3991	4434	4473	3292	2247	2244	2257	2609	-1.0858134	4.45E-08	4.75E-06
ENSMUSG00000026390	Marco	63	111	62	99	39	29	34	48	-1.43803	0.0002375	0.0076841
ENSMUSG00000049285	Mblac1	220	213	168	135	134	108	96	100	-1.0524063	0.0018337	0.0374857
ENSMUSG00000045569	Mc2r	736	429	869	535	152	270	167	179	-2.0455862	3.51E-10	6.31E-08
ENSMUSG00000032135	Mcam	16798	15285	20194	14966	9703	5935	7755	9917	-1.2913395	3.33E-09	4.71E-07
ENSMUSG00000027353	Mcm8	196	190	228	202	122	169	105	96	-1.03012	0.0006813	0.0176144
ENSMUSG00000032418	Me1	8114	7449	7108	5569	4108	2525	3335	4679	-1.2355397	3.07E-07	2.61E-05
ENSMUSG00000028910	Mecr	1445	1409	1497	1211	782	661	635	827	-1.2274421	1.03E-06	7.65E-05
ENSMUSG00000056476	Med12l	522	556	585	500	220	330	358	308	-1.1285891	3.92E-05	0.0017646
ENSMUSG00000039270	Megf9	1589	1598	1852	1507	844	732	741	820	-1.3488441	8.06E-09	1.04E-06
ENSMUSG00000036144	Meox2	1358	1227	1458	904	501	488	343	523	-1.7130725	2.16E-09	3.23E-07
ENSMUSG00000018169	Mfng	3580	2583	5397	2017	591	792	482	751	-2.6735562	4.08E-18	2.63E-15
ENSMUSG00000019080	Mfsd3	297	263	316	276	192	170	145	150	-1.1003057	0.0001207	0.0044433

ENSMUSG00000033174	Mgll	44340	48331	47443	35529	18450	18355	10732	18188	-1.717021	4.81E-15	2.05E-12
ENSMUSG00000008540	Mgst1	20568	25000	21549	18581	9316	10393	9068	10214	-1.435453	5.05E-15	2.14E-12
ENSMUSG00000074604	Mgst2	287	365	404	306	116	232	117	143	-1.47079	4.41E-06	0.0002761
ENSMUSG00000026688	Mgst3	5721	7427	8052	6246	1692	1739	1469	1781	-2.3281774	5.40E-33	1.76E-29
ENSMUSG00000026858	Miga2	3839	4326	3256	3213	2428	1724	1656	2552	-1.0992746	2.89E-06	0.0001898
ENSMUSG00000005373	Mlxipl	2983	3797	2272	2896	1623	1752	1961	1816	-1.0411533	2.84E-06	0.0001873
ENSMUSG00000039616	Mocos	552	542	709	559	309	385	252	305	-1.2113878	5.56E-06	0.0003362
ENSMUSG00000012187	Mogat1	616	626	503	548	313	193	209	232	-1.56427	2.10E-07	1.88E-05
ENSMUSG00000074813	Morrbid	94	59	129	80	36	60	38	51	-1.2600783	0.0008687	0.0213275
ENSMUSG00000079550	Mpp4	112	112	148	76	51	50	49	87	-1.2060541	0.0016663	0.0349152
ENSMUSG00000039956	Mrap	3305	4859	3629	2798	872	631	895	1027	-2.3823675	1.72E-19	1.38E-16
ENSMUSG00000024730	Ms4a8a	154	166	139	187	85	80	58	82	-1.3697977	1.65E-05	0.0008579
ENSMUSG00000063011	Msln	1422	981	828	1142	517	621	352	571	-1.3895548	2.69E-06	0.000178
ENSMUSG00000075705	Msrb1	2014	2762	2314	2130	1864	1011	1556	1089	-1.0237713	9.18E-05	0.0035475
ENSMUSG00000032584	Mst1r	1467	1645	1775	1765	922	891	765	1074	-1.1488667	7.90E-07	6.07E-05
ENSMUSG00000028654	Mycl	913	732	1204	1546	255	716	256	286	-1.8412242	3.46E-07	2.92E-05
ENSMUSG00000046916	Myct1	638	652	846	628	415	371	327	415	-1.1406824	1.72E-05	0.0008957
ENSMUSG00000061816	Myl1	1032	908	853	930	386	330	542	698	-1.2124746	8.98E-05	0.0034883
ENSMUSG00000061086	Myl4	110	139	306	148	80	96	101	107	-1.1462876	0.0023282	0.0448567
ENSMUSG00000041132	N4bp2l1	1349	1368	1087	1061	608	872	655	650	-1.1156925	1.49E-05	0.0007847
ENSMUSG00000058799	Nap1l1	9710	10734	17706	10396	4840	5922	5633	5794	-1.4152999	7.64E-12	1.77E-09
ENSMUSG00000055430	Nap1l5	160	108	117	112	74	77	70	69	-1.0768955	0.0010128	0.0240972
ENSMUSG00000044968	Napepld	1366	1603	1469	1260	936	619	934	979	-1.0011458	0.0001574	0.0055092
ENSMUSG00000048142	Nat8l	3222	2391	2621	1850	858	875	471	1019	-1.9489747	2.56E-11	5.48E-09
ENSMUSG00000020181	Nav3	940	889	923	906	475	693	445	529	-1.0743209	2.36E-05	0.001155
ENSMUSG00000040280	Ndufa4l2	577	559	645	533	311	275	232	344	-1.2811474	3.63E-06	0.0002326
ENSMUSG00000092274	Neat1	57653	52868	60082	69985	32754	37735	31270	29310	-1.1662485	1.49E-12	4.05E-10
ENSMUSG00000040037	Negr1	1630	1602	1593	1086	303	595	310	317	-2.276717	7.66E-14	2.67E-11
ENSMUSG00000067786	Nnat	53635	33338	53741	31640	16455	4428	17623	20677	-1.8133903	1.34E-06	9.62E-05
ENSMUSG00000032271	Nnmt	3281	2942	4086	2942	2211	903	1411	2082	-1.2732762	7.82E-06	0.0004507
ENSMUSG00000048616	Nog	237	161	397	301	164	84	96	162	-1.3716848	0.0003605	0.0105626
ENSMUSG00000014776	Nol3	575	354	815	490	225	125	169	180	-1.9470007	2.26E-08	2.60E-06
ENSMUSG00000030835	Nomo1	2965	3217	3960	2873	2069	1836	1732	2059	-1.0446983	2.22E-07	1.97E-05
ENSMUSG00000104350	Gm38244	636	795	327	549	89	244	111	103	-2.4125112	2.45E-09	3.61E-07

ENSMUSG00000086679	Gm15551	412	352	379	277	89	78	102	80	-2.3208101	4.66E-13	1.38E-10
ENSMUSG00000030664	Sox6os	172	142	99	181	57	23	30	35	-2.3089246	3.01E-08	3.35E-06
ENSMUSG00000106871	Gm3289	451	461	292	391	101	110	86	118	-2.2434413	3.00E-12	7.57E-10
ENSMUSG00000086584	Gm12002	189	211	288	169	54	71	43	62	-2.1912297	1.81E-10	3.49E-08
ENSMUSG00000106709	Gm30270	403	369	226	301	90	101	105	67	-2.1488653	3.91E-10	6.89E-08
ENSMUSG00000117263	Gm35290	159	123	175	182	39	55	32	51	-2.1383207	3.56E-10	6.34E-08
ENSMUSG00000101089	2610016A17Rik	430	526	721	321	126	221	136	103	-2.0805846	5.11E-09	6.93E-07
ENSMUSG00000097177	9330159M07Rik	269	247	174	233	63	62	83	69	-2.0324452	1.10E-09	1.77E-07
ENSMUSG00000109130	Gm45187	96	70	110	86	23	30	17	38	-2.0291012	2.24E-07	1.99E-05
ENSMUSG00000064349	mt-Tc	325	356	814	207	105	151	158	143	-1.9003574	4.48E-06	0.0002796
ENSMUSG00000115100	Gm34934	361	238	347	259	96	88	108	105	-1.8918612	2.74E-09	3.97E-07
ENSMUSG00000086727	4931428L18Rik	269	164	175	148	86	40	62	75	-1.8119595	4.54E-06	0.0002828
ENSMUSG00000116903	Gm19522	274	268	121	121	49	58	107	68	-1.7940214	5.31E-05	0.0022583
ENSMUSG00000114796	A930028N01Rik	48	136	65	97	34	38	12	40	-1.7699554	0.0002375	0.0076841
ENSMUSG00000020826	Nos2	509	364	394	324	193	199	107	183	-1.5260013	3.62E-06	0.0002321
ENSMUSG00000015468	Notch4	2287	2335	2577	1828	1123	1684	920	1245	-1.1681503	1.06E-06	7.77E-05
ENSMUSG00000022206	Npr3	18974	22047	36590	17112	5546	6216	3607	8912	-2.2471783	1.39E-14	5.64E-12
ENSMUSG00000026826	Nr4a2	115	94	142	132	69	66	40	43	-1.4326465	3.77E-05	0.001709
ENSMUSG00000026398	Nr5a2	175	169	127	147	54	83	30	64	-1.7290699	4.53E-06	0.0002827
ENSMUSG00000078202	Nrarp	226	144	237	257	115	122	54	109	-1.394851	9.06E-05	0.0035142
ENSMUSG00000032311	Nrg4	1807	2078	3247	1376	358	346	335	554	-2.7005993	6.59E-17	3.59E-14
ENSMUSG00000024228	Nudt12	760	1071	922	840	543	515	452	663	-1.0146032	0.0001814	0.0061922
ENSMUSG00000045211	Nudt18	1577	1734	2489	1428	716	733	822	1045	-1.4083855	1.46E-07	1.37E-05
ENSMUSG00000031767	Nudt7	1164	1116	1020	922	659	646	507	713	-1.0395284	7.45E-05	0.0029842
ENSMUSG00000026525	Opn3	672	553	709	444	35	62	22	53	-4.0946906	1.68E-25	2.66E-22
ENSMUSG00000039196	Orm1	1997	1315	2130	659	657	330	714	731	-1.6166686	3.59E-05	0.0016433
ENSMUSG00000049112	Oxtr	379	1480	2299	1334	468	218	446	722	-1.8124182	9.49E-05	0.0036453
ENSMUSG00000054582	Pabpc1l	112	115	129	121	55	52	63	75	-1.2433774	0.0001317	0.0047506
ENSMUSG00000025330	Padi4	209	238	177	239	143	88	83	151	-1.1715793	0.0007269	0.0185907
ENSMUSG00000033377	Palmd	7054	7843	10231	4572	1495	2433	1644	3145	-2.0685555	5.04E-13	1.46E-10
ENSMUSG00000064225	Paqr9	967	1162	1715	504	45	34	62	39	-4.8818686	1.94E-28	4.48E-25
ENSMUSG00000023249	Parp3	2799	3131	3465	2644	1664	1863	1743	1969	-1.0269053	2.07E-07	1.86E-05
ENSMUSG00000112129	Pbld1	302	397	206	348	198	255	85	143	-1.1903809	0.0017514	0.0362569
ENSMUSG00000027513	Pck1	59075	80124	85886	35260	6580	4623	3730	7494	-3.827981	5.63E-32	1.74E-28

ENSMUSG00000015354	Pcolce2	1044	815	1650	1214	434	487	507	484	-1.5835661	7.83E-09	1.02E-06
ENSMUSG00000030513	Pcsk6	1935	1299	1217	1657	676	864	559	879	-1.3366922	9.05E-07	6.84E-05
ENSMUSG00000024892	Pcx	32855	34627	19627	21696	16138	11475	11322	21094	-1.1568274	2.54E-05	0.0012287
ENSMUSG00000030671	Pde3b	12512	14701	12094	10460	5688	6374	5738	7265	-1.2894118	4.55E-11	9.45E-09
ENSMUSG00000022197	Pdzd2	7715	9045	8952	7705	3936	9048	2595	4833	-1.0308778	0.0002121	0.0070477
ENSMUSG00000002265	Peg3	5068	9663	6568	5900	1862	6151	1879	2963	-1.4123808	1.45E-05	0.0007673
ENSMUSG00000024901	Peli3	466	379	418	360	165	283	202	136	-1.3596598	1.15E-05	0.0006346
ENSMUSG00000027222	Pex16	1720	1883	2140	1513	846	781	795	916	-1.4103164	3.89E-09	5.42E-07
ENSMUSG00000025271	Pfkfb1	1787	2228	1170	1632	351	245	321	448	-2.6119568	3.25E-16	1.65E-13
ENSMUSG00000041229	Phf8	1850	1907	2673	1773	1053	1163	951	1010	-1.2650015	2.62E-08	2.98E-06
ENSMUSG00000026664	Phyh	4422	5175	4880	4007	2584	2522	2575	2908	-1.0972671	9.20E-09	1.18E-06
ENSMUSG00000032462	Pik3cb	2661	2615	2520	2007	1459	1061	1279	1797	-1.0978103	9.13E-06	0.0005168
ENSMUSG00000028756	Pink1	6844	9093	9939	6729	4093	4932	4700	4827	-1.1081428	8.35E-09	1.08E-06
ENSMUSG00000041957	Pkp2	2228	2730	3848	2026	995	868	1013	1067	-1.7420754	3.85E-12	9.53E-10
ENSMUSG00000042632	Pla2g6	1216	1215	1097	857	654	729	531	687	-1.0584236	7.13E-05	0.002878
ENSMUSG00000060675	Plaat3	16130	17663	25961	14900	9224	6037	8343	11756	-1.3525649	3.96E-08	4.31E-06
ENSMUSG00000030546	Plin1	69686	63871	68983	49133	16375	8772	13473	22179	-2.3306154	7.06E-17	3.80E-14
ENSMUSG00000002831	Plin4	98145	129782	155848	95318	20930	24117	24335	31372	-2.5382584	1.13E-30	3.31E-27
ENSMUSG00000073460	Pnldc1	166	164	187	139	85	66	68	87	-1.3868486	1.77E-05	0.0009163
ENSMUSG00000025509	Pnpla2	74044	85158	62184	61280	23806	46369	11679	32912	-1.6218605	1.38E-07	1.30E-05
ENSMUSG00000041653	Pnpla3	9312	5413	2326	4324	2159	286	708	1341	-2.5367178	1.09E-06	8.03E-05
ENSMUSG00000024854	Pold4	675	763	1330	726	475	702	359	450	-1.1103272	0.0003114	0.0094456
ENSMUSG00000045102	Poln	116	120	244	127	62	53	72	41	-1.6891062	9.51E-06	0.0005337
ENSMUSG00000027750	Postn	4836	6371	6342	5352	2774	4543	2319	3035	-1.1593765	4.21E-08	4.57E-06
ENSMUSG00000000440	Pparg	6523	5141	8963	5879	2681	2755	2796	2878	-1.5400888	1.93E-14	7.48E-12
ENSMUSG00000003863	Ppfia3	152	176	171	141	80	79	115	99	-1.0690812	0.0008553	0.0210832
ENSMUSG00000016487	Ppfibp1	4763	5541	6318	4873	2830	5050	2318	2632	-1.0549367	3.33E-06	0.0002151
ENSMUSG00000033526	Ppip5k1	1664	1984	2561	1548	1028	926	788	883	-1.3865191	2.21E-08	2.56E-06
ENSMUSG00000032058	Ppp2r1b	9278	11233	7737	7728	3613	4867	3352	4469	-1.4510013	4.61E-12	1.13E-09
ENSMUSG00000041577	Prelp	17354	18275	42676	17135	5284	5841	4286	5705	-2.4566752	1.49E-18	1.06E-15
ENSMUSG00000002997	Prkar2b	13898	18159	23780	11588	10614	6887	9005	12900	-1.0535884	5.07E-05	0.0021767
ENSMUSG00000044122	Proca1	614	473	672	440	145	158	193	211	-1.9281932	3.53E-10	6.31E-08
ENSMUSG00000037086	Prr32	217	167	942	230	0	3	2	5	-7.4734102	5.62E-22	6.23E-19
ENSMUSG00000036106	Prr5	1106	1399	1240	1110	536	775	715	732	-1.1158479	9.16E-06	0.0005174

ENSMUSG00000021792	Prxl2a	9555	9957	11156	8725	3693	4842	3741	4029	-1.5721456	1.74E-18	1.22E-15
ENSMUSG00000029059	Prxl2b	1008	1023	1467	1095	571	430	435	483	-1.5379848	8.03E-09	1.04E-06
ENSMUSG00000021411	Pxdc1	1355	1333	2163	1186	413	993	348	502	-1.7344926	2.35E-07	2.06E-05
ENSMUSG00000000876	Pxmp4	2469	3232	2842	2516	1532	1508	1481	1755	-1.109459	1.18E-07	1.13E-05
ENSMUSG00000021069	Pygl	4343	5171	4753	4255	2370	2380	1525	2281	-1.4098335	9.10E-12	2.07E-09
ENSMUSG00000043162	Pyurf	639	575	614	539	362	282	286	395	-1.1246677	5.71E-05	0.0023942
ENSMUSG00000043463	Rab9b	54	97	81	92	41	48	32	56	-1.1551392	0.0017957	0.0370032
ENSMUSG00000017491	Rarb	80	102	143	47	33	46	22	43	-1.6681256	0.000149	0.0052619
ENSMUSG00000009281	Rarres2	4752	3836	6952	4672	2711	2929	2830	2646	-1.1480779	8.74E-09	1.12E-06
ENSMUSG00000021708	Rasgrf2	1581	1537	2699	1451	687	788	498	824	-1.6647827	3.92E-09	5.43E-07
ENSMUSG00000044562	Rasip1	1169	1526	1555	960	620	1053	443	829	-1.1333911	0.0002214	0.0072833
ENSMUSG00000040043	Rbms2	4188	5707	4945	4560	2462	2976	2380	2914	-1.1505643	1.32E-09	2.09E-07
ENSMUSG00000032387	Rbpms2	1731	1382	2023	1245	623	498	529	573	-1.81002	1.53E-11	3.37E-09
ENSMUSG00000035504	Reep6	2062	1981	1531	1721	265	277	337	283	-2.9498164	3.00E-28	6.66E-25
ENSMUSG00000012705	Retn	20217	13958	11999	14400	527	456	440	460	-5.3078854	8.16E-98	4.53E-93
ENSMUSG00000061100	Retnla	14217	13535	16351	17382	6634	6365	9476	9866	-1.2041392	2.71E-09	3.94E-07
ENSMUSG00000056666	Retsat	4753	8162	4991	5001	1286	2134	968	1421	-2.2954393	4.44E-18	2.83E-15
ENSMUSG00000020846	Rflnb	4756	3550	3442	3074	2176	3012	1406	2501	-1.0174773	4.03E-05	0.0018045
ENSMUSG00000022018	Rgcc	10101	11692	12829	7777	5266	5208	4808	8086	-1.1505733	7.70E-07	5.94E-05
ENSMUSG00000038530	Rgs4	774	801	974	814	473	653	323	534	-1.0590916	0.0001421	0.0050636
ENSMUSG00000025735	Rhbdl1	158	140	108	131	79	74	61	79	-1.1708965	0.0004023	0.011573
ENSMUSG00000033107	Rnf125	670	896	536	635	211	734	198	311	-1.2512633	0.0016085	0.0340345
ENSMUSG00000038068	Rnf144b	2415	5142	1926	2589	1920	2167	1841	1295	-1.0566309	0.0004141	0.0118684
ENSMUSG00000026269	Rnpepl1	4475	4040	4442	3513	2408	2760	1829	2349	-1.1188965	2.07E-08	2.44E-06
ENSMUSG00000032125	Robo4	3170	3086	3420	2598	1315	1926	820	1414	-1.4724229	4.21E-09	5.81E-07
ENSMUSG00000046215	Rprml	252	434	415	453	98	33	161	100	-2.2464379	3.83E-07	3.19E-05
ENSMUSG00000038387	Rras	4067	3629	5477	3773	2521	2421	2554	2440	-1.0567388	4.65E-08	4.92E-06
ENSMUSG00000051851	Rtl8c	551	626	698	572	282	376	331	326	-1.1913211	3.72E-06	0.0002371
ENSMUSG00000030401	Rtn2	685	876	1367	661	345	441	412	319	-1.5351176	4.37E-07	3.62E-05
ENSMUSG00000044080	S100a1	3804	3252	3297	3281	1669	1499	2267	1909	-1.1829816	3.15E-08	3.49E-06
ENSMUSG00000037936	Scarb1	5681	7762	4399	5821	3063	3747	2076	3089	-1.2898311	2.93E-08	3.29E-06
ENSMUSG00000037071	Scd1	381149	326090	218269	187808	6354	4610	10478	5704	-5.6655938	9.31E-57	1.03E-52
ENSMUSG00000028603	Scp2	21402	28074	25505	21165	11964	10950	12543	13111	-1.276111	1.30E-11	2.90E-09
ENSMUSG00000021904	Sema3g	2237	2475	3072	2739	1319	1965	921	1571	-1.1604153	9.46E-07	7.11E-05

ENSMUSG00000020486	Septin4	1867	1909	1799	1677	955	1033	767	1072	-1.2189352	1.67E-07	1.54E-05
ENSMUSG00000066361	Serpina3c	3698	5857	4396	4453	1511	982	1805	1895	-1.8517138	4.08E-13	1.22E-10
ENSMUSG00000058207	Serpina3k	394	1478	81	240	63	7	18	55	-4.237219	5.41E-07	4.37E-05
ENSMUSG00000062345	Serpinb2	94	93	63	52	53	20	22	30	-1.5631842	0.0006585	0.0171221
ENSMUSG00000021319	Sfrp4	212	249	521	383	116	116	83	81	-2.0564378	7.56E-09	9.91E-07
ENSMUSG00000020354	Sgcd	893	984	1548	837	441	351	568	626	-1.3778895	1.01E-05	0.0005626
ENSMUSG00000031246	Sh3bgrl	10257	11395	15548	9580	6742	6855	6402	6254	-1.1236302	4.85E-09	6.61E-07
ENSMUSG00000037062	Sh3glb1	13361	14082	17664	11034	7266	9744	7786	9294	-1.018	1.77E-07	1.62E-05
ENSMUSG00000026860	Sh3glb2	9316	7713	6874	7777	4547	3308	2888	4478	-1.3474082	9.52E-10	1.54E-07
ENSMUSG00000053617	Sh3pxd2a	25615	28048	18313	25833	10571	12586	7580	13386	-1.4487225	6.38E-11	1.31E-08
ENSMUSG00000036553	Sh3tc1	2376	2464	3267	2305	1308	1623	979	1639	-1.2004896	2.17E-07	1.94E-05
ENSMUSG00000033256	Shf	317	241	264	204	133	146	129	153	-1.1723025	0.0001448	0.0051381
ENSMUSG00000036078	Sigmar1	2444	2192	2667	2189	1389	1240	1295	1427	-1.115199	1.17E-07	1.12E-05
ENSMUSG00000019838	Slc16a10	1756	1587	2190	1818	854	938	1080	973	-1.2215048	6.34E-08	6.52E-06
ENSMUSG00000024935	Slc1a1	139	68	109	121	39	36	62	43	-1.5626145	4.65E-05	0.0020223
ENSMUSG00000005360	Slc1a3	9068	13685	5779	10106	4281	5404	3491	6706	-1.2614519	3.66E-06	0.0002341
ENSMUSG00000046329	Slc25a23	3976	3598	3581	3547	1869	1597	1857	1915	-1.3125384	2.58E-11	5.50E-09
ENSMUSG00000018677	Slc25a39	4026	3990	3954	4036	2474	1965	2146	2335	-1.1299766	2.63E-09	3.85E-07
ENSMUSG00000031808	Slc27a1	18473	25759	13507	22119	9713	18653	6856	9939	-1.141901	2.80E-05	0.0013309
ENSMUSG00000018566	Slc2a4	5120	5500	4595	4235	2848	1848	2884	2604	-1.2229925	3.35E-08	3.66E-06
ENSMUSG00000006641	Slc5a6	890	1761	1019	1837	375	477	407	511	-1.9227822	2.38E-10	4.41E-08
ENSMUSG00000023945	Slc5a7	10	40	1010	42	32	12	36	28	-3.5652114	0.0005858	0.0156129
ENSMUSG00000030108	Slc6a13	2227	2430	3658	2448	668	517	823	941	-2.1408468	1.94E-15	8.73E-13
ENSMUSG00000030495	Slc7a10	8844	6778	7733	5998	626	575	505	588	-3.9757312	2.46E-69	6.83E-65
ENSMUSG00000060681	Slc9a6	2451	4029	2640	2853	1427	1400	1594	1349	-1.3491661	2.77E-09	4.00E-07
ENSMUSG00000072704	Smim10l1	4200	3674	5714	3721	2190	2262	2589	2559	-1.1370172	1.97E-08	2.35E-06
ENSMUSG00000031534	Smim19	1043	1139	1322	1066	607	746	690	650	-1.0569386	1.01E-05	0.0005639
ENSMUSG00000045709	Smkr-ps	260	279	144	239	117	139	176	127	-1.0259307	0.0024947	0.0471952
ENSMUSG00000019872	Smpdl3a	2712	3054	4164	2931	1461	1823	1647	1527	-1.2849175	2.16E-10	4.04E-08
ENSMUSG00000023064	Sncg	15919	5954	28877	10370	2529	2068	2926	4413	-2.6231209	1.52E-11	3.37E-09
ENSMUSG00000060429	Sntb1	2756	5137	3697	2547	1413	1280	1052	3067	-1.3416165	7.98E-05	0.0031702
ENSMUSG00000038301	Snx10	1121	1695	1620	1744	723	1006	878	825	-1.1387563	1.82E-06	0.0001259
ENSMUSG00000025006	Sorbs1	10296	14006	20871	9173	3529	5945	2779	5576	-1.9063187	2.80E-11	5.92E-09
ENSMUSG00000025902	Sox17	1291	1570	1530	1188	684	1273	495	872	-1.0596941	0.0003121	0.0094619

ENSMUSG00000046470	Sox18	3382	3185	3123	2937	1731	2473	1119	1831	-1.1271765	1.30E-06	9.32E-05
ENSMUSG00000063060	Sox7	1042	1906	1695	1436	533	666	410	731	-1.6686704	7.18E-09	9.51E-07
ENSMUSG00000029699	Ssc4d	92	114	111	130	66	66	68	59	-1.0711785	0.0006498	0.0169599
ENSMUSG00000030255	Sspn	2467	2544	3719	2745	1518	1686	1572	1590	-1.1360108	1.98E-08	2.36E-06
ENSMUSG00000028538	St3gal3	1571	1702	1791	1598	822	623	795	870	-1.3821185	1.37E-08	1.69E-06
ENSMUSG00000020303	Stc2	845	931	1007	1052	507	403	557	602	-1.167581	9.56E-06	0.0005355
ENSMUSG00000026027	Stradb	932	964	1275	1044	582	639	527	623	-1.117026	5.60E-06	0.0003381
ENSMUSG00000027762	Sucnr1	1969	2332	2527	1606	587	661	775	892	-1.8258035	2.58E-12	6.67E-10
ENSMUSG00000031357	Syap1	5947	7007	8959	5183	3256	3060	3403	3649	-1.3075461	3.75E-10	6.63E-08
ENSMUSG00000020059	Sycp3	200	284	383	158	147	130	138	137	-1.1816881	0.0007705	0.0194326
ENSMUSG00000050315	Synpo2	6379	9707	12811	4933	5293	4566	2661	4674	-1.2672046	1.32E-05	0.0007088
ENSMUSG00000031144	Syp	400	327	721	265	161	164	163	148	-1.7171478	1.40E-06	9.93E-05
ENSMUSG00000030731	Syt3	200	112	104	184	97	92	73	68	-1.1574382	0.0012606	0.0282718
ENSMUSG00000024743	Syt7	510	595	529	459	326	411	179	271	-1.1255689	0.0002853	0.0088538
ENSMUSG00000043866	Taf10	2050	2007	1989	540	474	521	627	929	-1.6779324	3.07E-05	0.0014377
ENSMUSG00000032419	Tbx18	233	247	204	230	95	106	79	101	-1.5585925	2.32E-07	2.04E-05
ENSMUSG00000036667	Tcaf1	4726	4303	6042	4088	2154	1852	1831	1920	-1.5919131	1.93E-15	8.73E-13
ENSMUSG00000068079	Tcf15	562	788	1021	388	176	134	192	394	-1.9007819	5.66E-06	0.0003411
ENSMUSG00000035517	Tdrd7	2137	2715	3095	2476	1628	1361	1283	1377	-1.1679441	3.91E-08	4.26E-06
ENSMUSG00000050052	Tdrp	2432	2597	3383	2853	1127	1103	1091	1663	-1.4552006	1.91E-10	3.66E-08
ENSMUSG00000040152	Thbs1	6526	7212	10519	5083	1268	4180	827	1448	-2.2594792	3.84E-09	5.38E-07
ENSMUSG00000035686	Thrsp	13550	17630	8787	8731	4308	2518	4582	5936	-1.7868264	7.54E-09	9.91E-07
ENSMUSG00000030317	Timp4	5005	6307	9442	7586	3941	3795	4252	3712	-1.1278949	1.20E-08	1.50E-06
ENSMUSG00000054452	Tle5	6149	5569	7174	5524	3727	3770	3943	3310	-1.0192392	1.53E-08	1.88E-06
ENSMUSG00000063406	Tmed5	2680	3519	4919	2816	1722	3197	1508	1847	-1.0576501	3.69E-05	0.0016818
ENSMUSG00000039886	Tmem120a	5618	5420	9605	4749	2319	1596	1555	2761	-1.8999808	2.93E-12	7.45E-10
ENSMUSG00000054434	Tmem120b	2597	3712	2864	2203	849	1046	642	1284	-1.8758135	6.92E-12	1.62E-09
ENSMUSG00000024845	Tmem134	6992	7730	8159	6974	2420	3395	2376	2211	-1.8240166	1.98E-22	2.39E-19
ENSMUSG00000057137	Tmem140	612	613	553	532	265	625	249	251	-1.0589414	0.0014716	0.0319467
ENSMUSG00000019158	Tmem160	615	608	961	458	448	363	301	417	-1.0752344	0.0005337	0.0145033
ENSMUSG00000057716	Tmem178b	162	154	157	147	69	120	69	93	-1.1254132	0.0004469	0.0125943
ENSMUSG00000079588	Tmem182	3051	3412	5228	2302	793	1189	969	1264	-2.0268077	5.52E-14	2.00E-11
ENSMUSG00000030431	Tmem238	339	236	260	180	114	93	124	184	-1.2732672	0.0005012	0.0138159
ENSMUSG00000079659	Tmem243	1010	938	1104	904	590	611	550	485	-1.1184137	6.62E-06	0.0003898

ENSMUSG00000041737	Tmem45b	6348	3426	5086	2222	142	37	97	102	-5.782779	6.71E-36	2.66E-32
ENSMUSG00000073680	Tmem88b	775	1082	619	612	88	106	107	80	-3.3292976	4.28E-23	5.28E-20
ENSMUSG00000063727	Tnfrsf11b	460	189	359	250	76	87	71	83	-2.2890099	5.68E-10	9.73E-08
ENSMUSG00000000934	Top1mt	363	385	314	375	215	213	198	222	-1.0538158	0.0001305	0.0047106
ENSMUSG00000023456	Tpi1	6521	6621	9911	7752	4425	3545	4917	4655	-1.0874557	4.72E-08	4.98E-06
ENSMUSG00000029030	Tprgl	4534	4819	6662	4173	2750	3319	2692	3169	-1.0515796	9.63E-08	9.44E-06
ENSMUSG00000020993	Trappc6b	2927	3419	4509	3014	1661	1820	1992	1805	-1.2186041	2.66E-09	3.87E-07
ENSMUSG00000046275	Trarg1	12078	13056	22162	8625	1612	2271	1638	2881	-3.0261171	8.88E-25	1.30E-21
ENSMUSG00000039853	Trim14	896	997	1141	530	430	561	318	483	-1.2999532	4.21E-05	0.0018737
ENSMUSG00000018507	Trpv2	763	1185	1110	878	491	886	289	411	-1.2371392	0.0002204	0.007259
ENSMUSG00000020963	Tshr	5861	5570	5527	5187	2414	1245	2402	2854	-1.5907539	4.60E-10	8.06E-08
ENSMUSG00000029669	Tspan12	6507	6216	6279	5479	2912	2516	3315	3148	-1.3328723	6.93E-12	1.62E-09
ENSMUSG00000020577	Tspan13	2293	1999	2539	1962	1284	1525	1150	1159	-1.0793902	4.74E-07	3.88E-05
ENSMUSG00000025875	Tspan17	1439	1552	2523	1556	1070	917	959	911	-1.1545033	4.33E-06	0.0002716
ENSMUSG00000058254	Tspan7	3725	3398	3873	3308	2114	1999	1574	1850	-1.2176851	5.48E-10	9.44E-08
ENSMUSG00000041736	Tspo	7989	9174	11467	8547	4923	6325	4964	5478	-1.0706876	2.41E-09	3.57E-07
ENSMUSG00000033530	Ttc7b	1649	1788	2303	1477	973	958	844	1187	-1.1532006	3.16E-06	0.000205
ENSMUSG00000002550	Uck1	5603	5011	3156	4298	2300	1816	1823	2229	-1.4444263	8.05E-10	1.33E-07
ENSMUSG00000032942	Ucp3	1154	1638	1492	776	202	204	121	236	-3.0304431	5.66E-18	3.53E-15
ENSMUSG00000090145	Ugt1a6b	359	473	660	440	90	190	301	340	-1.3489097	0.0007341	0.0187186
ENSMUSG00000029591	Ung	168	206	184	299	84	178	74	126	-1.1862038	0.0008971	0.0218991
ENSMUSG00000026839	Upp2	188	129	139	97	47	76	75	49	-1.4776629	6.78E-05	0.0027664
ENSMUSG00000034911	Ushbp1	4111	4820	4666	4274	2499	3036	1517	2454	-1.2100253	1.99E-08	2.36E-06
ENSMUSG00000035713	Usp35	1044	698	537	1045	309	550	286	279	-1.5357506	2.97E-06	0.0001942
ENSMUSG00000021256	Vash1	433	301	353	203	159	191	126	191	-1.2615378	0.0002417	0.0077827
ENSMUSG00000023951	Vegfa	8088	9624	7966	10461	5122	6661	4430	5497	-1.0305437	1.87E-08	2.27E-06
ENSMUSG00000024962	Vegfb	4027	3232	3387	2712	1013	871	1147	1172	-1.9633609	5.29E-17	2.93E-14
ENSMUSG00000091243	Vgll3	1302	1504	3068	1128	521	619	487	614	-1.9290363	1.86E-09	2.81E-07
ENSMUSG00000001943	Vsig2	433	449	366	385	240	280	161	206	-1.1851343	5.90E-05	0.0024629
ENSMUSG00000020101	Vsir	7551	8128	9722	6864	3945	5968	3398	4940	-1.1239309	4.23E-08	4.58E-06
ENSMUSG00000051748	Wfdc21	141	75	158	120	38	47	55	32	-1.8104111	1.48E-06	0.0001045
ENSMUSG00000028639	Ybx1	13640	14704	17226	9488	7566	8383	7442	9324	-1.0490466	6.89E-07	5.37E-05
ENSMUSG00000042675	Ypel3	3729	3616	3840	3589	1724	3321	1873	2068	-1.0274017	1.29E-06	9.26E-05
ENSMUSG00000034059	Ypel4	143	121	141	144	78	108	57	78	-1.0722026	0.0008948	0.0218629

ENSMUSG00000039770	Ypel5	2813	4156	3424	3269	1719	2421	1985	1873	-1.0735429	1.06E-07	1.03E-05
ENSMUSG00000018326	Ywhab	20040	23122	25419	20437	12395	10217	11810	13055	-1.1915063	1.25E-10	2.49E-08
ENSMUSG00000006310	Zbtb32	104	115	132	98	41	115	39	42	-1.2435034	0.0023326	0.0448944
ENSMUSG00000020335	Zfp354b	115	126	159	140	78	66	81	70	-1.1521965	0.0002314	0.0075476
ENSMUSG00000050919	Zfp366	911	1345	1172	789	557	833	335	621	-1.1570913	0.0002774	0.0086767
ENSMUSG00000078866	Zfp970	1132	1820	1718	1405	683	926	770	764	-1.2471779	5.39E-07	4.36E-05
ENSMUSG00000037224	Zfyve28	127	136	533	156	70	131	48	97	-1.7407061	0.0004926	0.0136392
ENSMUSG00000002266	Zim1	78	340	202	187	44	132	28	41	-2.0481263	0.000123	0.0045071
ENSMUSG00000034645	Zyg11a	164	362	209	621	159	140	99	175	-1.5017706	0.0003322	0.0099309
