# Supplementary material for: Adipocyte RNF20 Knockout Leads to Hyperinsulinemia via the H2Bub‐H3K4me3‐Slc2a4 Axis
Source: J Cell Mol Med. 2025 Jun 16;29(11):e70649. doi: 10.1111/jcmm.70649 (PMC12168218; doi:10.1111/jcmm.70649)
Supplement: Supplementary file 4 — Table S3 ChIP seq analysis of DRGs in siNC and siRNF20 cells. [file JCMM-29-e70649-s002.pdf]

Geneid	NC_1_IP	NC_2_IP	NC_3_IP	si_RNF20_1_I	si_RNF20_2_I	si_RNF20_3_I	logFC	logCPM	PValue	FDR
Xiap	0.3958003	0.28734502	0.21201	0.08329824	0.16362533	0.158690339	-1.24915	-0.1380661	0.00132	0.023625546
Adipor2	2.801578	1.91337981	1.702162	0.85547289	1.20047566	1.083366736	-1.14715	2.70694316	0.00595	0.061041078
Gm13398	0.3088963	0.25353973	0.234601	0.14493893	0.14025029	0.103759068	-1.1455	-0.2754049	4.3E-05	0.00276173
Slc25a34	0.9981052	0.79780501	0.934929	0.42815294	0.5776976	0.405881059	-1.0578	1.49447855	9.3E-07	0.000284413
Gm15598	0.7881589	0.68624753	0.76984	0.35651645	0.43744732	0.390622373	-1.02488	1.2235598	2.6E-07	0.000125558
Slc2a4	2.7551145	2.30214072	2.723112	1.19782864	1.57614606	1.341238537	-1.02209	3.007753	1.3E-08	2.32136E-05
Hcar1	1.3044202	1.09698189	1.46843	0.60641116	0.70235339	0.755304978	-1.00241	2.00364948	2.1E-08	2.92814E-05
Sema3g	1.2700028	1.07162791	1.299865	0.51311714	0.83148383	0.616450932	-0.99596	1.92390326	3.2E-06	0.000568769
Gpt	1.440369	1.25698236	1.736049	0.61973888	0.9784127	0.799555169	-0.98336	2.20330035	1.1E-06	0.000302362
Mrap	2.7189762	2.40693714	2.66865	1.1245262	1.72250725	1.467885634	-0.95761	3.03319447	1.1E-05	0.001245721
Gng3	0.4026838	0.38876091	0.507434	0.24156489	0.25879517	0.216673347	-0.95484	0.46013712	4E-06	0.000647633
Cmbl	1.1839593	0.93978725	0.931454	0.48979363	0.6027423	0.60729572	-0.95351	1.69573025	4.2E-06	0.000658865
Mir7225	0.2718976	0.21297337	0.284997	0.12328139	0.15694675	0.14648339	-0.94747	-0.2738708	5.8E-05	0.003278694
Gpd1	4.3951046	4.09044093	4.884919	2.01748329	2.97197033	2.47343307	-0.94254	3.81095656	3.4E-06	0.000586424
Ffar4	0.4577517	0.39214144	0.389264	0.22323927	0.27048269	0.204466398	-0.93601	0.41280963	3E-05	0.002195936
G0s2	0.8914112	0.78343776	0.761151	0.37983996	0.56016632	0.434872563	-0.93445	1.37725834	8.7E-06	0.00104744
Nisch	1.0084304	0.88569878	0.9106	0.42315504	0.67787638	0.482174491	-0.93358	1.57731634	1.9E-05	0.001678617
Scd3	0.3252446	0.29410608	0.312802	0.16326454	0.18199144	0.184630106	-0.91538	0.01203507	2.6E-05	0.002075885
Cox7a1	0.5369118	0.35495562	0.413593	0.22157331	0.30053633	0.22430269	-0.91358	0.49312309	0.00014	0.005542579
Ceacam1	0.3728439	0.33974323	0.378247	0.1982498	0.26380411	0.164275018	-0.9072	0.23730727	7.5E-05	0.003855228
Klb	1.4575777	0.99387573	0.788955	0.55643222	0.72128718	0.604243983	-0.90273	1.80987846	0.02023	0.130768254
Gm11827	1.0262242	0.90571152	0.911747	0.58308766	0.54764397	0.524135879	-0.88779	1.61398421	1.1E-06	0.000302362
Rdh16	0.3837542	0.27382291	0.25893	0.15326876	0.20369684	0.177000763	-0.887	0.00603576	0.00047	0.011672126
Aldh3b2	1.1134035	0.8941501	0.896107	0.49812346	0.64391577	0.565578471	-0.87452	1.6499914	7.8E-06	0.000971916
Grb7	0.2546889	0.20621231	0.260668	0.15326876	0.14525922	0.12359536	-0.87452	-0.3364142	0.00015	0.005803292
Irs3	0.3028733	0.33805297	0.42402	0.18991998	0.24376835	0.189207712	-0.87147	0.20672186	4.3E-05	0.00276741
Gm16163	1.0290809	0.87555719	0.96621	0.49645749	0.61109053	0.588985296	-0.86238	1.63241304	1.1E-06	0.000302362
Hcar2	0.5592831	0.49693786	0.596061	0.25822453	0.3355989	0.386044767	-0.85029	0.84141435	2E-05	0.001733389
Mir3104	0.2718976	0.22649549	0.239815	0.12661332	0.17364321	0.140379915	-0.84812	-0.2907137	0.0003	0.008759363
Gm28046(Rdh	0.1652036	0.13353092	0.113825	0.0849642	0.10017878	0.062560614	-0.8475	-1.0902364	0.00434	0.049942251
Selenbp1	0.6814649	0.62708826	0.722329	0.33985681	0.43911696	0.431820826	-0.84608	1.13918384	3.4E-06	0.000586424
Ghrh	0.3521935	0.36509721	0.354509	0.21657542	0.23708977	0.192259449	-0.83659	0.23956529	5.5E-05	0.003181669
Tenm4	1.094474	0.90598196	1.042672	0.58975152	0.61276018	0.631709618	-0.8325	1.72557601	5.9E-07	0.000231697

Cox6a2	0.2495263	0.21297337	0.163352	0.12161543	0.14358958	0.115966017	-0.82598	-0.5041684	0.00189	0.030111376
U6	0.227155	0.19438046	0.194632	0.10995367	0.12355382	0.143431652	-0.80714	-0.5290006	0.00071	0.015517914
Tex19.1	0.3407325	0.35833615	0.378838	0.21324349	0.25378623	0.204466398	-0.78603	0.26222065	7.2E-05	0.003794843
Gm12426	0.3854751	0.41918568	0.378838	0.2265712	0.25378623	0.259397669	-0.782	0.40034509	7.9E-05	0.003966451
Mirlet7b	0.3080359	0.25861052	0.304113	0.15326876	0.2237326	0.175993689	-0.7569	-0.0292856	0.00044	0.011194679
Gm4651	0.547237	0.55609713	0.736822	0.33652488	0.47918847	0.349423919	-0.75671	1.02422118	5.8E-05	0.003278694
Gm45416	0.3166403	0.33467244	0.371886	0.21990735	0.20703614	0.222776822	-0.75352	0.19776417	7.9E-05	0.003958536
Gm18258	0.5110987	0.32115032	0.331917	0.27321822	0.26380411	0.210569873	-0.75004	0.39510647	0.00274	0.038137009
Gm45372	0.7158823	0.69300859	0.743773	0.39316768	0.49421529	0.486752097	-0.74946	1.25974294	9.1E-06	0.001076351
Prpsap1	0.3131985	0.29410608	0.326704	0.15826665	0.24376835	0.198362924	-0.73909	0.07603494	0.00044	0.011194679
Itih4	0.4611934	0.5865219	0.623866	0.31320137	0.37066147	0.396725847	-0.72682	0.90249285	6.5E-05	0.003539332
Psen2	1.4532756	1.32347737	1.129562	0.74968413	0.92080991	0.891107287	-0.72018	2.13560764	0.00013	0.005281976
Gm43598	0.3372907	0.26368132	0.312802	0.17992419	0.20369684	0.210569873	-0.72014	0.05257153	0.00039	0.010391624
Gm23794	0.2374802	0.17240701	0.152925	0.10662174	0.11353595	0.14648339	-0.71805	-0.6175148	0.00685	0.066857922
Rprml	0.5885379	0.41918568	0.608225	0.29654172	0.42075086	0.338742839	-0.71229	0.85874595	0.00029	0.008533901
Gm23221	0.2718976	0.19607072	0.264144	0.12661332	0.1803218	0.170897288	-0.71205	-0.2595425	0.00152	0.025856303
Gm25559	0.2512472	0.19607072	0.215486	0.13994104	0.1803218	0.115966017	-0.71135	-0.3904746	0.00285	0.038995151
Cidec	3.1784488	2.6638574	2.938598	1.5543451	2.2707189	1.959215337	-0.70781	3.29595437	0.00038	0.010282688
Gm13012	0.2082254	0.25692026	0.222437	0.12994525	0.17364321	0.149535127	-0.70595	-0.3373017	0.00161	0.027069473
9030619P08R	0.5610039	0.51722104	0.476154	0.34652066	0.40405439	0.279233962	-0.70545	0.82061078	0.00046	0.011450324
Gm17151	0.3424533	0.24170787	0.389264	0.15826665	0.25044694	0.231932034	-0.69613	0.14087139	0.00168	0.02790358
Cpsf4l	0.2650141	0.26875211	0.366673	0.14327297	0.25044694	0.199888792	-0.69607	0.0313618	0.00158	0.026741081
1700029B24R	0.320082	0.27382291	0.205059	0.21324349	0.11687524	0.202940529	-0.68881	-0.1114522	0.01432	0.10658167
4930455H04R	0.2908272	0.34988482	0.198108	0.19325191	0.22039331	0.152586864	-0.68626	-0.0351904	0.01266	0.097518702
Cfd	1.9153294	1.96577802	2.519791	1.24280969	1.52772632	1.489247795	-0.68289	2.83898641	1.6E-07	8.7946E-05
RP23-312B17.	0.4577517	0.42256621	0.39274	0.25322664	0.3105542	0.294492648	-0.67506	0.5456783	0.0004	0.010567433
Ldb3	0.3166403	0.24339814	0.311064	0.17659226	0.19367897	0.21362161	-0.67364	0.00011744	0.00076	0.016377312
Fam209	0.359662	0.29917688	0.370149	0.19991577	0.24042906	0.250242457	-0.67271	0.23540768	0.00039	0.010391624
Gm4978	0.2632933	0.22480522	0.201583	0.15660069	0.16696463	0.143431652	-0.6703	-0.315143	0.00294	0.039651952
Adig	2.0048147	1.73928253	2.11082	1.15784549	1.38580639	1.403799151	-0.66865	2.72244697	3.8E-07	0.000157134
A530016L24R	1.7690554	1.65814981	2.113149	1.0962048	1.25390434	1.379385253	-0.66699	2.63938648	3.4E-07	0.000151655
Tmod4	0.2202715	0.27382291	0.271095	0.14327297	0.19367897	0.1800525	-0.6656	-0.1771203	0.00145	0.025004468
Gm33280	0.3699873	0.26706185	0.337131	0.16826244	0.28383986	0.209044004	-0.66193	0.16490799	0.00293	0.039648202
Elovl3	0.2770602	0.25749495	0.281522	0.1939183	0.17474518	0.184630106	-0.66108	-0.0834235	0.0007	0.015293435

E030018B13R	0.2959898	0.32453085	0.357984	0.21990735	0.24376835	0.201414661	-0.65867	0.17117859	0.00043	0.011027668
Prr5	0.3699873	0.35157509	0.404904	0.25322664	0.28217022	0.231932034	-0.65596	0.37208632	0.00028	0.008409503
Mirlet7c-2	0.3114777	0.2450884	0.338868	0.2032477	0.22039331	0.185148901	-0.65559	0.04432949	0.00082	0.017312852
Rhbdl1	0.5833753	0.61187587	0.78548	0.38150592	0.41741156	0.541683368	-0.65465	1.16645355	0.00014	0.005442829
Gm37391	0.3613829	0.27382291	0.184205	0.17992419	0.23041118	0.155638602	-0.65234	-0.0553012	0.02839	0.159920271
Gm19024	0.4371012	0.44792018	0.326704	0.22990313	0.30387562	0.300596123	-0.64883	0.49194944	0.00265	0.037440867
Gm12795	0.3372907	0.25522999	0.198108	0.18658805	0.20035755	0.158690339	-0.64883	-0.1085432	0.01014	0.08521823
Astl	0.146274	0.20198665	0.179861	0.13411016	0.11437077	0.113677214	-0.64717	-0.6839242	0.00561	0.058609606
Gm20636	0.3613829	0.40904409	0.427496	0.23323506	0.28717916	0.299070254	-0.6468	0.46191725	0.00034	0.009668226
Gm16180	0.4887274	0.33974323	0.354509	0.2032477	0.33726854	0.274656356	-0.64466	0.4536778	0.00561	0.058613346
B430010I23Ri	0.5833753	0.49355734	0.47094	0.28987786	0.3790097	0.396725847	-0.6445	0.83551112	0.00084	0.017521593
Cox8b	0.3131985	0.26706185	0.284997	0.15993261	0.22039331	0.21362161	-0.64385	0.00663805	0.00163	0.027373848
Gm26541	0.5902588	0.48679628	0.69859	0.34818663	0.42742944	0.439450169	-0.64122	1.01673558	0.00015	0.005639819
Tusc5	2.2922002	2.07395497	1.967592	1.20699145	1.80258349	1.383260959	-0.63855	2.8580122	0.00305	0.040300607
Gm26347	0.2994316	0.24001761	0.323228	0.21324349	0.19534861	0.186155974	-0.6349	0.00044279	0.00107	0.020342561
Gm10687	3.7497779	3.3129191	4.080324	2.3006973	2.72987162	2.661114913	-0.63443	3.661028	2.7E-08	3.0727E-05
Mir1904	3.7770365	2.88808793	3.346109	2.24182211	2.42850048	2.28269949	-0.63071	3.51492395	3E-05	0.002195936
Gm42732	0.3441742	0.31438926	0.324966	0.2032477	0.25378623	0.225828559	-0.62996	0.19388238	0.00087	0.017876572
Mcam	3.6878266	3.24868903	4.035142	2.29070151	2.69981799	2.615338854	-0.62804	3.64093675	2.7E-08	3.0727E-05
Mettl7b	0.4147299	0.3752388	0.430971	0.21490945	0.31723279	0.31432894	-0.62746	0.49618644	0.00091	0.018439579
Gm45643	0.2288758	0.3318666	0.24329	0.14707137	0.18533073	0.227354428	-0.62426	-0.0846839	0.00799	0.074127758
Gm15621	0.320082	0.35664588	0.366673	0.19991577	0.29051845	0.23650964	-0.62402	0.27690539	0.00114	0.021364
Apoc3	0.3235237	0.3076282	0.349295	0.21990735	0.21204507	0.251768326	-0.61732	0.18957313	0.00066	0.014811991
Rorc	2.1115087	1.79675153	2.213941	1.21948619	1.60786934	1.455678685	-0.61618	2.8071344	3.2E-06	0.000568769
Npas2	0.2787811	0.24339814	0.33018	0.17992419	0.21371472	0.201414661	-0.61435	-0.0112205	0.00112	0.021140174
Gm12218	0.4577517	0.32284059	0.436185	0.27988208	0.29051845	0.283811568	-0.60956	0.49820299	0.00091	0.018439579
Manba	0.4818439	0.58145111	0.609963	0.30653751	0.490876	0.381467161	-0.60739	0.95221977	0.00106	0.020342561
Htra4	0.2650141	0.27720343	0.333655	0.22323927	0.21371472	0.183104237	-0.59831	0.03635599	0.0014	0.024448094
Gm16575	0.8655981	0.73526521	1.061788	0.5764238	0.64448345	0.656123516	-0.59814	1.61200006	3.8E-05	0.002579112
Gm15235	0.1961793	0.22311496	0.236339	0.13994104	0.13190205	0.19073358	-0.59594	-0.3691206	0.00527	0.05624387
Gm45187	0.4766813	0.5527166	0.500483	0.33819084	0.43076873	0.323484152	-0.59397	0.83742962	0.00096	0.018997375
Stxbp2	0.7571832	0.72681388	0.69859	0.37650803	0.57101902	0.60729572	-0.59375	1.34303472	0.00132	0.02366855
Gm13071	0.2977107	0.22987602	0.24329	0.19158594	0.19200932	0.167845551	-0.58884	-0.1307327	0.0045	0.05126324
Gm44157	0.3372907	0.34481403	0.340606	0.22990313	0.26046482	0.241087246	-0.58721	0.26600456	0.001	0.019546667
